# MprF from *Pseudomonas aeruginosa* is a promiscuous lipid scramblase with broad substrate specificity

**DOI:** 10.1126/sciadv.ads9135

**Published:** 2025-04-09

**Authors:** Matthew T. K. Hankins, Matyas Parrag, Alisa A. Garaeva, Jennifer C. Earp, Markus A. Seeger, Phillip J. Stansfeld, Maike Bublitz

**Affiliations:** ^1^ Department of Biochemistry, University of Oxford, South Parks Road, Oxford OX1 3QU, UK.; ^2^ School of Life Sciences and Department of Chemistry, University of Warwick, Coventry CV4 7AL, UK.; ^3^ Institute of Medical Microbiology, University of Zurich, Gloriastrasse 28/30, 8006 Zurich, Switzerland.; ^4^ Institute of Translational Medicine, Faculty of Medical Sciences, Private University in the Principality of Liechtenstein (UFL), Dorfstrasse 24, 9495 Triesen, Liechtenstein.

## Abstract

The multiple peptide resistance factor (MprF) is a bifunctional membrane protein found in many bacteria, including *Pseudomonas aeruginosa* and *Staphylococcus aureus*. MprF modifies inner leaflet lipid headgroups through aminoacylation and translocates modified lipid to the outer leaflet. This activity provides increased resistance to antimicrobial agents. MprF presents a promising target in multiresistant pathogens, but structural information is limited and both substrate specificity and energization of MprF-mediated lipid transport are poorly understood. Here, we present the cryo-EM structure of MprF from *P. aeruginosa* (*Pa*MprF) bound to a synthetic nanobody. *Pa*MprF adopts an “open” conformation with a wide, lipid-exposed groove on the periplasmic side that induces a local membrane deformation in molecular dynamics simulations. Using an in vitro liposome transport assay, we demonstrate that *Pa*MprF translocates a wide range of different lipids without an external energy source. This suggests that *Pa*MprF is the first dedicated lipid scramblase to be characterized in bacteria.

## Teaser

Structural and functional characterization of MprF from *Pseudomonas aeruginosa* reveals that it is a bacterial lipid scramblase.

## INTRODUCTION

**Fig. 1.**
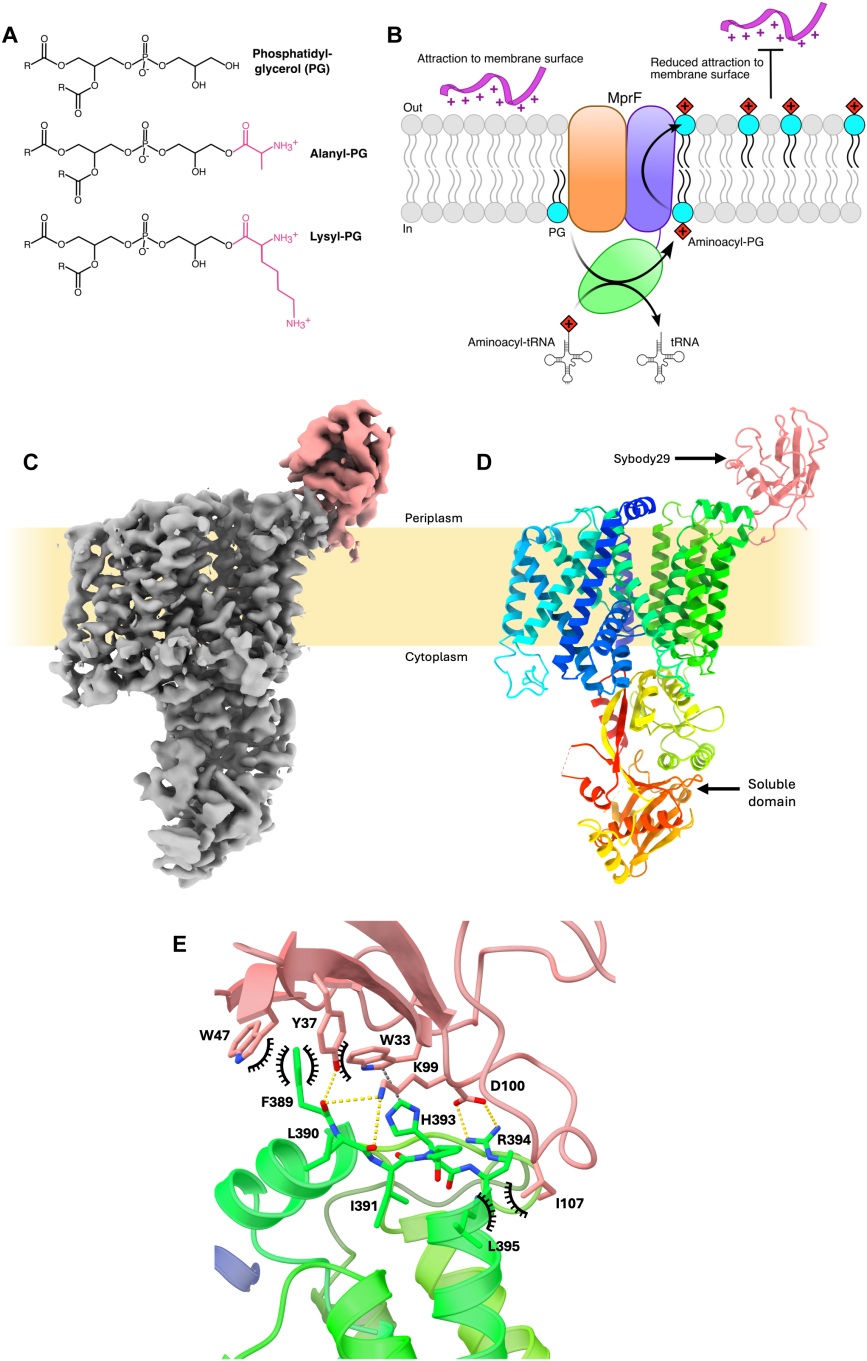
**Substrate, products and activity of MprF, and** *Pa*MprF-Sb29 complex model. (**A**) Skeletal formulae of PG and common amino acid modifications of PG found in bacteria. (**B**) Schematic of MprF activity showing modification of PG (cyan) to aminoacyl-PG in the inner leaflet of the bilayer, followed by translocation of aminoacyl-PG to the outer leaflet, resulting in reduced attraction of CAMPs (pink) to the membrane surface. (**C**) Final post-processed cryo-EM map of *Pa*MprF (gray) bound to Sb29 (pink). (**D**) Side view of the complex in cartoon representation, *Pa*MprF colored from N to C terminus as a rainbow and Sb29 colored as pink. (**E**) View of the interaction surface between *Pa*MprF TMD2 and Sb29, colored as in (D). Polar interactions are shown as yellow dashes, cation/π interactions as gray dashes, and hydrophobic contacts as black arcs.

The ability of bacteria to chemically modify surface-exposed lipids is a key defense mechanism that provides many common pathogens with resistance to antimicrobials and environmental stressors. One such lipid modification is the addition of an amino acid residue to the head group of a phosphatidylglycerol (PG) molecule in the plasma membrane (Fig. 1A). This modification provides bacteria with resistance to cationic antimicrobial peptides (CAMPs) and other external challenges such as low pH (*1*–*3*). Studies in model membranes have demonstrated that this modification reduces the electrostatic attraction of positively charged antimicrobials to the bacterial plasma membrane as a direct effect of the reduction in anionic character of PG (*4*). First identified in *Staphylococcus aureus* and *Clostridium welchii* (*5*), the presence of aminoacyl-PG is now recognized to be widespread across both Gram-positive and Gram-negative bacteria and even some archaea (*6*). In the opportunistic Gram-negative pathogen *Pseudomonas aeruginosa*, despite the presence of an outer membrane, the formation of aminoacyl-PG in the inner membrane has been shown to mediate resistance to the cationic peptide protamine sulfate, the antibiotic cefsulodin, and the heavy metal ion Cr^3+^ (*3*). The protein responsible for this lipid modification is the multiple peptide resistance factor (MprF) (*1*), a bifunctional transmembrane (TM) protein made up of distinct “synthase” and “flippase” domains (*7*). The soluble synthase domain binds a charged transfer RNA and aminoacylates PG in the inner leaflet of the bacterial inner membrane (*8*–*11*), and the TM flippase domain translocates this modified lipid to the outer leaflet (*7*, *12*, *13*) (Fig. 1B).

MprF represents a promising target to combat antimicrobial resistance, as removal of MprF has been shown to sensitize bacteria to positively charged antimicrobials (*1*, *14*). Furthermore, daptomycin resistance in many clinical isolates of methicillin-resistant *S. aureus* (MRSA) has been attributed to a variety of gain-of-function mutations at the *mprF* locus (*15*–*17*), and the recent use of antibodies to target the MprF flippase domain sensitized such MRSA strains to host CAMPs (*18*). Despite these findings, little is known about the molecular mechanism of MprF-mediated lipid transport. The only published full-length structure of MprF is from the soil-bacterium *Rhizobium tropici* (*19*), which has limited sequence identity to MprF homologs from pathogenic species such as *S. aureus* and *P. aeruginosa.* No in vitro assessment of lipid transport has yet been reported for MprF, leaving both the energization and specificity of the lipid transport mechanism unclear.

The structure of MprF from *R. tropici* (*Rt*MprF) (*19*) revealed the architecture of the flippase domain that shares no homology with any previously characterized lipid transporters. Two aminoacyl-PG binding sites were identified in *Rt*MprF: one accessible to the cytoplasm and one to the periplasm. These two sites are separated by a conserved salt bridge, leading to the suggestion that the proton motive force (PMF) could energize lipid transport by protonation and subsequent conformational change to open a channel between the two lipid-binding sites. However, there is no additional structural or functional data to support this hypothesis.

To provide further insight into the molecular mechanism of MprF-mediated lipid transport, we determined the cryo–electron microscopy (cryo-EM) structure of MprF from the Gram-negative pathogen *P. aeruginosa* (*Pa*MprF). We also assessed *Pa*MprF’s in vitro lipid transport activity in liposomes and conducted molecular dynamics (MD) simulations to further characterize its interaction with the membrane lipid environment. The structural, functional, and computational data suggest that *Pa*MprF passively transports a vast variety of lipid species, probably via a PMF-independent route of lipid translocation involving a local destabilization of the bilayer. Thus, *Pa*MprF displays hallmark characteristics of a lipid scramblase.

## RESULTS

### The cryo-EM structure of *Pa*MprF

Full-length *Pa*MprF was purified following recombinant production in *Escherichia coli* and eluted predominantly as a monomer from size exclusion chromatography (SEC) (fig. S1A). Given the small size of monomeric *Pa*MprF (96 kDa), synthetic nanobodies (sybodies) (*20*) were generated against purified full-length *Pa*MprF to isolate specific binders that could act as fiducial markers for single-particle cryo-EM structural determination. A truncated form of *Pa*MprF representing the TM region was used in pull-down assays to identify a sybody binding to the TM region (Sb29), which was then utilized for structure determination. *Pa*MprF was reconstituted into SaposinA-lipid nanoparticles (SapNPs) (*21*, *22*) composed of phosphatidylcholine (PC):PG at a 7:3 molar ratio, and the structure of the *Pa*MprF-Sb29 complex was determined to 3.28-Å resolution (Fig. 1, C and D).

Sb29 binds at a surface-exposed amphipathic helix on the periplasmic face of *Pa*MprF (Fig. 1E). The buried surface area for this interaction is ~680 Å^2^, similar to previously reported areas of sybody binding interfaces (*23*). Most of the TM region of *Pa*MprF is well resolved (fig. S1G), with the lowest resolution located around TM helix 5. This helix makes up part of the dimer interface in *Rt*MprF. While the structure of *Pa*MprF presented here is monomeric, a dimeric population of *Pa*MprF is present in early stages of purification (fig. S1A). Hence, TM5 may exhibit increased flexibility in the absence of a stable dimer interface, resulting in the lower local resolution observed by cryo-EM.

**Fig. 2.**
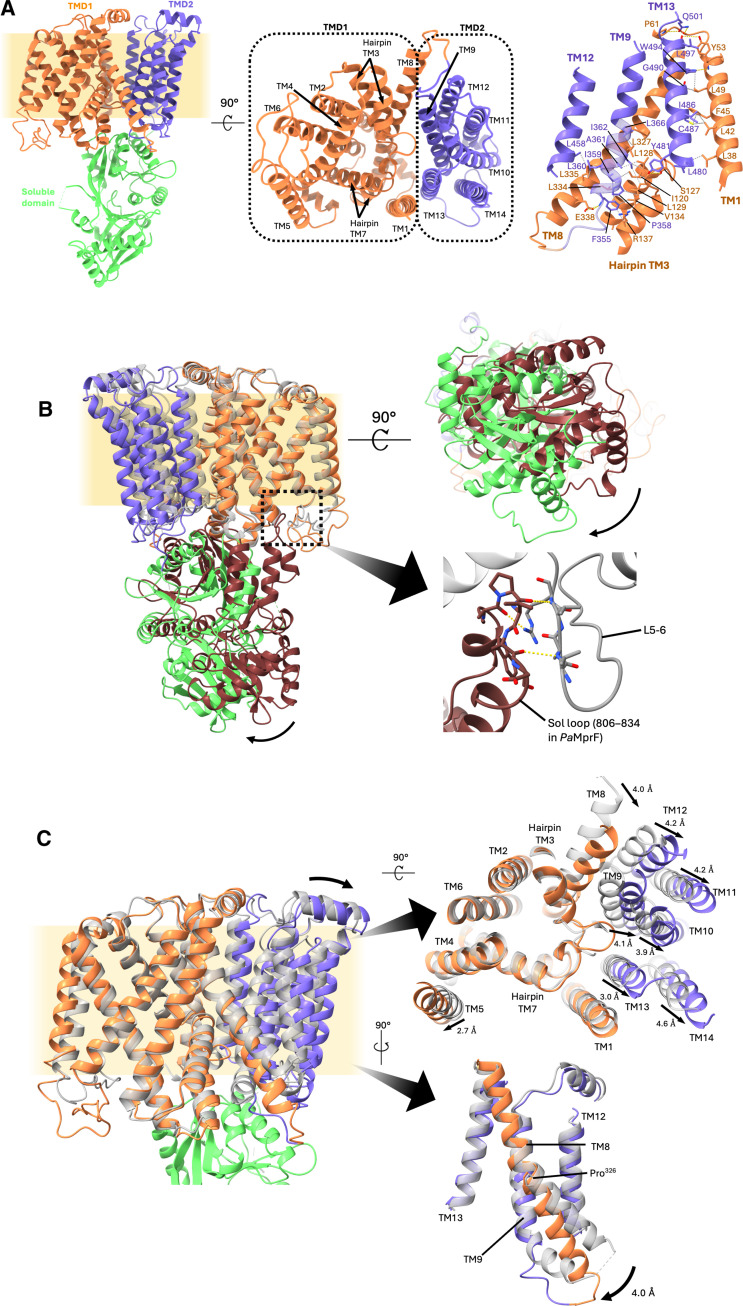
Displacement of MprF domains. (**A**) Side (left) and top view (middle, viewing from the cytoplasmic face clipped through the soluble domain) of *Pa*MprF (sybody removed for clarity). The residues interacting to form the TMD interface are shown (right, polar interactions colored yellow, hydrophobic contacts colored gray). Orange, TMD1; purple, TMD2; green, soluble domain. (**B**) Alignment of *Rt*MprF (PDB: 7DUW; gray, TMDs; magenta, soluble domain) to *Pa*MprF (orange, TMD1; purple, TMD2; green, soluble domain) at TMD1, demonstrating rigid-body rotation of the soluble domain (left). The interaction between the soluble domain and TM L5-6 from *Rt*MprF is shown (bottom right). (**C**) Structures aligned at TMD1 demonstrating displacement of TMD2 (top right, viewed from cytoplasm, soluble domain removed for clarity) and view of TM8 clipped through TMD1 (bottom right). The proline hinge of TM8 (Pro326 in *Pa*MprF) is shown.

The global structural elements of *Pa*MprF are similar to *Rt*MprF (*19*), with the TM region of *Pa*MprF comprising 14 α-helices that can be subdivided into two TM domains (TMDs). TMD1 (TM helices 1 to 8) corresponds to the previously identified flippase domain of MprF (*13*) and contains two pairs of re-entrant hairpin helices (TM3 and TM7), whereas TMD2 (TM helices 9 to 14) connects to the soluble domain (Fig. 2A and figs. S2A and S3). Each individual TMD is structurally very similar to the corresponding domain in *Rt*MprF [root mean square deviation (RMSD) of 1.12 and 0.89 Å for TMD1 and TMD2, respectively]. The interface between the two TMDs is formed by two clusters of mainly hydrophobic interactions: one cluster between TM1 (TMD1) and TM13 (TMD2) and a second cluster between hairpin helix TM3 and TM8 of TMD1 and TM9 and TM12 of TMD2. Very few polar interactions occur at the membrane borders in both clusters. It was previously suggested that MprF-mediated lipid transport could occur via the disruption of a conserved salt bridge between TM7 and TM8, opening a channel between the cytoplasmic and periplasmic lipid-binding sites (*19*). The corresponding salt bridge in *Pa*MprF (E295-R319) is intact (fig. S2B), and there is no substantial intradomain structural reorganization. The soluble domain of the *Pa*MprF full-length structure is very similar to the previously determined crystal structure of the *Pa*MprF soluble domain alone [Protein Data Bank (PDB): 4V35; (*10*)], with an RMSD of 0.63 Å, with the exception of a loop at residues 806 to 834, which could not be resolved in our cryo-EM structure, presumably due to local flexibility. We did not observe any signal for copurified tRNA or lipid molecules at the soluble domain.

### *Pa*MprF adopts an “open” conformation

Despite similar global structural elements, there are substantial rigid-body displacements of domains when comparing *Pa*MprF to *Rt*MprF. The soluble domain is rotated away from the plane of the membrane, adopting a more perpendicular orientation (Fig. 2B, left). This rigid-body movement may be facilitated by the increased flexibility of the aforementioned loop 806 to 834, which in *Rt*MprF forms β strand interactions with a loop between TM5 and TM6 (L5-6) in TMD1 (Fig. 2B, right), “tethering” the soluble domain of *Rt*MprF in proximity to the membrane plane. The untethered, rotated conformation seen in *Pa*MprF may facilitate access to the aminoacyl-tRNA substrate from the cytoplasm (fig. S2C).

In addition to the rotated soluble domain, both TMDs of *Pa*MprF are substantially displaced relative to *Rt*MprF, with TMD1 separating away from both TMD2 and the soluble domain (Fig. 2C, left, top right). The motion appears to originate from TM8, the C-terminal helix of TMD1, which has a hinging point at a proline residue (Pro326 in *Pa*MprF) halfway down the helix at the center of the membrane (Fig. 2C, bottom right). This proline residue is highly conserved across MprF homologs, and previous mutagenesis of the corresponding residue in *S. aureus* MprF (*Sa*MprF) to alanine moderately increased susceptibility to daptomycin (*13*).

**Fig. 3.**
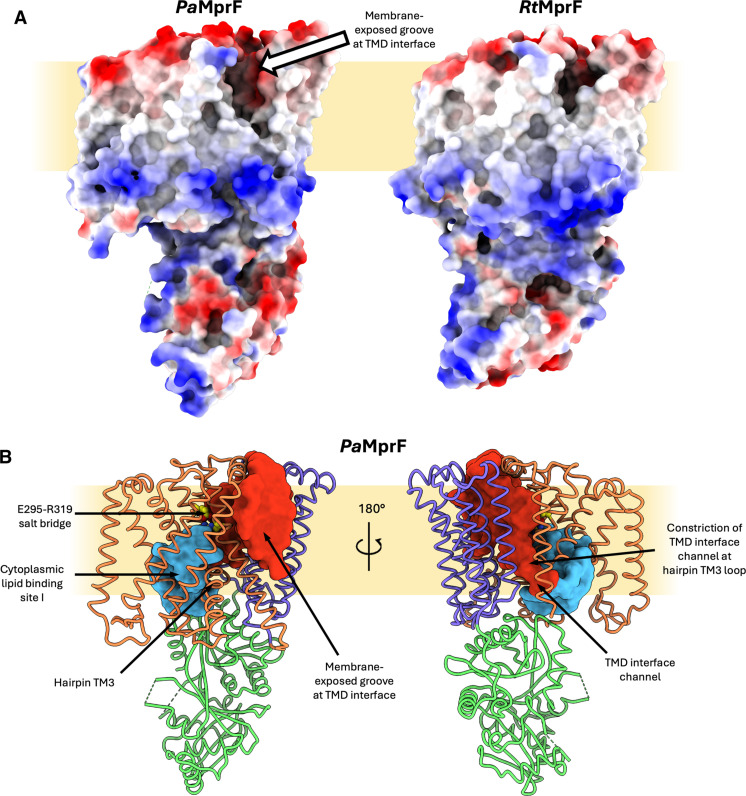
Lipid- and solvent-exposed cavities in *Pa*MprF. (**A**) Comparison of *Pa*MprF (left) and *Rt*MprF (right) electrostatic surfaces. The periplasmic membrane-exposed groove in *Pa*MprF is indicated by an arrow. (**B**) *Pa*MprF (TMD1 = orange, TMD2 = purple, soluble domain = green) ribbon model overlaid with solvent accessible interior surfaces generated by HOLLOW (*70*). The E295-R319 salt bridge residues are highlighted as yellow spheres. A solvent accessible pocket at the cytoplasmic side is shown in blue (lipid binding site I, discussed below), and the channel at the TMD interface, which connects to the membrane-exposed periplasmic groove, is shown in red.

This separation between the two TMDs results in a substantive widening of the membrane-exposed surface, forming a deep groove between the two TMDs that is open to both the periplasm and laterally to the membrane, spanning nearly its entire width (Fig. 3A and red surface in Fig. 3B). The presence of membrane-exposed hydrophilic grooves is common in TMEM16 family lipid scramblases, proteins that facilitate bidirectional lipid transport down a concentration gradient in the absence of an external energy source (*24*–*26*). These grooves are thought to provide a route for the passage of polar lipid head groups via a credit card mechanism (*27*) or to deform the local membrane environment to lower the energy barrier for lipid translocation via a so-called out-of-the-groove mechanism (*28*, *29*). The groove in *Pa*MprF does contain a patch of hydrophilicity at the periplasmic side (fig. S2D), but it does not extend the full width of the membrane as seen in TMEM16 scramblases. If lipid was indeed translocated via this route, then further conformational change would be required to form a continuous surface-mediated lipid path (see also Discussion).

Starting from the wide opening on the periplasmic side, the groove between the two TMDs extends further into the interior of the protein, forming a deep channel that passes diagonally through the protein, along the side of the hairpin helices, reaching all the way through to the cytoplasm (Fig. 3B, red surface). This channel is absent in *Rt*MprF, and its presence does not depend on the disruption of the previously described salt bridge (E295-R319), which is still intact in *Pa*MprF. Because of a constriction near the loop at the end of hairpin TM3, the width of this channel on the cytoplasmic side of *Pa*MprF is too small to accommodate the entry of a lipid molecule. Hence, further structural rearrangements would be necessary at this region to yield a wide enough pathway for lipid transport through the core of the protein. This channel could, however, present a plausible lipid permeation route alternative to the previously suggested pathway (*19*), which starts at a cytosolic lipid binding site (site I, discussed below) adjacent to the constricted end of the channel described above, and is blocked by the conserved salt bridge (Fig. 3B, blue surface). See also Discussion for a comparison of suggested lipid transport routes.

### *Pa*MprF displays extensive lipid binding

**Fig. 4.**
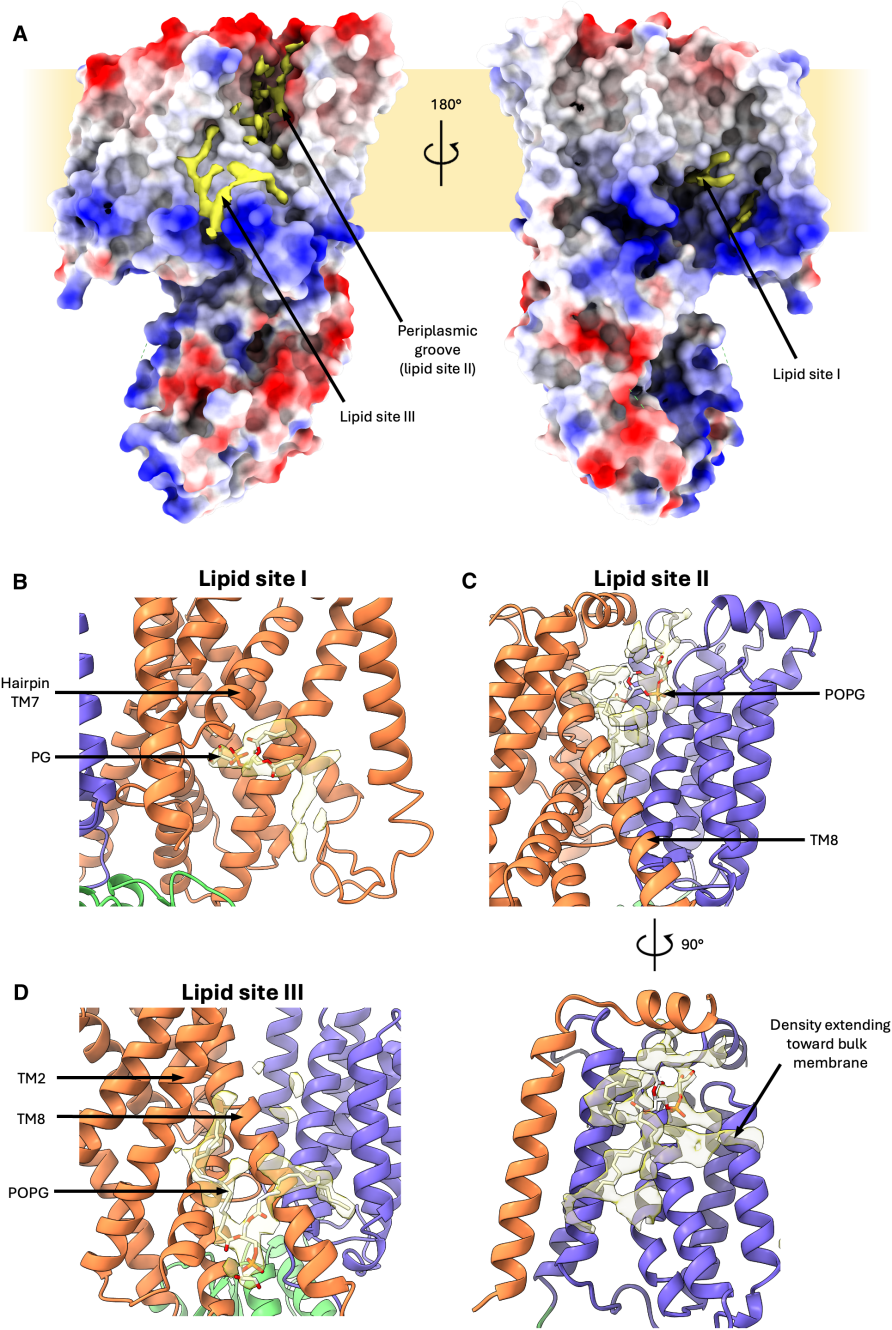
*Pa*MprF lipid-binding sites. (**A**) Electrostatic surface of *Pa*MprF aligned to the lipid features (yellow) present in the final cryo-EM map, contoured at 6 σ. (**B**) View of lipid binding site I (TMD1 = orange, TMD2 = purple, soluble domain = green), with a truncated PG molecule (white sticks) fitted into the map. (**C**) View of lipid signal present in the periplasmic groove between TMDs with a POPG molecule (white sticks) fitted into the map. (**D**) View of lipid binding site III with a POPG molecule fitted into the map.

The *Pa*MprF cryo-EM map displays several features representing bound lipid molecules (Fig. 4A). In the absence of unambiguous evidence for the exact identities of these lipids in our cryo-EM sample, we chose not to include any ligands in our final structural model (PDB: 9GOE). Nevertheless, by tentatively fitting exemplified lipid molecules into the map features, we identified three regions of lipid binding to *Pa*MprF: first, a characteristic Y-shaped phospholipid core is resolved at the same cytoplasm-facing pocket in TMD1 that has been described in *Rt*MprF, formed from TM3, TM4, TM7, and TM8 (Fig. 4B, lipid site I). A tentatively fitted PG head group does not extend as deep into this cavity as the lysyl-PG molecule modeled into the *Rt*MprF structure. This may indicate that unmodified bulk lipid can also occupy this site. Lipid binding at this site has previously been suggested to represent the entry into the proposed salt bridge–gated transport channel through the protein. However, given its orientation facing the expected catalytic face of the soluble domain (*10*), this lipid binding site could alternatively function to facilitate lipid transfer to/from the aminoacylation site.

The second site of lipid binding is at the TMD1/2 interface (formed from TM1, TM3, TM7, and TM8 of TMD1 and TM9, TM12, and TM13 of TMD2) inside the periplasmic groove described above (Fig. 4C, lipid site II). This map region of relatively low local resolution features several continuous sections that would fit one or two lipid molecules, including a map section oriented toward the bulk membrane (highlighted in Fig. 4C). This suggests that this open, membrane-exposed state of *Pa*MprF allows for multiple lipids to occupy this region at the same time.

A previously undescribed, third lipid-binding site is located at the surface of the protein in proximity to the cytoplasmic end of TM8 and allows a reasonable fit of a 1-Palmitoyl-2-oleoyl-*sn*-glycero-3-phosphoglycerol (POPG) molecule (Fig. 4D, lipid site III). As TM8 is adopting an alternate conformation to the corresponding helix in *Rt*MprF through flexion at the Pro326 kink, this lipid-binding site III may act to stabilize the more open conformation of MprF presented here, explaining its absence in the *Rt*MprF structure.

### MD simulations confirm lipid binding to *Pa*MprF and suggest a protein-induced membrane deformation

**Fig. 5.**
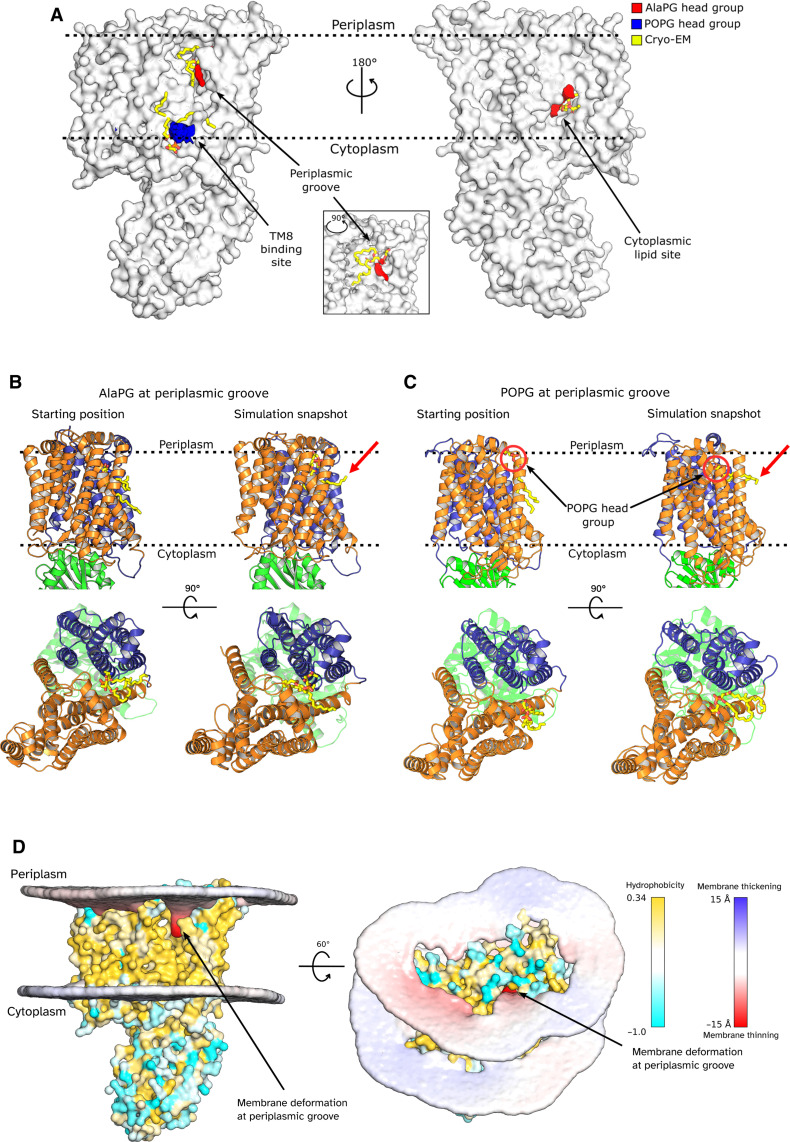
*Pa*MprF MD simulations. (**A**) Lipid head group densities calculated from coarse-grained simulations, superposed with fitted lipid molecules from cryo-EM (Fig. 4). Lipid densities are shown as isosurfaces that represent an above-average occupation of lipids across the frames of the simulation. Densities are only shown in regions of cryo-EM lipid map signal for clarity. (**B**) Atomistic simulation of *Pa*MprF (colored as in Fig. 2) with AlaPG (yellow sticks) at the periplasmic groove. Starting positions (left) and simulation snapshots (right) are shown. (**C**) Same as (B), but with POPG at the periplasmic groove. Protrusion of lipid acyl tails into the bulk membrane is highlighted with red arrows. (**D**) Membrane deformation calculated from coarse-grained simulations. *Pa*MprF is colored by hydrophobicity. The membrane is colored with red for a thinning of the membrane and blue for a thickening of the membrane.

To further investigate the interactions of *Pa*MprF with its native lipid environment, we conducted MD simulations of the structure placed into a symmetrical membrane bilayer composed of 80% POPE, 10% POPG, and 10% AlaPG, reflecting a simplified *P. aeruginosa* inner membrane composition (*30*) but with equal amounts of POPG and AlaPG to avoid introducing any bias when determining binding preferences. Simulations were first run in a coarse-grained (CG) system to allow for long simulation times of 5 μs with five repeats. Lipid density plots confirmed lipid interactions with all sites I, II, and III over the course of the simulations, with a reasonable positional match between the tentatively modeled lipids from the cryo-EM data and the regions of increased lipid head group occupation throughout the simulations (Fig. 5A). The simulation data that show exclusively AlaPG density at both sites suggested to be involved in aminoacylation and transport, namely, the cytoplasmic lipid site (site I) and the periplasmic groove (site II). In contrast, only POPG density is found at the TM8 binding site (site III) suggested to be a “stability” site (Fig. 5A). Further analysis of the trajectories was performed with PyLipID (*31*), which identified binding sites for both AlaPG and POPG at each of the three lipid sites. Binding poses for each of these binding sites were calculated (fig. S4) and then used as starting points for subsequent triplicate atomistic simulations. At site III, the TM8 binding site, AlaPG became detached during two runs while remaining bound only weakly via an acyl tail in the final run (fig. S5A), suggesting that this site is likely not a stable binding site for AlaPG, in line with the suggestion of a stability-conferring lipid site without direct involvement in MprF catalysis. At site I, the cytoplasmic lipid site, POPG remained tightly bound throughout (fig. S5B), indicating that both AlaPG and POPG are able to occupy this site.

At site II, the periplasmic groove, CG simulations identified an AlaPG binding pose oriented parallel to the membrane plane; the head group deeply buried at the TMD interface and the acyl tails oriented outward into the bulk membrane (fig. S4A), in line with the similarly oriented cryo-EM map feature found in this site (Fig. 4C). In one of three atomistic simulations, AlaPG remained tightly bound in this starting position, but in the other two, AlaPG moved even deeper into the groove (Fig. 5B). The POPG binding pose from CG simulations at this site was located at the side of the groove rather than inside (fig. S4A). In one of three atomistic simulations, POPG dissociated from this starting position (fig. S5C), however, during the two remaining runs, POPG moved deeply into the groove, into a similar membrane-parallel orientation with acyl tails oriented out of the groove (Fig. 5C). This indicates that the inside of the groove is able to provide a stable binding site for both AlaPG and POPG, inducing an orientation of the lipid molecules that would be consistent with a credit card model of lipid transport along the groove at the TMD interface of *Pa*MprF.

The extensive dimension and orientation of the membrane-exposed periplasmic groove found in the cryoEM structure led us to speculate whether *Pa*MprF is able to locally deform the bilayer in this region, thereby lowering the energy barrier for lipid translocation via a surface-guided, passively driven mechanism, as in other lipid transporters with membrane-exposed grooves (*29*). The CG simulations show a substantial, and spatially confined thinning of the membrane at the periplasmic groove, reducing the hydrophobic thickness by nearly 50% in this area (Fig. 5D). Together, the MD simulations corroborate the three lipid binding sites identified by cryoEM and support a credit card or out-of-the-groove lipid transport mechanism by *Pa*MprF.

### *Pa*MprF transports fluorescently labeled AlaPG in vitro

**Fig. 6.**
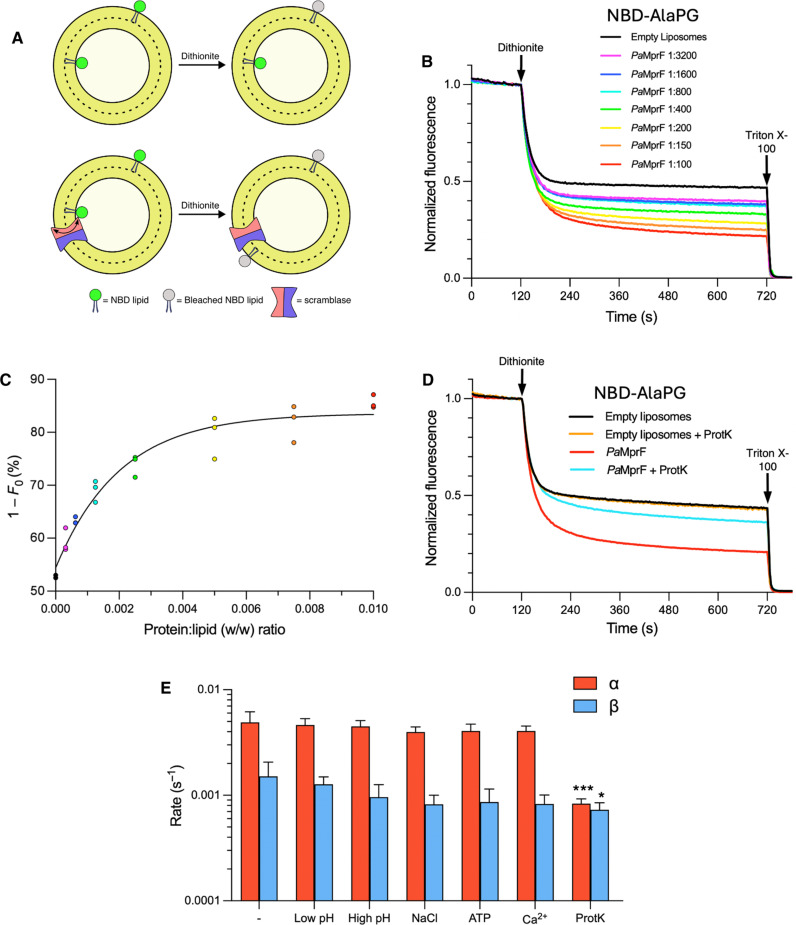
*Pa*MprF lipid transport assay. (**A**) Schematic of liposome-based lipid transport assay, showing empty liposomes in top panels and proteoliposomes reconstituted with a scramblase in bottom panels. (**B**) Lipid transport assay fluorescence time courses for NBD-AlaPG liposomes with different *Pa*MprF:lipid (w/w) ratios. (**C**) Plot of the 1 − *F*_0_ versus *Pa*MprF:lipid (w/w) ratio for each replicate. A mono-exponential fit for the data is shown (*R*^2^ = 0.953), saturating at 1 − *F*_0_ = 83.6%. (**D**) Normalized NBD-AlaPG fluorescence time courses for 1:200 (w/w) *Pa*MprF:lipid liposomes subjected to different treatments after reconstitution. Untreated (red) and proteinase K treated (blue). Empty control liposomes were also measured before (black) and after treatment with proteinase K (orange). (**E**) Bar chart plotting α (red) and β (blue) rate constants of lipid transport mediated by *Pa*MprF in 1:200 (w/w) *Pa*MprF:lipid liposomes in the absence (−) and presence of common activators, as well as following proteinase K (ProtK) treatment. (−) values, *n* = 8; all other values, *n* = 3; error bars represent SD. Asterisks mark values of α and β significantly different from values in the absence of an activator, as determined by unpaired *t* tests (*P ≤ 0.05 and ***P ≤ 0.001).

To investigate the lipid transport capabilities of *Pa*MprF in vitro, the purified protein was reconstituted into liposomes containing a small fraction of nitrobenzoxadiazole (NBD)–labeled lipid. Proteoliposomes were exposed to dithionite, a membrane impermeable compound that bleaches NBD fluorescence (*32*, *33*) thereby reporting on the distribution of fluorescently labeled lipid between the inner and outer leaflets of liposomes (Fig. 6A). Proteoliposomes were produced for a range of *Pa*MprF:lipid ratios using a 16:0/6:0 tail-labeled NBD-AlaPG reporter lipid, mimicking the physiological substrate of *Pa*MprF (Fig. 6B). For all *Pa*MprF:lipid ratios tested, the normalized fluorescence decrease after dithionite addition was greater than for both protein-free liposomes and liposomes reconstituted with a negative control protein [*Ilyobacter tartaricus* adenosine 5′-triphosphate (ATP)–synthase c-ring; fig. S6A], indicating an MprF-dependent transport of NBD-labeled AlaPG between the liposome leaflets. The final fluorescence value, *F*_0_, for each time course before Triton X-100 addition is dependent on *Pa*MprF concentration and saturates at high [*Pa*MprF], as indicated by the mono-exponential fit to 1 − *F*_0_ for each replicate (Fig. 6C). This saturation reflects the fact that at high *Pa*MprF:lipid ratios, the likelihood of all available liposomes containing at least one *Pa*MprF molecule is high, and further increases in [*Pa*MprF] will not affect the final fluorescence value after dithionite addition but merely the rate at which this value is reached (*32*, *34*, *35*). Even at high lipid transporter concentrations, the final fluorescence plateau following dithionite addition will not reach zero, as there is a fraction of liposomes that are multilamellar/refractory to protein insertion (*36*).

Both protein-free liposomes and proteoliposomes were equally insensitive to dithionite-mediated bleaching of incorporated NBD-glucose (fig. S6E) (*34*), ruling out unspecific permeability of proteoliposomes to dithionite. This was further supported by the use of fatty acid–free bovine serum albumin (BSA) as an alternative quenching agent (*33*, *37*), which resulted in a greater fluorescence decrease for proteoliposomes than protein-free liposomes (fig. S6, C and D). Together, these results indicate that *Pa*MprF is exposing inner leaflet NBD-AlaPG to dithionite at the surface of liposomes, providing the first in vitro evidence that *Pa*MprF catalyzes the transport of AlaPG between the leaflets of the bilayer.

As a confirmation that lipid transport is indeed protein dependent, the treatment of *Pa*MprF proteoliposomes with proteinase K after reconstitution resulted in a marked reduction of the observed fluorescence decrease (Fig. 6D), similar to what has been demonstrated in other studies of lipid transport (*37*, *38*). SDS–polyacrylamide gel electrophoresis analysis of proteinase K–treated proteoliposomes before and after solubilization with *n*-Dodecyl-ß-D-maltoside (DDM) confirms a complete digestion of *Pa*MprF in solubilized liposomes (fig. S6G). A small amount of full-length MprF remains undigested in proteoliposomes not presolubilized with DDM. This suggests that *Pa*MprF is less accessible to proteinase K treatment when embedded in intact liposomes and provides an explanation as to why proteinase-treated proteoliposomes still exhibit a small amount of lipid transport activity compared to empty liposomes (Fig. 6D). The protease sensitivity of the observed fluorescence decrease indicates that there is no pre-established asymmetry of NBD-lipid from reconstitution, as this would be unaffected by successive proteolytic treatment. Instead, the observed fluorescence decrease is both dependent on the concentration of *Pa*MprF and sensitive to proteolytic digest, therefore representing the ongoing transport of lipid by *Pa*MprF.

### *Pa*MprF-mediated lipid transport is independent of H^+^- or Na^+^-gradients or common activators

In the experiments described above, lipid transport occurs in the absence of an external energy source, suggesting that *Pa*MprF is passively scrambling NBD-AlaPG in both directions. To quantify whether *Pa*MprF-mediated lipid transport is affected by common activators, as is the case for the TMEM16 family of calcium-activated scramblases (*25*, *39*) and the recently characterized proton-dependent transporter LtaA (*40*), an analytical method to assess lipid scrambling rates developed by Malvezzi *et al.* (*28*) was used to estimate the macroscopic rate constants for forwards (inner leaflet to outer leaflet) and reverse rates of lipid transport (α and β, respectively) within proteoliposomes. This allowed for assessment of whether the addition of common activating compounds to the outside of liposomes would preferentially stimulate lipid transport unidirectionally. Directly before fluorescence measurements, *Pa*MprF proteoliposomes were diluted in buffer containing common activators (1 mM Ca^2+^, 100 mM Na^+^, and 1 mM ATP), or a pH gradient of ±0.4 units across liposome membranes was established. Liposome membrane integrity on the establishment of a pH gradient was confirmed via incorporation of the pH-sensitive dye 8-hydroxypyrene-1,3,6-trisulfonic acid (HPTS; fig. S6F). Triplicate fluorescence time courses for each condition were fitted to a three-state model to estimate α and β (Fig. 6E and fig. S7, A to D). The values of rate constants determined for 1:200 (w/w) *Pa*MprF:lipid liposomes are in the range of 0.004 to 0.005 s^−1^ for α and 0.0008 to 0.002 s^−1^ for β, similar to those reported previously for other scramblases (*25*). Differences between α and β when using this analysis method have also been observed in previous studies (*25*, *28*) and have been suggested to be caused by preferential orientation of protein within liposomes (*28*), although additional factors unaccounted for in the analysis model may affect these values (e.g., liposome surface tension favoring exposure of inner leaflet lipid). There was no observed significant increase in the rates of lipid transport for any of the conditions tested, suggesting that *Pa*MprF lipid scrambling is not affected by these common external activators. In contrast, proteinase K treatment of proteoliposomes reduced the lipid transport rates sixfold for α and twofold for β.

### *Pa*MprF is a highly promiscuous scramblase

**Fig. 7.**
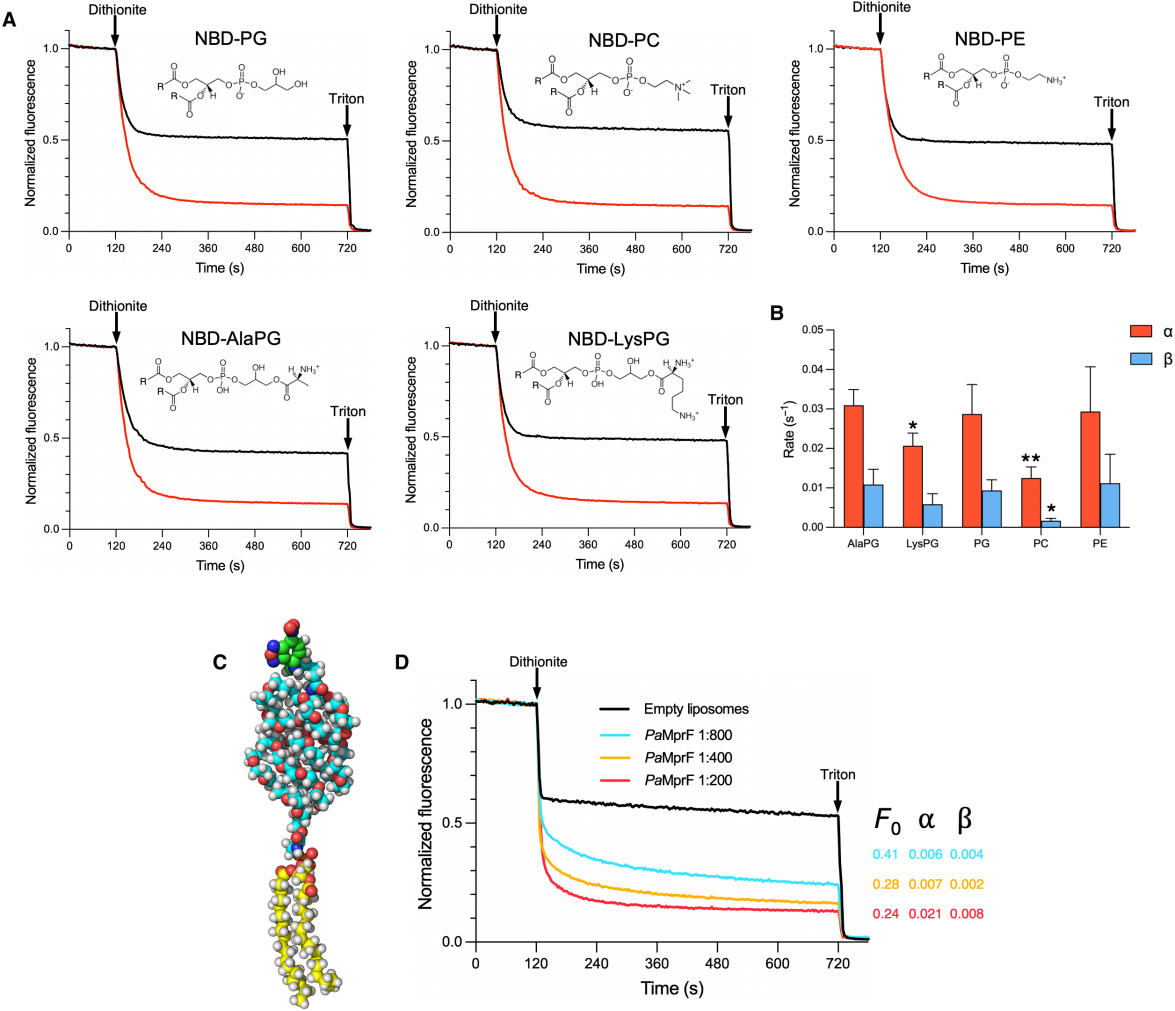
*Pa*MprF-mediated transport of different NBD-lipid species. (**A**) Normalized fluorescence time courses of different NBD-lipids in empty (black) and *Pa*MprF reconstituted (red) liposomes [1:100 (w/w) *Pa*MprF:lipid]. Skeletal formulae of the head groups of the NBD-lipids are shown. (**B**) Bar chart showing the fitted values of α (red) and β (blue) for transport of different lipid species by *Pa*MprF. *n* = 3; error bars represent SD. Asterisks mark values of α and β statistically significant from AlaPG values determined by unpaired *t* tests (*P ≤ 0.05 and **P ≤ 0.01). (**C**) Space-filling model of the DSPE-PEG2000-NBD reporter lipid (colored by element except carbon, which is yellow for DSPE core, cyan for PEG2000 polymer, and green for NBD fluorophore). Previous studies have shown that the PEG2000 polymer adopts a globular conformation with a radius of ~25 Å (*28*). (**D**) Normalized fluorescence time courses assessing DSPE-PEG2000-NBD transport in the established liposome transport assay for control liposomes (black), and *Pa*MprF reconstituted liposomes at different *Pa*MprF:lipid (w/w) ratios (red, orange, and cyan). The calculated values of *F*_0_, α, and β for each trace are given.

A hallmark feature of all well-characterized lipid scramblases is the ability to transport a wide range of lipid substrates between membrane leaflets (*25*, *32*, *35*, *41*). The flippase domain of MprF has been previously demonstrated to exhibit a degree of relaxed specificity; two MprF homologs are present in *Clostridium perfringens*, one of which uses alanine for PG aminoacylation (*Cp*MprF1) and the other lysine (*Cp*MprF2) (*42*). However, *Cp*MprF1 lacks the flippase domain, containing only the C-terminal six TM helices of TMD2, raising the question of whether the flippase domain of *Cp*MprF2 is able to transport both AlaPG and LysPG. An *S. aureus* ΔmprF deletion mutant expressing in trans the AlaPG-producing synthase domain of *Cp*MprF1 with only the flippase domain of either *Cp*MprF2 or *Sa*MprF restored wild-type (WT) levels of daptomycin resistance (*43*), confirming that the substrate specificity at the MprF flippase domain is relaxed. The extent of this relaxed specificity beyond aminoacyl-PG is currently unexplored. To assess the substrate specificity of *Pa*MprF, a variety of commercially available tail-labeled NBD-lipids were tested using the in vitro dithionite assay. NBD-PG and NBD-PC were tested to reflect the bulk lipid composition used for liposome reconstitution, as well as NBD-AlaPG and NBD-LysPG to reflect the substrates of *Pa*MprF and *Rt*MprF, respectively. NBD-PE, representing the main component of the *P. aeruginosa* inner membrane, was also tested (Fig. 7A). For both aminoacylated and unmodified NBD-PG as well as for NBD-PC and NBD-PE, the decrease in fluorescence on dithionite addition was similar, suggesting that *Pa*MprF exhibits broad substrate specificity. The transport of NBD-LysPG provides in vitro evidence that the substrate range of the *Pa*MprF flippase domain includes different aminoacylated-lipid species, as demonstrated in vivo (*43*). In addition, unmodified NBD-PG, NBD-PC, and NBD-PE were also transported, highlighting that aminoacylation of lipids is not a requirement for transport. This high level of promiscuity provides further evidence that *Pa*MprF is functioning as a scramblase in vitro. The rate analysis of these experiments (Fig. 7B) demonstrates that *Pa*MprF has a slight substrate preference for NBD-AlaPG, with α = 0.031 s^−1^ and β = 0.011 s^−1^. NBD-LysPG is transported at a slightly lower rate of α = 0.021 s^−1^. NBD-PC is transported almost threefold slower in the inside-to-outside direction (α = 0.013 s^−1^) than AlaPG and sixfold slower in the outside-to-inside direction (β = 0.0017 s^−1^) than NBD-AlaPG. As PC is a non-native lipid to *P. aeruginosa,* the slower rate of NBD-PC transport is perhaps expected. NBD-PG transport rates are not significantly different to NBD-AlaPG. This suggests that (i) the smaller PG head group is able to access the same lipid transport pathway as aminoacylated lipids, and (ii) the transport process is not sensitive to the different charge distributions on the head groups.

### *Pa*MprF can transport “giant” lipid substrates

The remarkable promiscuity demonstrated by *Pa*MprF raises the question of whether there is a limit on the size of lipid head group that can be transported. This question has been previously addressed for other highly promiscuous scramblases through the use of lipid reporters with giant PEGylated head groups (*28*). To test whether *Pa*MprF is able to transport such a giant lipid, a distearoyl-*sn*-glycero-3-phosphoethanolamine (DSPE) lipid with a polyethylene glycol (PEG) 2000 head group (DSPE-PEG2000-amine) was covalently linked to NBD and purified, producing an NBD reporter lipid with a molecular weight of ~3 kDa, approximately fourfold larger than the NBD-lipids used for the previously described experiments (Fig. 7C and fig. S8). *Pa*MprF is able to transport this PEGylated NBD lipid, with *Pa*MprF proteoliposomes giving a significantly greater fluorescence decrease on dithionite addition than protein-free or c-ring–reconstituted liposomes and with comparable rate constants (Fig. 7D and fig. S6B). As with the smaller lipids, the fluorescence decrease is *Pa*MprF concentration dependent. Given the large size of this substrate, it is highly unlikely that the transport of this PEGylated reporter lipid occurs through the core of *Pa*MprF. Instead, it is most probable that the transport of this giant lipid occurs via an out-of-the-groove mechanism similar to that shown for TMEM16 scramblases.

### *Pa*MprF-mediated lipid transport is highly resistant to mutations at lipid binding sites

**Fig. 8.**
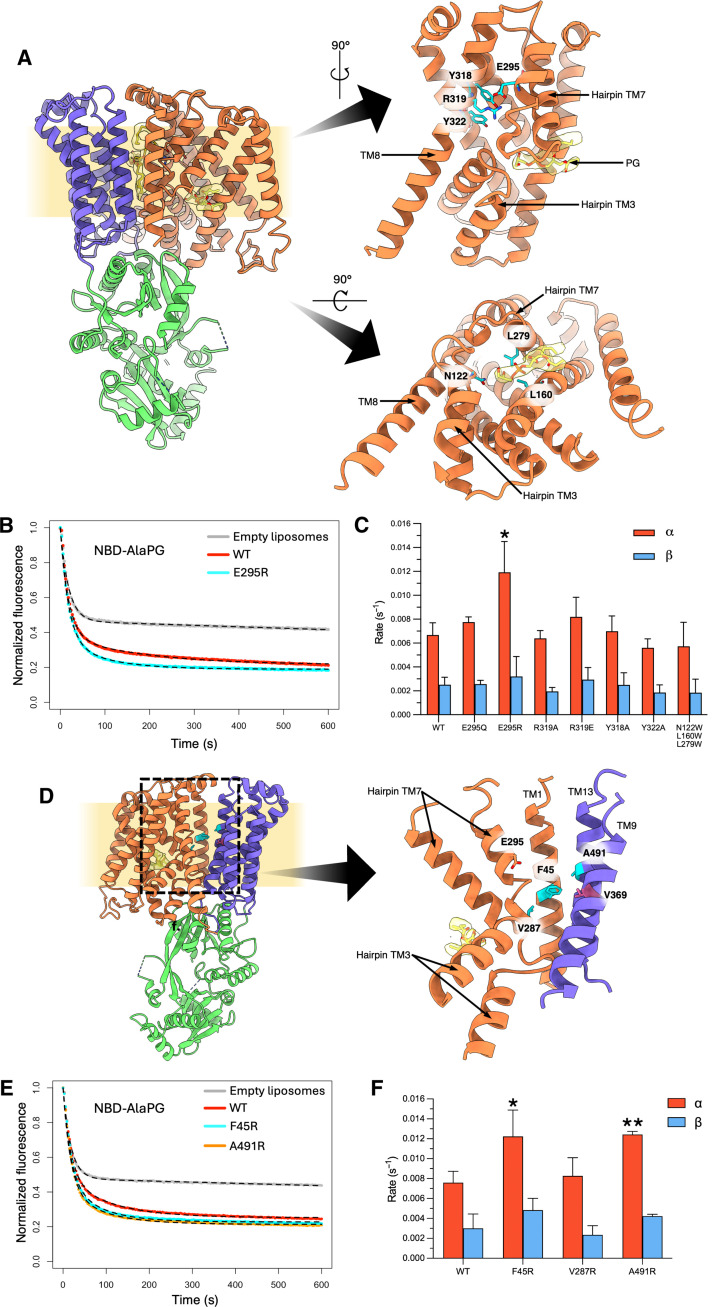
In vitro lipid transport activities of *Pa*MprF mutants. (**A**) Structure of *Pa*MprF, highlighting residues mutated at lipid binding site I (cyan). (**B**) Normalized NBD-AlaPG fluorescence time courses for protein-free (gray), WT (red), and E295R (cyan) proteoliposomes following dithionite addition. The fit to the three-state lipid transport model is shown for each curve as a black dotted line. (**C**) Bar chart of lipid transport rates for lipid binding site I mutants. (**D**) Structure of *Pa*MprF highlighting residues at TMD interface mutated to arginine (cyan) and the residue in *Pa*MprF corresponding to the MRSA gain-of-function mutant M347R (V369, pink). (**E**) Normalized NBD-AlaPG fluorescence time courses for protein-free (gray), WT (red), F45R (cyan), and A491R (orange) proteoliposomes following dithionite addition. The fit to the three-state lipid transport model is shown for each time course as a black dotted line. (**F**) Bar chart of lipid transport rates for TM interface arginine mutants. (For all bar charts; error bars represent SD. n = 3. Asterisks mark values of α and β statistically significant from WT values determined by unpaired *t* tests; *P ≤ 0.05 and **P ≤ 0.01).

To further question whether lipid transport could be occurring via the previously proposed cytoplasmic lipid binding site I (*19*), the key residues at this site, comprising the conserved salt bridge E295-R319 and several conserved tyrosine residues, were probed by mutagenesis. All mutated *Pa*MprF variants behaved similarly to WT *Pa*MprF during purification (figs. S9 and S10, and table S1) and were tested for in vitro NBD-AlaPG transport activity (Fig. 8A, top right). Previous in vivo experiments suggested that mutations in this area stimulated LysPG synthesis/transport in *Rt*MprF (*19*). In *Pa*MprF, mutation of either Y318 or Y322 to alanine and of R319 to alanine or glutamate did not significantly affect NBD-AlaPG transport compared to WT. The protonation-mimicking mutation E295Q also did not result in a stimulation of in vitro lipid transport, further supporting our conclusion that lipid transport is not dependent on the PMF. However, charge reversal of E295 to arginine did result in a significant increase in lipid transport compared to WT, with α increasing almost twofold (Fig. 8, B and C). This is consistent with previous in vivo experiments, where this mutant in *Rt*MprF gave the greatest increase in both total LysPG content and surface-exposed LysPG (*19*). The mutation of these conserved salt bridge residues to alanine in *Sa*MprF, along with mutation of other conserved residues found in TMD1, has been previously shown to significantly increase *S. aureus* sensitivity to daptomycin (*13*); the inspection of the corresponding residues in *Pa*MprF reveals that they mostly contribute to stabilizing interactions within the core of the TMD1 helix bundle (fig. S11); hence, their mutation to alanine likely has a global destabilizing effect on MprF rather than directly affecting lipid interaction and transport. We further probed lipid binding site I by introduction of three tryptophan residues to sterically restrict lipid access to this pocket (Fig. 8A bottom right). However, this triple mutant transported NBD-AlaPG in vitro at similar rates to WT (Fig. 8C), suggesting that lipid transport is not occurring via lipid binding site I.

The TMD interface was also probed by mutagenesis to assess whether this internal channel in *Pa*MprF could be the transport route for AlaPG. Similar to lipid-binding site I, the introduction of tryptophan residues at constriction points along the TMD interface channel did not result in a significant decrease in lipid transport rates compared to WT, again suggesting that lipid transport does not occur via this internal channel (fig. S12). More disruptive mutations were introduced by placing large, highly charged arginine side chains at this interface (Fig. 8D, cyan). Two of these mutations resulted in an increase in transport rates compared to WT; F45R and A491R (Fig. 8, E and F). Structurally, these residues and the previously discussed E295 all occupy a similar region on the periplasmic side of the membrane, suggesting that introduction of a positive charge in this region stimulates lipid transport. Notably, a number of the recently identified gain-of-function MprF mutations in MRSA also introduce positively charged residues at the TMD (*44*). One such mutation, M347R, (corresponding to V369 in *Pa*MprF), is also in proximity to this “periplasmic stimulatory region” (Fig. 8D, pink). All residues mutated in this study are highlighted in an alignment of *Pa*MprF, *Sa*MprF, and *Rt*MprF sequences in fig. S13.

## DISCUSSION

We present here the cryo-EM structure of MprF from the pathogen *P. aeruginosa*, displaying a previously undescribed conformation and three lipid binding sites that were further corroborated by MD simulations. Furthermore, we provide the first in vitro functional evidence that MprF acts as a lipid scramblase.

**Fig. 9.**
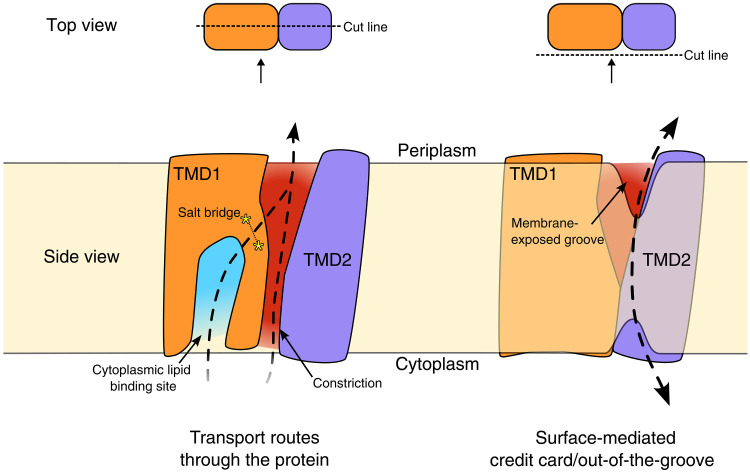
Possible *Pa*MprF lipid transport routes. Schematic of *Pa*MprF (soluble domain removed for clarity) highlighting lipid transport routes assessed in this study. (**Left**) Cut-away view of *Pa*MprF showing possible transport routes through the core of the protein via the cytoplasmic lipid site (blue) or the TMD channel (red). Lipid transport rates of *Pa*MprF were unaffected by obstructive mutations along these routes. (**Right**) Surface view of *Pa*MprF demonstrating a potential credit card/out-of-the-groove transport mechanism at the periplasmic groove, facilitated by membrane deformation. Such a mechanism is consistent with transport of giant lipids.

The structure of *Pa*MprF presented here highlights substantial domain rearrangements in comparison to *Rt*MprF, prompting us to suggest alternate routes of lipid transport to that originally proposed (*19*) (see further discussion and Fig. 9 below). The reason for the conformational differences between *Pa*MprF and *Rt*MprF is unclear. It is unlikely that Sb29 binding to *Pa*MprF has induced any structural rearrangement, as its binding site is far from the observed hinging points. Lipid transport rate constants from *Pa*MprF-Sb29 complex liposomes are not significantly different to *Pa*MprF alone (fig. S7E). One possible reason for the structural differences is the different lipid composition used for SapNP reconstitution; it has been demonstrated that bulk lipid can significantly affect transport properties of membrane proteins (*25*, *45*); hence, the different lipid compositions could alternately stabilize the more closed or open states demonstrated by the two structures. Another key factor is the oligomeric state, as *Rt*MprF is dimeric and *Pa*MprF purifies as a monomer in our hands. Oligomerization-induced structural changes are an appealing hypothesis, as this would also offer a regulatory mechanism of reducing MprF activity in vivo at high MprF concentrations. Such a mechanism could provide an additional level of control over aminoacyl-PG levels in vivo, which have been shown to be detrimental to CAMP resistance of *P. aeruginosa* cells at high concentrations (*14*).

The in vitro transport data presented here show that *Pa*MprF is able to transport a wide variety of lipid species in the absence of an external energy source. This suggests that *Pa*MprF acts as a passive phospholipid scramblase, driving lipid distribution toward an equilibrium between both membrane leaflets. Such a classification is consistent with earlier work in *S. aureus* WT cells, which found that LysPG is evenly distributed between the inner and outer leaflets of the membrane (*7*). Although *Pa*MprF is capable of transporting a range of lipid substrates, rate analysis suggests that both NBD-AlaPG and NBD-PG transport is favored in vitro. Because unmodified PG comprises ~25% of the *P. aeruginosa* membrane (*30*) and AlaPG only ~6% (*3*), this observation raises questions as to whether AlaPG transport is favored in vivo and, if so, how. One possibility could be that the proximity of the aminoacylating soluble domain increases the local concentration of AlaPG near the lipid transport domain, thus increasing the likelihood of transporting AlaPG over unmodified PG. Such a selectivity-by-proximity mechanism is consistent with previous characterization of MprF dimers (*19*) and tetramers (*13*), where the clustering of MprF aminoacylation domains would lead to higher local concentrations of AlaPG in proximity to the TMDs.

These findings would make *Pa*MprF the first dedicated phospholipid scramblase to be characterized in bacteria. Such nonspecific scrambling activity of *Pa*MprF is in fact in line with sequence analyses of the wider MprF family of proteins; it has recently been shown that the MprF lipid transport domain is widespread in bacteria and archaea, often fused to different lipid-modifying soluble enzymes such as O-antigen ligases and glycosyltransferases (*46*). This suggests that the MprF transport domain has evolved as a universal, highly promiscuous transporter of differently modified lipid species, with its respective physiological function depending on the identity of the fused lipid-modifying enzyme, conferring substrate preference by increased local concentration rather than structural selectivity. MprF transport domains have also been identified that are not fused to a soluble enzyme. For example, the gene for YbhN in *E. coli* is located in proximity to cardiolipin synthesis genes in the *E. coli* genome, and YbhN interacts with proteins involved in PG and cardiolipin synthesis (*46*), suggesting a role in membrane homeostasis and transport of unmodified bulk lipid species.

In addition to direct MprF homologs, the eukaryotic DedA family members VMP1 and TMEM41B have recently been demonstrated to be phospholipid scramblases (*35*). In silico structural predictions of this family suggest the presence of the characteristic double inverted hairpin helix motif found in MprF. The identification of fusions of DedA domains to MprF soluble domains in some bacterial species (*47*) further suggests that DedA and MprF lipid transport domains are somewhat interchangeable and involved in the transport of phospholipid species in a broader biological context. Notably, the eukaryotic DedA proteins VMP1 and TMEM41B also do not require an energy source for lipid transport.

The route of lipid transport through MprF remains to be elucidated. Our assessment of possible lipid transport routes by mutagenesis demonstrated that *Pa*MprF tolerates considerable steric obstruction at both the previously suggested route via the cytoplasmic lipid binding site I and also at the channel along the TMD1/2 interface, without any significant reduction in lipid transport rates (Fig. 9, left). This suggests that transport of AlaPG is not occurring through the core of MprF and is not gated by protonation of a salt bridge as previously suggested. This finding raises the question as to the purpose of the cytoplasmic lipid-binding site I, present in both *Pa*MprF and *Rt*MprF, if it is not the entry site for transport. Given the soluble domain approaches the membrane at this binding site in *Rt*MprF, it could be involved in the transfer of PG substrate between the membrane and the soluble domain for aminoacylation. Such a function could explain why an unmodified PG species fits slightly better into the cryo-EM density at this site than an extended AlaPG head group. MD simulations do suggest a preference for AlaPG binding at this site, although PG, when present, also remains bound. Future work should focus on whether mutation at this site impairs the aminoacylation activity of MprF.

Given the wide variety of lipid species transported by *Pa*MprF, particularly its ability to transport giant artificial lipid head groups, it is possible that *Pa*MprF transports lipid via disruption of the membrane through local bilayer deformation at the protein surface, thereby lowering the energy barrier for lipid scrambling (Fig. 9, right). Such a mechanism has been characterized in other scramblases (*28*, *29*). In the case of TMEM16 scramblases, membrane deformation occurs in proximity to membrane-exposed hydrophilic grooves, not unlike the wide membrane-exposed groove formed by TMD separation in the *Pa*MprF structure presented here. In line with this, our MD simulations show a strong local deformation of the outer membrane leaflet at the periplasmic groove. However, further conformational change at the TMD interface may be required to extend the groove to the full width of the membrane. The inspection of the cryo-EM map region corresponding to the SapNP indicates a slight deformation of the membrane in proximity to the TMD interface (fig. S14), albeit to a lesser magnitude than observed in MD simulations. This disparity may be due to the relatively small amount of lipid included in the SapNP, leading to a more compact packing of the NPs as compared to more conventional membrane mimetics with larger lipid:protein ratios (*22*). Future experimental assessment of the ability of MprF to deform the bilayer in larger membrane mimetic systems will help to clarify this issue. No individual lipid scrambling events were observed over the course of the MD simulations performed in this study due to the elastic network used in the CG simulations preventing any conformational changes in the protein.

Reconciling the effect of “daptomycin hypersensitizing” mutations described earlier for *Sa*MprF (*13*) is complex: An actual stimulation of aminoacyl-PG synthesis by mutation may be masked in vivo by lowered expression levels, as observed for some *Rt*MprF mutants (*19*), and both a decrease and a strong enrichment of aminoacyl-PG in the bacterial membrane may cause increased sensitivity (*14*). Nevertheless, the observed stimulation of lipid transport by introduction of a positive charge within the membrane exposed groove of *Pa*MprF is particularly interesting, as it concurs with MRSA gain-of-function mutations in a similar location, such as M347R. M347R in MRSA increases surface-positive charge of bacteria (*44*), further supporting the suggestion that positively charged residues at this region stimulate lipid transport. The mechanism for this stimulation is unclear; given that these residues line the wide, membrane-exposed periplasmic exit cavity, one might speculate that the introduction of large and charged side chains could facilitate the separation of TMDs, further widening the membrane-exposed groove. Such a rationalization would be in line with a lipid transport route via this surface groove, consistent with the indications from membrane deformation in MD simulations, the tryptophan mutagenesis of alternate pathways, and the ability of *Pa*MprF to transport giant PEGylated lipids. A future in-depth characterization of the molecular mechanism underlying this stimulation could aid in targeting MprF in daptomycin-resistant *S. aureus* strains.

## MATERIALS AND METHODS

### *Pa*MprF cloning, expression, and purification

The *Pa*MprF gene was cloned from PAO1 genomic DNA into the expression vector p7XC3H (*48*) and expressed in *E. coli* C41 with a C-terminal 10xHis tag. Cells were grown to an optical density at 600 nm (OD_600_) of 0.6 before induction with 0.5 mM isopropyl-β-d-thiogalactopyranoside (IPTG) overnight at 20°C. Cells were harvested and resuspended in 20 mM Hepes (pH 7.6), 200 mM KCl, and 10% glycerol and lysed at 30 kpsi with two passes through a cell disruptor (Constant Systems). Cell debris was removed by centrifugation at 17,000*g* for 1 hour at 4°C, and then membranes were isolated by ultracentrifugation at 100,000*g* for 1 hour at 4°C. Membranes were solubilized in 1.2% (w/v) DDM for 1 hour at 4°C and then incubated with Ni Sepharose 6 FF resin (Cytiva) for 1 hour at 4°C. Ni-nitrilotriacetic acid resin was washed with 100 bed volumes of buffer containing 50 mM imidazole and then eluted with buffer containing 250 mM imidazole. Eluate was subjected to SEC with an S200 increase column (Cytiva). The monomeric *Pa*MprF fraction was pooled and concentrated for downstream experiments. *Pa*MprF mutants were generated using a Quikchange II site-directed mutagenesis kit (Agilent) and expressed and purified as for WT *Pa*MprF. A list of all primer sequences used to generate WT and mutant *Pa*MprF constructs in this study is given in table S2.

### SapA purification

The sequence for human SapA was expressed in a pNIC-Bsa4 vector (provided by J. Frauenfeld, Salipro Biotech AB, Sweden). The expression and purification of SapA were performed as described previously (*21*). Briefly, transformed Rosetta pLysS *E. coli* were grown to an OD_600_ of 1.2 before induction with 0.7 mM IPTG overnight at 25°C. Cells were harvested and resuspended in 20 mM Hepes (pH 7.5), 150 mM NaCl, and 20 mM imidazole and lysed at 30 kpsi with two passes through a cell disruptor (Constant Systems). Lysate was incubated at 85°C in a water bath for 10 min and centrifuged at 40,000*g* for 1 hour to pellet precipitated proteins. The supernatant containing SapA was collected and incubated with Ni Sepharose 6 Fast Flow resin (Cytiva) for 1 hour. Affinity resin was washed with 30 bed volumes buffer containing 40 mM imidazole to remove nonspecifically bound proteins before elution with 5 bed volumes of buffer containing 400 mM imidazole. Eluate was supplemented with 1 mg of His-tagged TEV protease per 12.5 mg of SapA and dialyzed overnight at room temperature (RT). Dialysate was passed over re-equilibrated affinity resin and the flow-through collected. Flow-through was concentrated in 3 kDa molecular weight cut-off Vivaspin 20 concentrators (Sartorius) and subjected to SEC with an S200 PG column (Cytiva). The peak fraction was collected and used for reconstitution with *Pa*MprF.

### Sybody selection

Sybody selections were performed as described by Zimmerman *et al.* (*20*, *49*) against full-length biotinylated *Pa*MprF. The *Pa*MprF gene was cloned into the expression vector pBXCA3H (*48*) to introduce a C-terminal AviTag followed by a 3C cleavage site and 10x His tag; Avi-tagged *Pa*MprF was expressed and purified as described above [with the exception of cells being induced with 0.02% (w/v) l-arabinose] and biotinylated with recombinantly purified BirA. All sybody selection steps were performed at 4°C in buffers supplemented with 0.026% (w/v) DDM. *Pa*MprF-specific binders were enriched by one round of ribosome display followed by two rounds of phage display. Individual *Pa*MprF-specific binders were identified by enzyme-linked immunosorbent assay (ELISA) using full-length *Pa*MprF. A selection of hits displaying a range of ELISA signals were sequenced, expressed, and purified, and duplicated sequences/sybodies with poor SEC elution profiles were discarded. A total of 31 unique sybodies were obtained (for further selection details, see table S3). The TM region-specific binder Sb29 (from the loop sybody sublibrary) was identified by pull-down assay with a truncated *Pa*MprF construct of residues 1 to 545 and used for cryo-EM sample preparation.

### Cryo-EM sample preparation and data collection

Purified *Pa*MprF, SapA, and POPC:POPG (7:3 mol/mol), were mixed at a 1:25:40 molar ratio on ice for 1 hour. Detergent was removed by incubation with 50 mg of activated Biobeads-SM2 (Bio-Rad) for 1 hour at 4°C, followed by incubation overnight at 4°C with a further 100 mg of Biobeads. *Pa*MprF-loaded SapNPs were separated from empty SapNPs by SEC and mixed with a 1.2× molar excess of Sb29 before application to cryo-EM grids. Grids were prepared by applying 3 μl of *Pa*MprF-Sb29 SapNPs at 0.8 mg/ml to glow-discharged holey carbon grids (Quantifoil R 1.2/1.3 Cu, 300 mesh). Grids were blotted at 100% humidity and 4°C for 6 s before being plunge frozen in liquid ethane with a Vitrobot Mark IV (FEI).

*Pa*MprF-Sb29 SapNP grids were imaged using a Titan Krios Cryo-TEM (Thermo Fisher Scientific) at the Central Oxford Structural Molecular Imaging Centre (COSMIC), Oxford, operated at 300 kV with a Gatan BioQuantum energy filter (20 eV) and a Gatan K3 direct electron detector. Movies were collected at a nominal ×105,000 magnification at 0.83 Å/pixel with an 11.4 e^−^/pixel/second dose rate for a 2.6-s exposure time, resulting in a total dose of ~42 e^−^/Å^2^. Movies were collected using a 100-μm objective lens and a defocus range of −2.25 to −0.75 μm in 0.25-μm steps.

### Cryo-EM data processing

Datasets were processed in CryoSPARC (*50*). Briefly, following motion correction and contrast transfer function (CTF) estimation of collected movies, particles were picked using blob picker and 2D classified to produce initial templates that were used for template picking. Template picked particles were then subjected to multiple rounds of two-dimensional (2D) classification before the best particle sets were used for ab initio 3D reconstruction. These particles were then taken forward to heterogeneous refinement using the ab initio model low-pass filtered to 12 Å as three starting classes. The particle sets from the best two classes were subjected to a round of 3D classification (using three starting ab initio models low-pass filtered to 12 Å and a focus mask with a 15-pixel soft edge that excluded the SapNP micelle). The particle set comprising the class with the best defined sybody map was taken forward to local CTF refinement and used for a final round of nonuniform refinement. This output was sharpened with a *B* factor of −85 Å^2^, producing the post-processed map used for model building.

### Model building and refinement

The *Pa*MprF-Sybody29 model was built in Coot (*51*) using homology models of *Pa*MprF and Sybody29 as initial templates. Homology models were generated from one chain of the *Rt*MprF cryo-EM structure [PDB: 7DUW; (*19*)] and a sybody binder [PDB: 3P0G; (*52*)] using SWISS-MODEL (*53*). Model refinement was performed in PHENIX (*54*) with iterative rounds of real-space refinement with rotamer and secondary structure restraints using the final map at 3.28-Å resolution. Statistics for data collection, reconstruction, and model building are reported in table S4).

### Molecular simulation setup

All simulations were run using GROMACS 2022 (*55*). MprF was positioned in a membrane using the Martini 3 CG force field and solvated with water and 0.15 M NaCl to neutralize the system (*56*). The membranes were constructed using insane with a 8:1:1 ratio of PE:PG:AlaPG lipids (*57*). An elastic network of 500 kJ mol^−1^ nm^−2^ was applied between all backbone beads between 0.5 and 0.9 nm. Electrostatics were described using the reaction field method, with a cutoff of 1.1 nm using the potential shift modifier, and the van der Waals interactions were shifted between 0.9 and 1.1 nm. The systems were first energy minimized by steepest descent algorithm to 1000 kJ mol^−1^ nm^−1^ and then simulated for a total of 5 runs of 5 μs. The temperature and pressure were kept constant throughout the simulation at 310 K and 1 bar, respectively, with protein, lipids, and water/ions coupled individually to a temperature bath by the V-rescale method (*58*) and a semi-isotropic c-rescale (*59*). The final snapshots from the CG simulations were then converted back to an atomistic description using CG2AT2 (*60*). Lipid density plots were generated by calculating for each point in space the number of frames in which a specific lipid was present at that point, displayed as isosurfaces. Binding sites for PG and AlaPG were identified using PyLipID (*31*), and atomistic simulations were set up from bound poses using LipIDens (*61*).

### Atomistic simulations

Simulations of the MprF structure were performed without position restraints for a total of 100 ns and run in triplicate. In all cases, a 2-fs time step was used in an NPT ensemble with V-rescale temperature coupling at 310 K (*58*) and a semi-isotropic Parrinello-Rahman barostat at 1 bar, with protein, water/ions, and, if included, lipids coupled individually (*62*). Electrostatics was described using the Particle-mesh Ewald method, with a cutoff of 1.2 nm, and the van der Waals interactions were shifted between 1 and 1.2 nm. The TIP3P water model was used, the water bond angles and distances were constrained by SETTLE (*63*). H bonds were constrained using the LINCS algorithm (*64*). Analysis was performed using MDAnalysis (*65*) and visualized in PyMOL (*66*).

### Proteoliposome reconstitution

Chloroform-solubilized lipid stocks of POPC and POPG were mixed in a 7:3 (mol:mol) ratio along with 0.4 mol % NBD-labeled reporter lipid. The lipid mixture was dried in a round bottom flask with a nitrogen stream to form a lipid film. Residual solvent was removed by drying overnight in a vacuum desiccator. The lipid film was rehydrated to a total lipid concentration of 20 mg/ml with liposome reconstitution buffer [100 mM Hepes (pH 7.6) and 100 mM KCl] for 2 hours at RT protected from light to limit NBD photobleaching. Rehydrated lipid was extruded through a 400-nm membrane filter (Merck Millipore) using a mini-extruder (Avanti) 31 times to form liposomes. Liposomes were incubated with 0.4% (w/v) DDM for 3 hours at RT. *Pa*MprF at the desired *Pa*MprF:lipid (w/w) ratio was then added to the destabilized liposomes and incubated at 4°C for 30 min. DDM was removed by the addition of Biobeads-SM2 (Bio-Rad); 80 mg of Biobeads per ml liposomes were added and incubated at 4°C for 2 hours. This was repeated with a fresh batch of Biobeads (80 mg/ml), and then Biobeads (80 mg/ml) were added to samples for incubation overnight at 4°C. The next morning, Biobead incubation (80 mg/ml) was performed to ensure complete DDM removal, and then Biobeads were removed, and liposomes were either measured immediately in a fluorescence-based lipid transport assay described below or frozen in liquid nitrogen and stored at −80°C for future use. When using stored liposomes, samples were thawed and subjected to extrusion as described above to ensure that liposomes were unilamellar for fluorescence measurements. Variations in the recovery of lipid in the initial rehydration of lipid films meant that lipid transport measurements could not be directly compared across different batches of liposomes (*28*, *67*), and so internal controls were included in all reconstitutions. For experiments using *I. tartaricus* c-ring as a negative control, c-ring was provided by T. Meier, Imperial College, London, UK and was reconstituted into liposomes at a 1:100 (w/w) c-ring:lipid ratio.

### DSPE-PEG2000-NBD lipid synthesis

DSPE-PEG2000-NBD was synthesized as described by Malvezzi *et al.* (*28*). Briefly, 9.8 mg of DSPE-PEG2000-amine (Avanti) and 2.7 mg of succinimidyl 6-(*N*-(7-nitrobenz-2-oxa-1,3-diazol-4-yl)amino)hexanoate (NBD-SE, Cambridge Bioscience) were dissolved in 1.5 ml of dichloromethane. 10 μl of triethylamine were added, and the reaction mix was stirred at RT in the dark for 2 hours. The reaction mixture was separated by TLC on silica gel 60 plates (Merck Millipore) with a mobile phase of 85:15 chloroform:methanol (v/v). The DSPE-PEG2000-NBD band was scraped from the plate, and the lipid was isolated by incubation with 12 volumes of methanol, followed by filtration to remove silica. The purified DSPE-PEG2000-NBD was analyzed by mass spectrometry to confirm its identity (fig. S8).

### In vitro lipid transport assay

NBD fluorescence of liposomes was monitored as a time course in a 2-ml quartz cuvette using a LS55 fluorescence spectrometer (PerkinElmer). Measurements were taken at 25°C every 3 s at excitation = 460 nm, emission = 520 nm with 10-nm excitation and emission slit widths, and a 0.5 s integration time. A 40 μl of reconstituted liposomes were diluted in 2 ml of liposome buffer [100 mM Hepes (pH 7.6) and 100 mM KCl], and fluorescence was monitored for an initial 2 min to allow for sample equilibration with constant stirring. After 2 min, 20 μl of 2 M sodium dithionite (dissolved in 1 M tris) was added to the sample cuvette (20 mM final concentration), and the evolution of fluorescence over time was measured for 10 min. After 10 min, 20 μl of 10% (v/v) Triton X-100 was added [0.1% (v/v) final concentration] to solubilize liposomes and expose any remaining protected NBD to dithionite. For proteinase K experiments, proteoliposomes were treated with proteinase K (0.17 mg/ml) at 37°C for 30 min before fluorescence measurement.

For BSA back extraction assay measurements, the assay was repeated as above, except that fatty acid–free BSA (2 mg/ml; Sigma-Aldrich) was added at *t* = 120 s instead of 20 mM dithionite. For NBD-glucose permeability control experiments and HPTS proton permeability assays, liposomes were made as described above, but with NBD-labeled lipid removed from the bulk lipid and replaced with either 30 μM NBD-glucose or 40 mM HPTS. Following proteoliposome formation, liposomes were ultracentrifuged at 100,000*g* for 2 hours, and the supernatant was removed. Pelleted liposomes were washed four times with 100 mM Hepes (pH 7.6) and 100 mM KCl, and the supernatant was tested for fluorescence to confirm the removal of any NBD fluorophore not incorporated into liposomes. Liposomes were then resuspended in 1 ml of the same buffer for fluorescence measurements.

### Quantification of lipid transport rates

Lipid transport rates were estimated using the model derived by Malvezzi *et al.* (*28*), in which the populations of NBD-labeled lipid within proteoliposomes can be described by the following three-state model

$$L_{i}\underset{\beta }{\overset{\alpha }{⇌}}L_{o}\overset{\gamma }{\to }L_{*}$$ (1)

where $L_{i}$ is the population of NBD fluorescent lipid in the inner leaflet of the liposome, $L_{o}$ is the population in the outer leaflet, and $L_{*}$ is the population of NBD lipid bleached by dithionite. Interconversion between $L_{i}$ and $L_{o}$ is mediated by *Pa*MprF, with α and β representing the forwards and reverse rate constants for lipid transport, respectively. γ is the rate constant for bleaching of $L_{o}$ to $L_{*}$ and is irreversible. As there is a fraction of liposomes that are refractory to protein insertion, the total fluorescence of the system at any time, $F_{\text{tot}}\left(t\right)$, is given as

$$F_{\text{tot}}\left(t\right)=f_{0}F_{\text{PF}}\left(t\right)+\left(1-f_{0}\right)F_{\text{Scr}}\left(t\right)$$ (2)

where $F_{\text{PF}}\left(t\right)$ is the fluorescence of protein-free liposomes, $F_{\text{Scr}}\left(t\right)$ is the fluorescence of liposomes containing at least one active scramblase molecule, and $f_{0}$ is the fraction of liposomes that are protein free. $F_{\text{PF}}\left(t\right)$ can be written in terms of γ as

$$F_{\text{PF}}\left(t\right)=L_{i}^{\text{PF}}+\left(1-L_{i}^{\text{PF}}\right)e^{-\gamma t}$$ (3)

where $L_{i}^{\text{PF}}$ is the fraction of NBD lipid in the inner leaflet of protein-free liposomes. Similarly, $F_{\text{Scr}}\left(t\right)$ can be written in terms of α, β, and γ, resulting in the full equation of $F_{\text{tot}}\left(t\right)$

$$\begin{array}{c} F_{\text{tot}}\left(t\right)=f_{0}\left[L_{i}^{\text{PF}}+\left(1-L_{i}^{\text{PF}}\right)e^{-\gamma t}\right]\\ +\frac{\left(1-f_{0}\right)\left[\alpha \left(\lambda_{2}+\gamma \right)\left(\lambda_{1}+\alpha +\beta \right)e^{\lambda_{1}t}+\lambda_{1}\beta \left(\lambda_{2}+\alpha +\beta +\gamma \right)e^{\lambda_{2}t}\right]}{D\left(\alpha +\beta \right)} \end{array}$$ (4)

where

$$\begin{array}{c} \lambda_{1}=\frac{\left(\alpha +\beta +\gamma \right)-\sqrt{{\left(\alpha +\beta +\gamma \right)}^{2}-4\alpha \gamma }}{2},\\ \lambda_{2}=\frac{\left(\alpha +\beta +\gamma \right)+\sqrt{{\left(\alpha +\beta +\gamma \right)}^{2}-4\alpha \gamma }}{2} \end{array}$$

and

$$D=\left(\lambda_{1}+\alpha \right)\left(\lambda_{2}+\beta +\gamma \right)-\alpha \beta$$

Experimental data were fitted to the model above in R using the Nonlinear least squares function. Protein-free liposomes were included in all reconstitution experiments and traces from protein-free liposomes fitted to Eq. 3 to obtain estimates of γ and $L_{i}^{\text{PF}}$. Proteoliposome data were then fitted to Eq. 4 using these values of γ and $L_{i}^{\text{PF}}$ to determine values of α and β. Values for γ and $L_{i}^{\text{PF}}$ vary based on NBD-lipid identity (*68*), so in experiments assessing substrate specificity, protein-free liposomes were produced for each NBD-lipid to account for these differences. Fluorescence decay results are most comparable within a reconstitution experiment due to variations in lipid recovery during liposome reconstitution (*67*). Hence, for all experiments estimating rate constants, three independent liposomes reconstitutions were performed using the same batch of prepared bulk lipid to account for variation in reconstitution efficiency. When assessing substrate specificity, the *Pa*MprF:lipid (w/w) ratio was increased to 1:100 to give larger rate constants for lipid transport such that differences in rates could be more easily quantified.

In all liposomes, a slow linear decay in fluorescence can be observed that reflects the combined processes of NBD photobleaching and slow entry of dithionite into liposomes. The rate constant of this decay was determined in all reconstitutions to be of the order ~10^−5^ s^−1^, similar to other values reported in the literature (*28*, *69*). As this decay is several orders of magnitude smaller than the determined lipid transport rate constants, it was not included in proteoliposome analysis as it had a negligible effect on the determined rate constants from fitting.

Variations in f_0_ affect the determined values of α and β. As the same concentration of WT and mutant *Pa*MprF was used in experiments investigating mutagenic effects on transport, the values of f_0_, α and β were determined for the most catalytically active construct (proteoliposomes that gave the greatest decrease in fluorescence on dithionite addition), and then this estimated value of f_0_ was fixed for other proteoliposomes within the same reconstitution experiment.

### Statistical analysis

For analysis of lipid transport rates, independent liposome reconstitutions were performed (*n* ≥ 3). Rate constants were estimated as described above and displayed as the mean with error bars representing SD. For assessment of lipid specificity, unpaired *t* tests were performed for each lipid tested compared to NBD-AlaPG (table S5). For investigation of common activators, unpaired *t* tests were performed for each condition compared to liposomes in the absence of an activator (table S6). For investigation of mutant *Pa*MprF activity, unpaired *t* tests were performed for each mutant compared to WT *Pa*MprF (table S7).

## Supplementary Materials

### This PDF file includes:

Figs. S1 to S14 Tables S1 to S7 References

## References

[1] A. Peschel, R. W. Jack, M. Otto, L. V. Collins, P. Staubitz, G. Nicholson, H. Kalbacher, W. F. Nieuwenhuizen, G. Jung, A. Tarkowski, K. P. M. van Kessel, J. A. G. van Strijp, *Staphylococcus aureus* resistance to human defensins and evasion of neutrophil killing via the novel virulence factor MprF is based on modification of membrane lipids with l-lysine. J. Exp. Med. 193, 1067–1076 (2001). 11342591 10.1084/jem.193.9.1067 PMC2193429

[2] P. Vinuesa, F. Neumann-Silkow, C. Pacios-Bras, H. P. Spaink, E. Martínez-Romero, D. Werner, Genetic analysis of a pH-regulated operon from *Rhizobium tropici* CIAT899 involved in acid tolerance and nodulation competitiveness. Mol. Plant Microbe Interact. 16, 159–168 (2003). 12575750 10.1094/MPMI.2003.16.2.159

[3] S. Klein, C. Lorenzo, S. Hoffmann, J. M. Walther, S. Storbeck, T. Piekarski, B. J. Tindall, V. Wray, M. Nimtz, J. Moser, Adaptation of *Pseudomonas aeruginosa* to various conditions includes tRNA-dependent formation of alanyl-phosphatidylglycerol. Mol. Microbiol. 71, 551–565 (2009). 19087229 10.1111/j.1365-2958.2008.06562.x

[4] P. W. Simcock, M. Bublitz, F. Cipcigan, M. G. Ryadnov, J. Crain, P. J. Stansfeld, M. S. P. Sansom, Membrane binding of antimicrobial peptides is modulated by lipid charge modification. J. Chem. Theory Comput. 17, 1218–1228 (2021). 33395285 10.1021/acs.jctc.0c01025

[5] M. G. Macfarlane, Characterization of lipoamino-acids as O-amino-acid esters of phosphatidyl-glycerol. Nature. 196, 136–138 (1962).

[6] H. Roy, Tuning the properties of the bacterial membrane with aminoacylated phosphatidylglycerol. IUBMB Life 61, 940–953 (2009). 19787708 10.1002/iub.240 PMC2757517

[7] C. M. Ernst, P. Staubitz, N. N. Mishra, S.-J. Yang, G. Hornig, H. Kalbacher, A. S. Bayer, D. Kraus, A. Peschel, The bacterial defensin resistance protein MprF consists of separable domains for lipid lysinylation and antimicrobial peptide repulsion. PLOS Pathog. 5, e1000660 (2009). 19915718 10.1371/journal.ppat.1000660 PMC2774229

[8] W. J. Lennarz, J. A. Nesbitt III, J. Reiss, The participation of sRNA in the enzymatic synthesis of OL-lysyl phosphatidylgylcerol in *Staphylococcus aureus*. Proc. Natl. Acad. Sci. U.S.A. 55, 934–941 (1966). 5219701 10.1073/pnas.55.4.934 PMC224253

[9] S. Hebecker, W. Arendt, I. U. Heinemann, J. H. Tiefenau, M. Nimtz, M. Rohde, D. Söll, J. Moser, Alanyl-phosphatidylglycerol synthase: mechanism of substrate recognition during tRNA-dependent lipid modification in *Pseudomonas aeruginosa*. Mol. Microbiol. 80, 935–950 (2011). 21392131 10.1111/j.1365-2958.2011.07621.x PMC3111014

[10] S. Hebecker, J. Krausze, T. Hasenkampf, J. Schneider, M. Groenewold, J. Reichelt, D. Jahn, D. W. Heinz, J. Moser, Structures of two bacterial resistance factors mediating tRNA-dependent aminoacylation of phosphatidylglycerol with lysine or alanine. Proc. Natl. Acad. Sci. U.S.A. 112, 10691–10696 (2015). 26261323 10.1073/pnas.1511167112 PMC4553816

[11] J. A. Nesbitt III, W. J. Lennarz, Participation of aminoacyl transfer ribonucleic acid in aminoacyl phosphatidylglycerol synthesis. I. Specificity of lysyl phosphatidylglycerol synthetase. J. Biol. Chem. 243, 3088–3095 (1968). 5653192

[12] C. M. Ernst, A. Peschel, Broad-spectrum antimicrobial peptide resistance by MprF-mediated aminoacylation and flipping of phospholipids. Mol. Microbiol. 80, 290–299 (2011). 21306448 10.1111/j.1365-2958.2011.07576.x

[13] C. M. Ernst, S. Kuhn, C. J. Slavetinsky, B. Krismer, S. Heilbronner, C. Gekeler, D. Kraus, S. Wagner, A. Peschel, The lipid-modifying multiple peptide resistance factor is an oligomer consisting of distinct interacting synthase and flippase subunits. mBio 6, e0234014 (2015). 10.1128/mBio.02340-14 PMC4324311 25626904

[14] W. Arendt, M. K. Groenewold, S. Hebecker, J. S. Dickschat, J. Moser, Identification and characterization of a periplasmic aminoacyl-phosphatidylglycerol hydrolase responsible for *Pseudomonas aeruginosa* lipid homeostasis. J. Biol. Chem. 288, 24717–24730 (2013). 23792962 10.1074/jbc.M113.482935 PMC3750168

[15] F.-J. Chen, T.-L. Lauderdale, C.-H. Lee, Y.-C. Hsu, I.-W. Huang, P.-C. Hsu, C.-S. Yang, Effect of a point mutation in *mprF* on susceptibility to daptomycin, vancomycin, and oxacillin in an MRSA clinical strain. Front. Microbiol. 9, 1086 (2018). 29887848 10.3389/fmicb.2018.01086 PMC5980971

[16] L. Friedman, J. D. Alder, J. A. Silverman, Genetic changes that correlate with reduced susceptibility to daptomycin in *Staphylococcus aureus*. Antimicrob. Agents Chemother. 50, 2137–2145 (2006). 16723576 10.1128/AAC.00039-06 PMC1479123

[17] K. Julian, K. Kosowska-Shick, C. Whitener, M. Roos, H. Labischinski, A. Rubio, L. Parent, L. Ednie, L. Koeth, T. Bogdanovich, P. C. Appelbaum, Characterization of a daptomycin-nonsusceptible vancomycin-intermediate *Staphylococcus aureus* strain in a patient with endocarditis. Antimicrob. Agents. Chemother. 51, 3445–3448 (2007). 17620372 10.1128/AAC.00559-07 PMC2043240

[18] C. J. Slavetinsky, J. N. Hauser, C. Gekeler, J. Slavetinsky, A. Geyer, A. Kraus, D. Heilingbrunner, S. Wagner, M. Tesar, B. Krismer, S. Kuhn, C. M. Ernst, A. Peschel, Sensitizing *Staphylococcus aureus* to antibacterial agents by decoding and blocking the lipid flippase MprF. eLife 11, e66376 (2022). 35044295 10.7554/eLife.66376 PMC8806190

[19] D. Song, H. Jiao, Z. Liu, Phospholipid translocation captured in a bifunctional membrane protein MprF. Nat. Commun. 12, 2927 (2021). 34006869 10.1038/s41467-021-23248-z PMC8131360

[20] I. Zimmermann, P. Egloff, C. A. Hutter, F. M. Arnold, P. Stohler, N. Bocquet, M. N. Hug, S. Huber, M. Siegrist, L. Hetemann, J. Gera, S. Gmür, P. Spies, D. Gygax, E. R. Geertsma, R. J. P. Dawson, M. A. Seeger, Synthetic single domain antibodies for the conformational trapping of membrane proteins. eLife 7, e34317 (2018). 29792401 10.7554/eLife.34317 PMC5967865

[21] J. Frauenfeld, R. Löving, J.-P. Armache, A. F. P. Sonnen, F. Guettou, P. Moberg, L. Zhu, C. Jegerschöld, A. Flayhan, J. A. G. Briggs, H. Garoff, C. Löw, Y. Cheng, P. Nordlund, A saposin-lipoprotein nanoparticle system for membrane proteins. Nat. Methods 13, 345–351 (2016). 26950744 10.1038/nmeth.3801 PMC4894539

[22] A. Flayhan, H. D. T. Mertens, Y. Ural-Blimke, M. Martinez Molledo, D. I. Svergun, C. Löw, Saposin lipid nanoparticles: A highly versatile and modular tool for membrane protein research. Structure 26, 345–355.e5 (2018). 29413323 10.1016/j.str.2018.01.007 PMC5807053

[23] J. Ahmad, J. Jiang, L. F. Boyd, A. Zeher, R. Huang, D. Xia, K. Natarajan, D. H. Margulies, Structures of synthetic nanobody–SARS-CoV-2 receptor-binding domain complexes reveal distinct sites of interaction. J. Biol. Chem. 297, 101202 (2021). 34537245 10.1016/j.jbc.2021.101202 PMC8444450

[24] J. D. Brunner, N. K. Lim, S. Schenck, A. Duerst, R. Dutzler, X-ray structure of a calcium-activated TMEM16 lipid scramblase. Nature 516, 207–212 (2014). 25383531 10.1038/nature13984

[25] S. R. Bushell, A. C. Pike, M. E. Falzone, N. J. Rorsman, C. M. Ta, R. A. Corey, T. D. Newport, J. C. Christianson, L. F. Scofano, C. A. Shintre, A. Tessitore, A. Chu, Q. Wang, L. Shrestha, S. M. M. Mukhopadhyay, J. D. Love, N. A. Burgess-Brown, R. Sitsapesan, P. J. Stansfeld, J. T. Huiskonen, P. Tammaro, A. Accardi, E. P. Carpenter, The structural basis of lipid scrambling and inactivation in the endoplasmic reticulum scramblase TMEM16K. Nat. Commun. 10, 3956 (2019). 31477691 10.1038/s41467-019-11753-1 PMC6718402

[26] M. E. Falzone, J. Rheinberger, B.-C. Lee, T. Peyear, L. Sasset, A. M. Raczkowski, E. T. Eng, A. Di Lorenzo, O. S. Andersen, C. M. Nimigean, A. Accardi, Structural basis of Ca^2+^-dependent activation and lipid transport by a TMEM16 scramblase. eLife 8, e43229 (2019). 30648972 10.7554/eLife.43229 PMC6355197

[27] T. Pomorski, A. K. Menon, Lipid flippases and their biological functions. Cell. Mol. Life Sci. 63, 2908–2921 (2006). 17103115 10.1007/s00018-006-6167-7 PMC11136118

[28] M. Malvezzi, K. K. Andra, K. Pandey, B.-C. Lee, M. E. Falzone, A. Brown, R. Iqbal, A. K. Menon, A. Accardi, Out-of-the-groove transport of lipids by TMEM16 and GPCR scramblases. Proc. Natl. Acad. Sci. U.S.A. 115, E7033–E7042 (2018). 29925604 10.1073/pnas.1806721115 PMC6065010

[29] M. E. Falzone, Z. Feng, O. E. Alvarenga, Y. Pan, B. Lee, X. Cheng, E. Fortea, S. Scheuring, A. Accardi, TMEM16 scramblases thin the membrane to enable lipid scrambling. Nat. Commun. 13, 2604 (2022). 35562175 10.1038/s41467-022-30300-z PMC9095706

[30] S. K. Ghorbal, A. Chatti, M. M. Sethom, L. Maalej, M. Mihoub, S. Kefacha, M. Feki, A. Landoulsi, A. Hassen, Changes in membrane fatty acid composition of *Pseudomonas aeruginosa* in response to UV-C radiations. Curr. Microbiol. 67, 112–117 (2013). 23463516 10.1007/s00284-013-0342-5

[31] W. Song, R. A. Corey, T. B. Ansell, C. K. Cassidy, M. R. Horrell, A. L. Duncan, P. J. Stansfeld, M. S. P. Sansom, PyLipID: A Python Package for analysis of protein–lipid interactions from molecular dynamics simulations. J. Chem. Theory Comput. 18, 1188–1201 (2022). 35020380 10.1021/acs.jctc.1c00708 PMC8830038

[32] I. Menon, T. Huber, S. Sanyal, S. Banerjee, P. Barré, S. Canis, J. D. Warren, J. Hwa, T. P. Sakmar, A. K. Menon, Opsin is a phospholipid flippase. Curr. Biol. 21, 149–153 (2011). 21236677 10.1016/j.cub.2010.12.031 PMC3057128

[33] Q.-l. Chang, S. N. Gummadi, A. K. Menon, Chemical modification identifies two populations of glycerophospholipid flippase in rat liver ER. Biochemistry 43, 10710–10718 (2004). 15311932 10.1021/bi049063a

[34] M. A. Goren, T. Morizumi, I. Menon, J. S. Joseph, J. S. Dittman, V. Cherezov, R. C. Stevens, O. P. Ernst, A. K. Menon, Constitutive phospholipid scramblase activity of a G protein-coupled receptor. Nat. Commun. 5, 5115 (2014). 25296113 10.1038/ncomms6115 PMC4198942

[35] Y. E. Li, Y. Wang, X. Du, T. Zhang, H. Y. Mak, S. E. Hancock, H. McEwen, E. Pandzic, R. M. Whan, Y. C. Aw, TMEM41B and VMP1 are scramblases and regulate the distribution of cholesterol and phosphatidylserine. J. Cell Biol. 220, e202103105 (2021). 33929485 10.1083/jcb.202103105 PMC8077175

[36] B. Ploier, A. K. Menon, A fluorescence-based assay of phospholipid scramblase activity. J. Vis. Exp. 20, 54635 (2016). 10.3791/54635 PMC5092049 27684510

[37] J. Kubelt, A. K. Menon, P. Müller, A. Herrmann, Transbilayer movement of fluorescent phospholipid analogues in the cytoplasmic membrane of *Escherichia coli*. Biochemistry 41, 5605–5612 (2002). 11969421 10.1021/bi0118714

[38] S. K. Sahu, S. N. Gummadi, Flippase activity in proteoliposomes reconstituted with *Spinacea oleracea* endoplasmic reticulum membrane proteins: Evidence of biogenic membrane flippase in plants. Biochemistry 47, 10481–10490 (2008). 18767811 10.1021/bi8014339

[39] M. Malvezzi, M. Chalat, R. Janjusevic, A. Picollo, H. Terashima, A. K. Menon, A. Accardi, Ca^2+^-dependent phospholipid scrambling by a reconstituted TMEM16 ion channel. Nat. Commun. 4, 2367 (2013). 23996062 10.1038/ncomms3367 PMC3970400

[40] B. Zhang, X. Liu, E. Lambert, G. Mas, S. Hiller, J. W. Veening, C. Perez, Structure of a proton-dependent lipid transporter involved in lipoteichoic acids biosynthesis. Nat. Struct. Mol. Biol. 27, 561–569 (2020). 32367070 10.1038/s41594-020-0425-5 PMC7611249

[41] C. Alvadia, N. K. Lim, V. Clerico Mosina, G. T. Oostergetel, R. Dutzler, C. Paulino, Cryo-EM structures and functional characterization of the murine lipid scramblase TMEM16F. eLife 8, e44365 (2019). 30785399 10.7554/eLife.44365 PMC6414204

[42] H. Roy, M. Ibba, RNA-dependent lipid remodeling by bacterial multiple peptide resistance factors. Proc. Natl. Acad. Sci. U.S.A. 105, 4667–4672 (2008). 18305156 10.1073/pnas.0800006105 PMC2290796

[43] C. J. Slavetinsky, A. Peschel, C. M. Ernst, Alanyl-phosphatidylglycerol and lysyl-phosphatidylglycerol are translocated by the same MprF flippases and have similar capacities to protect against the antibiotic daptomycin in *Staphylococcus aureus*. Antimicrob. Agents Chemother. 56, 3492–3497 (2012). 22491694 10.1128/AAC.00370-12 PMC3393434

[44] A. S. Bayer, N. N. Mishra, L. Chen, B. N. Kreiswirth, A. Rubio, S. J. Yang, Frequency and distribution of single-nucleotide polymorphisms within *mprF* in Methicillin-Resistant *Staphylococcus aureus* clinical isolates and their role in cross-resistance to daptomycin and host defense antimicrobial peptides. Antimicrob. Agents Chemother. 59, 4930–4937 (2015). 26055370 10.1128/AAC.00970-15 PMC4505294

[45] J. L. Parker, S. Newstead, Structural basis of nucleotide sugar transport across the Golgi membrane. Nature 551, 521–524 (2017). 29143814 10.1038/nature24464 PMC5701743

[46] J. N. Hauser, A. Kengmo Tchoupa, S. Zabel, K. Nieselt, C. M. Ernst, C. J. Slavetinsky, A. Peschel, PplT domain proteins–ubiquitous potential prokaryotic phospholipid translocases. bioRxiv 483950 [Preprint] (2022). 10.1101/2022.03.11.483950.

[47] I. J. Roney, D. Z. Rudner, The DedA superfamily member PetA is required for the transbilayer distribution of phosphatidylethanolamine in bacterial membranes. Proc. Natl. Acad. Sci. U.S.A. 120, e2301979120 (2023). 37155911 10.1073/pnas.2301979120 PMC10193950

[48] E. R. Geertsma, R. Dutzler, A versatile and efficient high-throughput cloning tool for structural biology. Biochemistry 50, 3272–3278 (2011). 21410291 10.1021/bi200178z

[49] I. Zimmermann, P. Egloff, C. A. Hutter, B. T. Kuhn, P. Bräuer, S. Newstead, R. J. Dawson, E. R. Geertsma, M. A. Seeger, Generation of synthetic nanobodies against delicate proteins. Nat. Protoc. 15, 1707–1741 (2020). 32269381 10.1038/s41596-020-0304-x PMC7617899

[50] A. Punjani, J. L. Rubinstein, D. J. Fleet, M. A. Brubaker, cryoSPARC: Algorithms for rapid unsupervised cryo-EM structure determination. Nat. Methods. 14, 290–296 (2017). 28165473 10.1038/nmeth.4169

[51] P. Emsley, K. Cowtan, Coot: Model-building tools for molecular graphics. Acta Crystallogr. D Biol. Crystallogr. 60, 2126–2132 (2004). 15572765 10.1107/S0907444904019158

[52] S. G. Rasmussen, H. J. Choi, J. J. Fung, E. Pardon, P. Casarosa, P. S. Chae, B. T. Devree, D. M. Rosenbaum, F. S. Thian, T. S. Kobilka, A. Schnapp, I. Konetzki, R. K. Sunahara, S. H. Gellman, A. Pautsch, J. Steyaert, W. I. Weis, B. K. Kobilka, Structure of a nanobody-stabilized active state of the β_2_ adrenoceptor. Nature 469, 175–180 (2011). 21228869 10.1038/nature09648 PMC3058308

[53] A. Waterhouse, M. Bertoni, S. Bienert, G. Studer, G. Tauriello, R. Gumienny, F. T. Heer, T. A. P. de Beer, C. Rempfer, L. Bordoli, R. Lepore, T. Schwede, SWISS-MODEL: Homology modelling of protein structures and complexes. Nucleic Acids Res. 46, W296–W303 (2018). 29788355 10.1093/nar/gky427 PMC6030848

[54] P. V. Afonine, B. K. Poon, R. J. Read, O. V. Sobolev, T. C. Terwilliger, A. Urzhumtsev, P. D. Adams, Real-space refinement in PHENIX for cryo-EM and crystallography. Acta Crystallogr. D Struct. Biol. 74, 531–544 (2018). 29872004 10.1107/S2059798318006551 PMC6096492

[55] M. J. Abraham, T. Murtola, R. Schulz, S. Páll, J. C. Smith, B. Hess, E. Lindahl, GROMACS: High performance molecular simulations through multi-level parallelism from laptops to supercomputers. SoftwareX. 1-2, 19–25 (2015).

[56] D. H. de Jong, G. Singh, W. F. D. Bennett, C. Arnarez, T. A. Wassenaar, L. V. Schäfer, X. Periole, D. P. Tieleman, S. J. Marrink, Improved parameters for the martini coarse-grained protein force field. J. Chem. Theory Comput. 9, 687–697 (2013). 26589065 10.1021/ct300646g

[57] T. A. Wassenaar, H. I. Ingólfsson, R. A. Böckmann, D. P. Tieleman, S. J. Marrink, Computational lipidomics with insane: A versatile tool for generating custom membranes for molecular simulations. J. Chem. Theory Comput. 11, 2144–2155 (2015). 26574417 10.1021/acs.jctc.5b00209

[58] G. Bussi, D. Donadio, M. Parrinello, Canonical sampling through velocity rescaling. J. Chem. Phys. 126, 014101 (2007). 17212484 10.1063/1.2408420

[59] M. Bernetti, G. Bussi, Pressure control using stochastic cell rescaling. J. Chem. Phys. 153, 114107 (2020). 32962386 10.1063/5.0020514

[60] O. N. Vickery, P. J. Stansfeld, CG2AT2: An enhanced fragment-based approach for serial multi-scale molecular dynamics simulations. J. Chem. Theory Comput. 17, 6472–6482 (2021). 34492188 10.1021/acs.jctc.1c00295 PMC8515810

[61] T. B. Ansell, W. Song, C. E. Coupland, L. Carrique, R. A. Corey, A. L. Duncan, C. K. Cassidy, M. M. G. Geurts, T. Rasmussen, A. B. Ward, C. Siebold, P. J. Stansfeld, M. S. P. Sansom, LipIDens: Simulation assisted interpretation of lipid densities in cryo-EM structures of membrane proteins. Nat Commun. 14, 7774 (2023). 38012131 10.1038/s41467-023-43392-y PMC10682427

[62] M. Parrinello, A. Rahman, Polymorphic transitions in single crystals: A new molecular dynamics method. J. Appl. Phys. 52, 7182–7190 (1981).

[63] S. Miyamoto, P. A. Kollman, Settle: An analytical version of the SHAKE and RATTLE algorithm for rigid water models. J. Comput. Chem. 13, 952–962 (1992).

[64] B. Hess, H. Bekker, H. J. C. Berendsen, J. G. E. M. Fraaije, LINCS: A linear constraint solver for molecular simulations. J. Computat. Chem. 18, 1463–1472 (1997).

[65] N. Michaud-Agrawal, E. J. Denning, T. B. Woolf, O. Beckstein, MDAnalysis: A toolkit for the analysis of molecular dynamics simulations. J. Comput. Chem. 32, 2319–2327 (2011). 21500218 10.1002/jcc.21787 PMC3144279

[66] The PyMOL Molecular Graphics System, Version 3 (Schrödinger, LLC).

[67] M. Arndt, C. Alvadia, M. S. Straub, V. Clerico Mosina, C. Paulino, R. Dutzler, Structural basis for the activation of the lipid scramblase TMEM16F. Nat. Commun. 13, 6692 (2022). 36335104 10.1038/s41467-022-34497-x PMC9637102

[68] M. E. Falzone, A. Accardi, Reconstitution of proteoliposomes for phospholipid scrambling and nonselective channel assays. Methods Mol. Biol. 2127, 207–225 (2020). 32112325 10.1007/978-1-0716-0373-4_15 PMC7297447

[69] B.-C. Lee, G. Khelashvili, M. Falzone, A. K. Menon, H. Weinstein, A. Accardi, Gating mechanism of the extracellular entry to the lipid pathway in a TMEM16 scramblase. Nat. Commun. 9, 3251 (2018). 30108217 10.1038/s41467-018-05724-1 PMC6092359

[70] B. K. Ho, F. Gruswitz, HOLLOW: Generating accurate representations of channel and interior surfaces in molecular structures. BMC Struct. Biol. 8, 49 (2008). 19014592 10.1186/1472-6807-8-49 PMC2603037

[71] M. Fonvielle, I. Li de La Sierra-Gallay, A. H. El-Sagheer, M. Lecerf, D. Patin, D. Mellal, C. Mayer, D. Blanot, N. Gale, T. Brown, H. van Tilbeurgh, M. Ethève-Quelquejeu, M. Arthur, The structure of FemX_Wv_ in complex with a peptidyl-RNA conjugate: mechanism of aminoacyl transfer from Ala-tRNA^Ala^ to peptidoglycan precursors. Angew. Chem. Int. Ed. Engl. 52, 7419–7422 (2013). 23744707 10.1002/anie.201301411

