# Supplementary material for: MprF from Pseudomonas aeruginosa is a promiscuous lipid scramblase with broad substrate specificity
Source: Sci Adv. 2025 Apr 9;11(15):eads9135. doi: 10.1126/sciadv.ads9135 (PMC11980842; doi:10.1126/sciadv.ads9135)
Supplement: Supplementary file 1 — Figs. S1 to S14 Tables S1 to S7 References [file sciadv.ads9135_sm.pdf]

Science Advances
AAAS

Supplementary Materials for

MprF from *Pseudomonas aeruginosa* is a promiscuous lipid scramblase with broad substrate specificity

Matthew T. K. Hankins *et al.*

Corresponding author: Maike Bublitz, maike.bublitz-meier@ufl.li

Sci. Adv. **11**, eads9135 (2025)
DOI: 10.1126/sciadv.ads9135

This PDF file includes:

Figs. S1 to S14
Tables S1 to S7
References

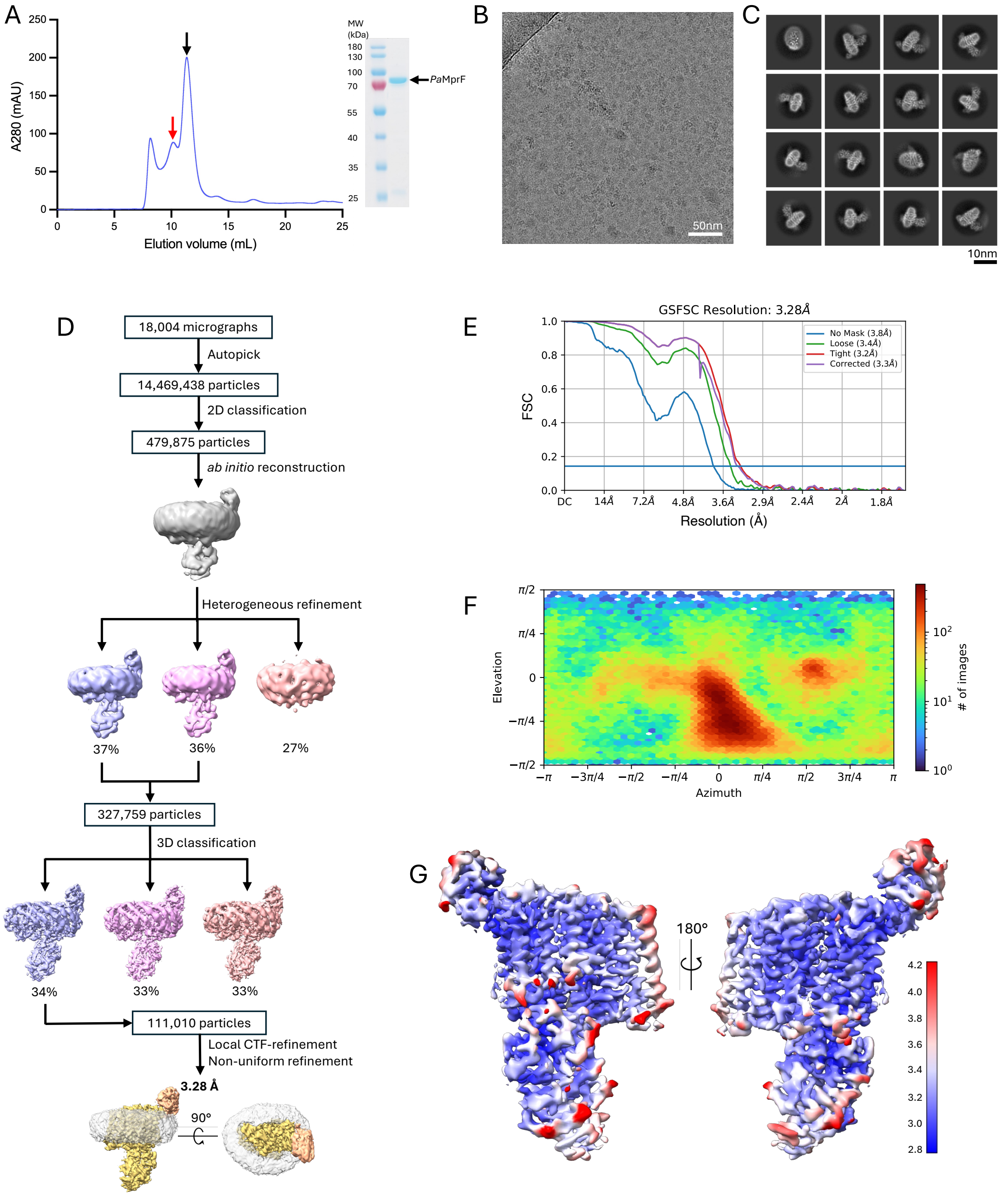

Fig. S1.

Cryo-EM processing of *Pa*MprF-Sb29 SapNPs. (A) SEC profile and SDS-PAGE gel of a typical *Pa*MprF purification. The monomer peak used for structural characterization is highlighted with a black arrow, and the higher order *Pa*MprF oligomer peak is highlighted with a red arrow. **(B)** Representative micrograph of *Pa*MprF-Sb29 SapNP particles. **(C)** Representative 2D classes of *Pa*MprF-Sb29 SapNPs with a box size of 288 pixels/23.9 nm. **(D)** Flow chart of *Pa*MprF-Sb29 SapNP processing in CryoSPARC. For the final map, *Pa*MprF = yellow, Sb29 = orange, SapNP = transparent. **(E)** Gold-standard FSC curves from the final non-uniform refinement job in CryoSPARC. At FSC = 0.143, the resolution was determined to be 3.28 Å. **(F)** Heatmap showing orientation distribution of *Pa*MprF-Sb29 SapNPs. **(G)** Final map of *Pa*MprF-Sb29 (SapNP removed for clarity), coloured according to local resolution as estimated by CryoSPARC.

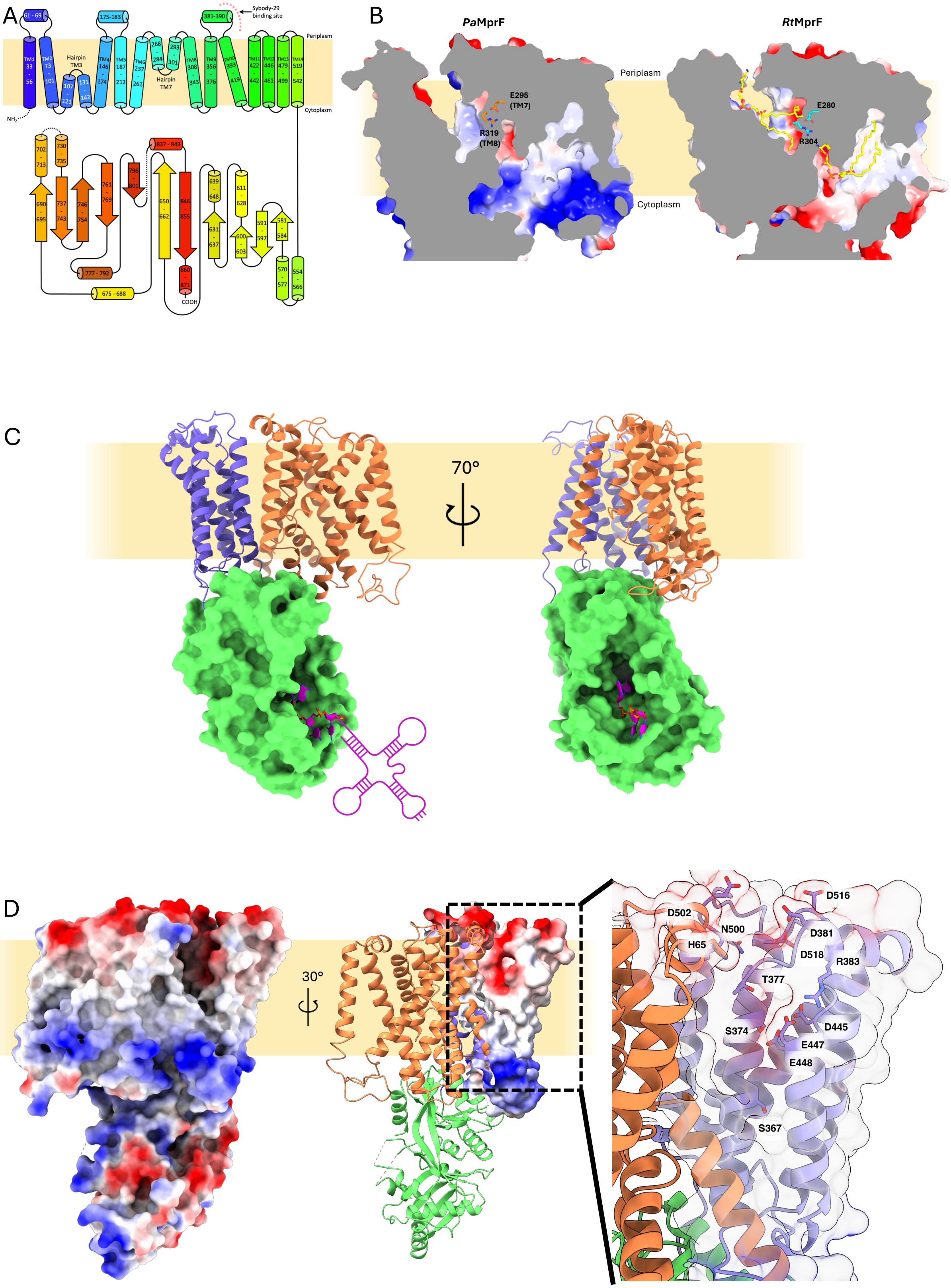

Fig. S2.

***Pa*MprF structural features. (A)** Topology diagram of *Pa*MprF (coloured as in **Fig. 1D**), with residue numbers on secondary structure elements. Black dotted lines indicate unresolved features

in model. **(B)** Clipped view of *Pa* (left) and *Rt*MprF (right) electrostatic surfaces showing the arrangement of the conserved salt bridge between glutamate (hairpin helix TM7) and arginine (TM8) residues near the centre of the membrane. The LysPG lipids present in the previously published *Rt*MprF structure are shown (PDB ID 7DUW, yellow sticks). (**C**) Side views of *Pa*MprF coloured as in Fig. 2 with the surface of the soluble domain shown. The expected tRNA binding site at the soluble domain is shown based on alignment of GNAT domain 2 of the soluble domain to FemX (71) which was co-crystallised in the presence of a tRNA-CCA analogue (shown as magenta sticks, with the rest of the tRNA molecule shown as a stylised cartoon. **(D)** *Left: Pa*MprF electrostatic surface at the periplasmic "exit cavity". *Middle*: rotated view of cavity (surface clipping through TMD1, *Pa*MprF coloured as in **Fig. 2**) highlighting stretch of polar surface extending down from periplasm into the membrane. *Right*: zoomed in view showing individual polar residues present at TMD interface groove.

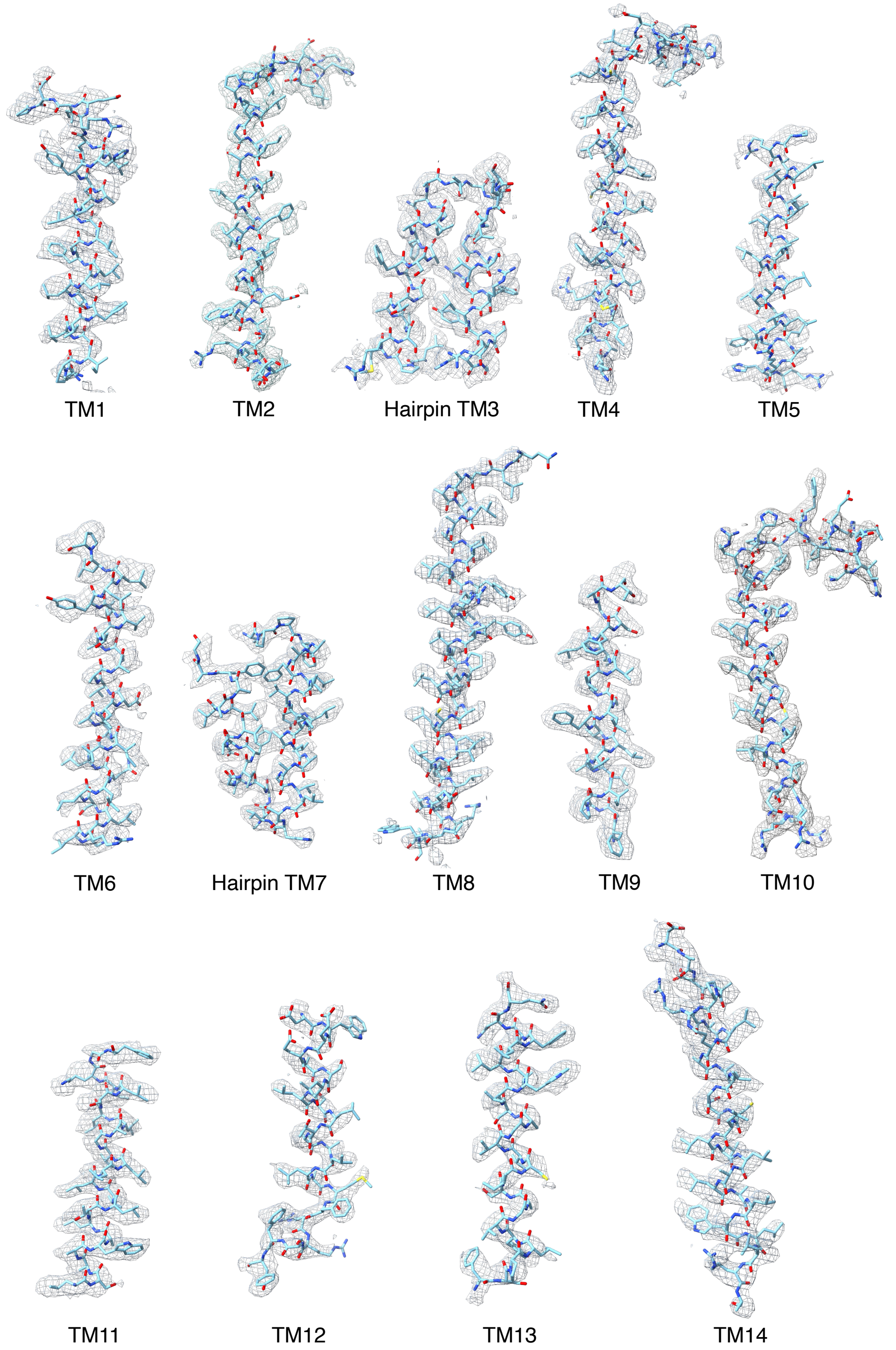

Fig. S3.

Representative cryo-EM map sections of *Pa*MprF transmembrane α-helices. Individual α-helices from the TM region of *Pa*MprF are shown (blue sticks, coloured by element) fitted to the corresponding region of the final post-processed cryo-EM map (grey mesh) sharpened with a *B*-factor of -85 Å^2. The map is contoured at 6 σ.

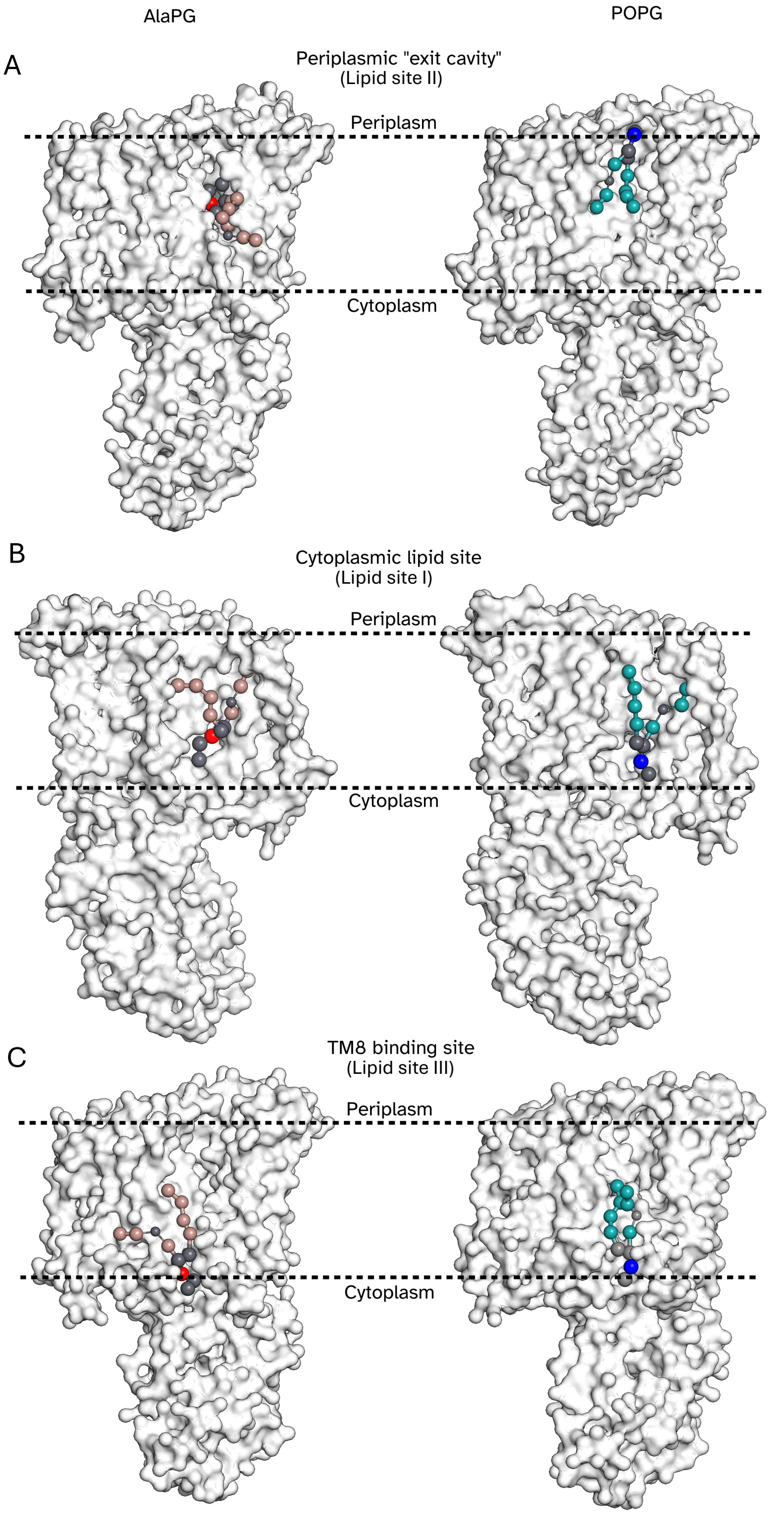

Fig. S4.

Lipid binding sites identified by coarse grain MD. Surface models of *Pa*MprF (white) with identified binding poses of AlaPG (left panels) and POPG (right panels) at **(A)** the periplasmic "exit cavity" (lipid site II), **(B)** the cytoplasmic lipid site (lipid site I), and **(C)** the TM8 site (lipid site III).

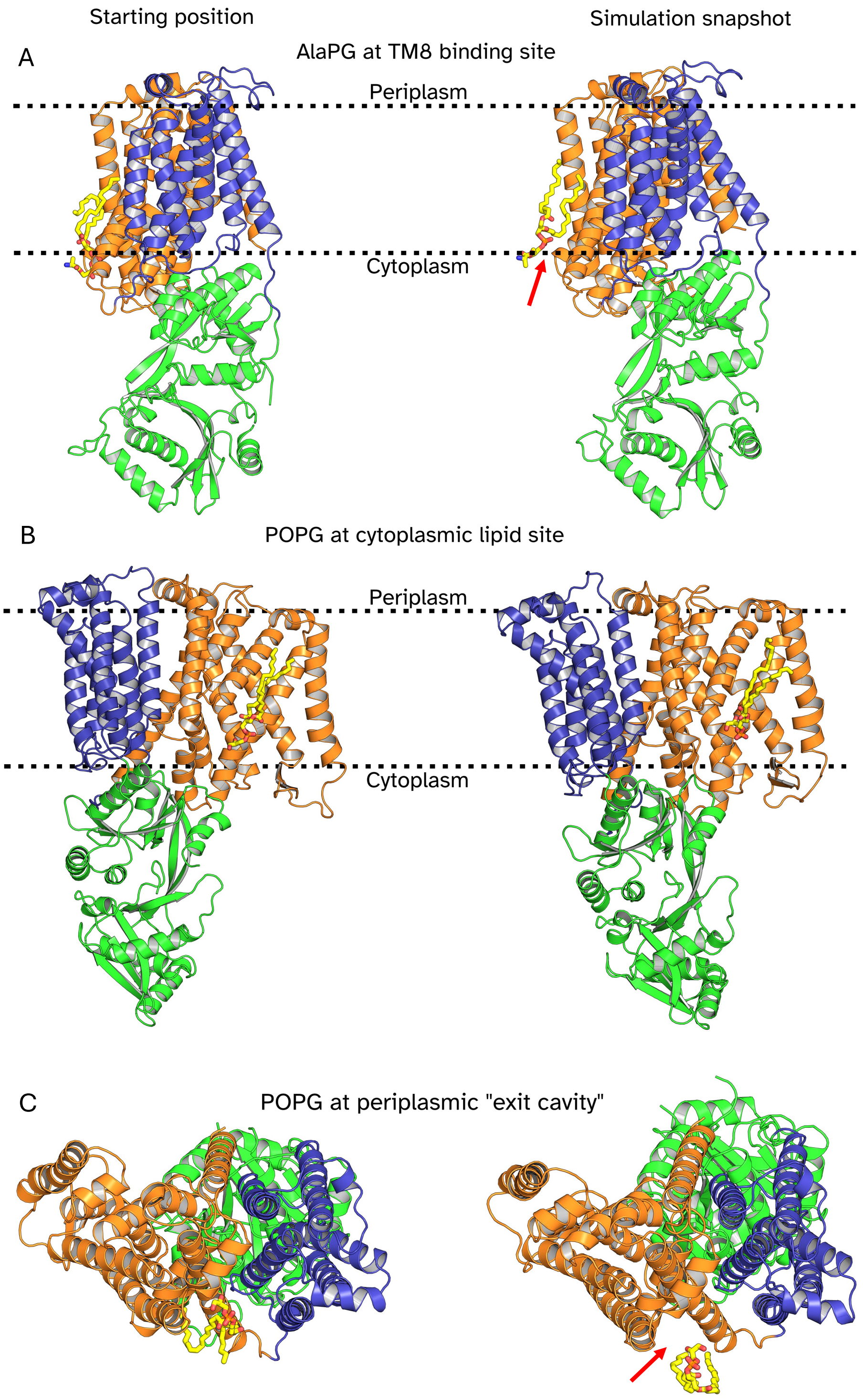

Fig. S5.

Snapshots of atomistic simulations at lipid binding sites. Starting positions (left panels) and simulation snapshots (right panels) of *Pa*MprF (coloured as in **Fig. 2**) with **(A)** AlaPG at the TM8 binding site (lipid site III), **(B)** POPG at the cytoplasmic lipid site (lipid site I), and **(C)** POPG at the periplasmic “exit cavity” (lipid site II). Detachment of lipid from the starting position is highlighted with red arrows.

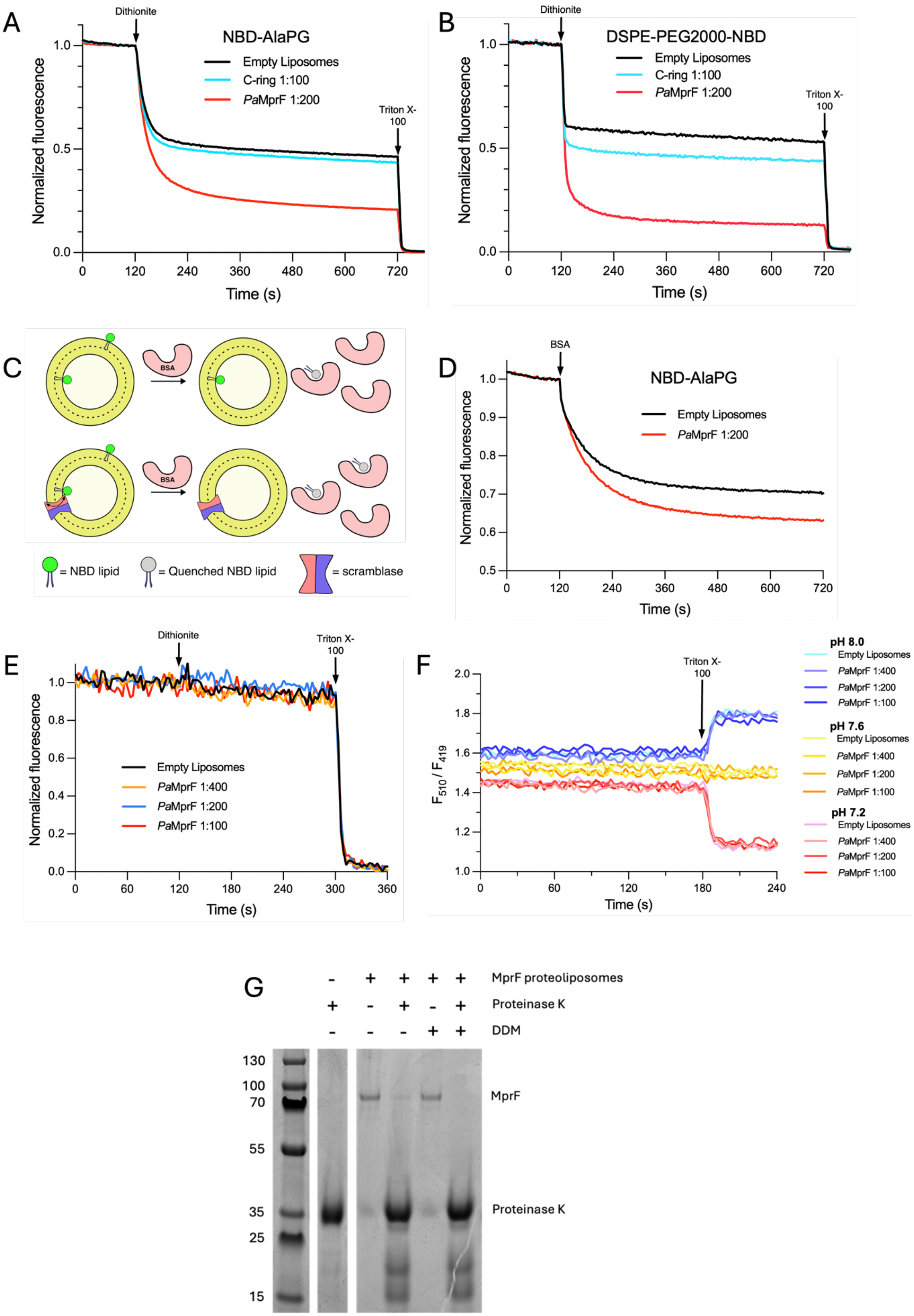
A
NBD-AlaPG
Dithionite
Triton X-100
Empty Liposomes
C-ring 1:100
PaMprF 1:200
Normalized fluorescence
Time (s)
B
DSPE-PEG2000-NBD
C
BSA
= NBD lipid
= Quenched NBD lipid
= scramblase
D
NBD-AlaPG
E
PaMprF 1:400
PaMprF 1:100
F
pH 8.0
pH 7.6
pH 7.2
F510 / F419
G
MprF proteoliposomes
Proteinase K
DDM
130
100
70
55
35
25
15
MprF
Proteinase K

Fig. S6.

Liposome control experiments. (A) Normalized fluorescence time-courses for empty (black), c-ring reconstituted (blue, 1:100 (w/w) c-ring:lipid ratio) and *Pa*MprF reconstituted (red, 1:200 (w/w) *Pa*MprF:lipid ratio) liposomes containing NBD-AlaPG. Curves are averages of three independent reconstitutions. **(B)** Same as (A), but for liposomes containing DSPE-PEG2000-NBD. **(C)** Schematic of BSA back-extraction assay; BSA extracts lipid from the outer leaflet of liposomes, quenching NBD fluorescence by ~50 % (37). **(D)** Normalized fluorescence time-courses of BSA back-extraction for empty (black) and 1:200 (w/w) *Pa*MprF:lipid reconstituted (red) NBD-AlaPG liposomes (averages of three independent reconstitutions), demonstrating an increase in surface-exposed NBD-AlaPG in *Pa*MprF proteoliposomes compared to empty liposomes. **(E)** Single replicates of normalized fluorescence time-courses for empty (black) and *Pa*MprF (orange, blue, red) liposomes at different *Pa*MprF:lipid (w/w) ratios reconstituted in the presence of NBD-glucose. **(F)** Fluorescence time-course single replicates (excitation = 455 nm, one of the pH-dependent excitation maxima of HPTS, emission = 510 nm normalized to the HPTS isosbestic point at 419 nm) of HPTS within empty (control) and *Pa*MprF reconstituted (at several *Pa*MprF:lipid (w/w) ratios) liposomes. HPTS was incorporated into liposomes at pH 7.6, and then diluted in either pH 7.2 (red traces), pH 7.6 (yellow traces), or pH 8.0 (blue traces) buffer. HPTS fluorescence only changes in experiments with a pH gradient once liposomes are solubilised by Triton-X 100 addition, indicating that both empty liposomes and proteoliposomes can maintain the pH gradient used in experiments probing the *Pa*MprF energy source without significant proton leak. **(G)** Coomassie-stained SDS-PAGE gel of *Pa*MprF liposomes (1:200 (w/w) *Pa*MprF:lipid) before and after proteinase K treatment. One sample of liposomes was solubilised with 0.5% (w/v) DDM before proteinase K addition. Following incubation with proteinase K, samples were treated with 5mM PMSF and immediately assessed via SDS-PAGE.

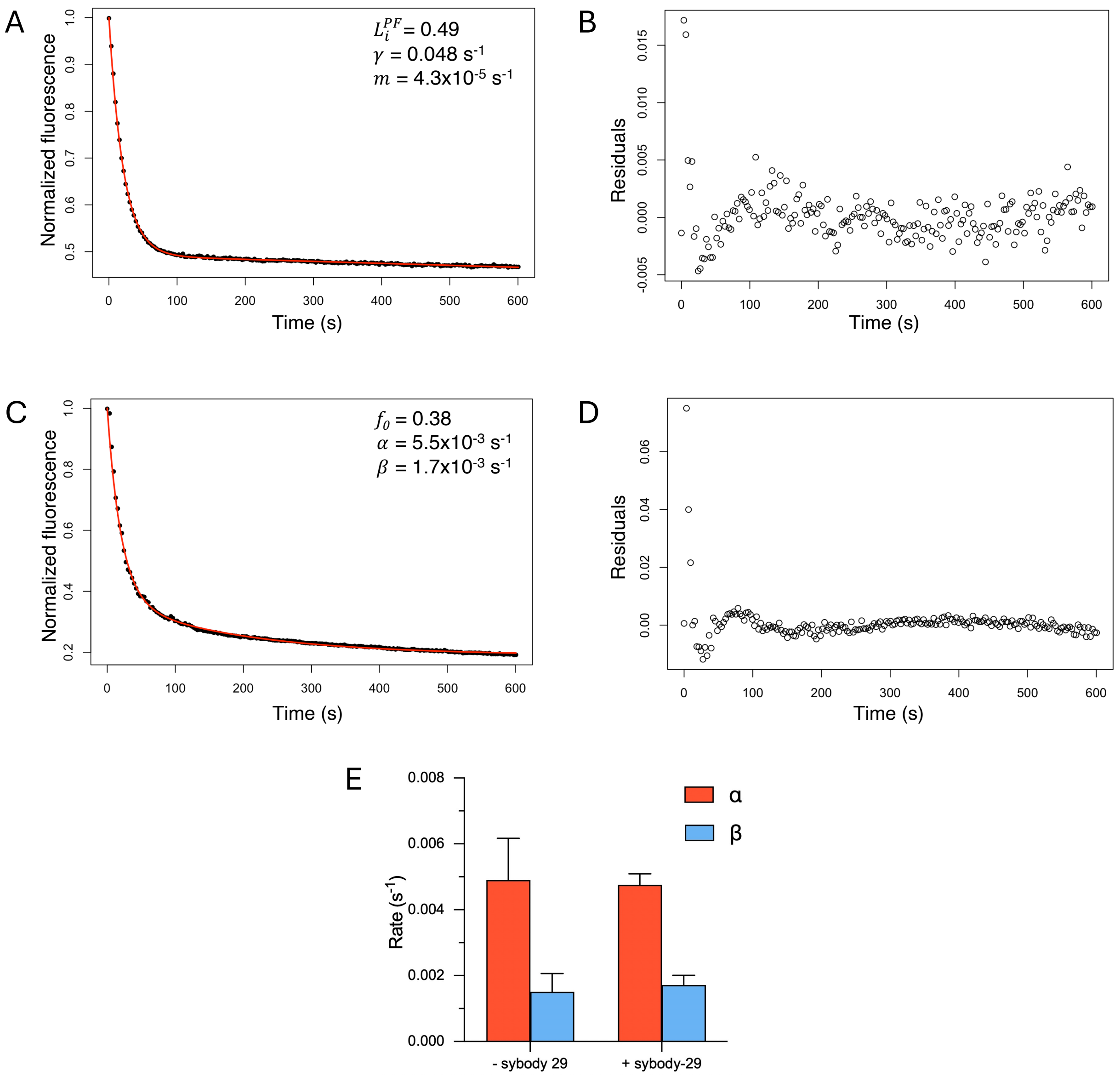

Fig. S7.

Fitting experimental liposome data to three-state model. (A) Example experimental data (black points) of a control liposome fluorescence time-course overlaid with a curve fitted from the three-state model (red line). Estimated values of L_i^{PF} and γ from this fit are shown. An additional component, -*mt*, is added to the expression for empty liposomes to represent the slow linear decrease attributed to dithionite entry into liposomes/NBD photobleaching/non-specific flip-flop of phospholipids. **(B)** Residual plot of experimental data to the fitted model from panel (A). **(C)** Example experimental data (black points) of a 1:200 (w/w) *Pa*MprF:lipid proteoliposome fluorescence time-course overlaid with a curve fitted from the three-state model (red line). Estimated values of f_0, α and β from this fit are shown. **(D)** Residual plot of experimental data to the fitted model from panel (C). **(E)** Bar chart of lipid transport rates for 1:200 (w/w) *Pa*MprF:lipid proteoliposomes in the absence (n=8) or presence (n=3) of Sybody29 (Sybody29 added at a 5x molar excess before each independent proteoliposome reconstitution).

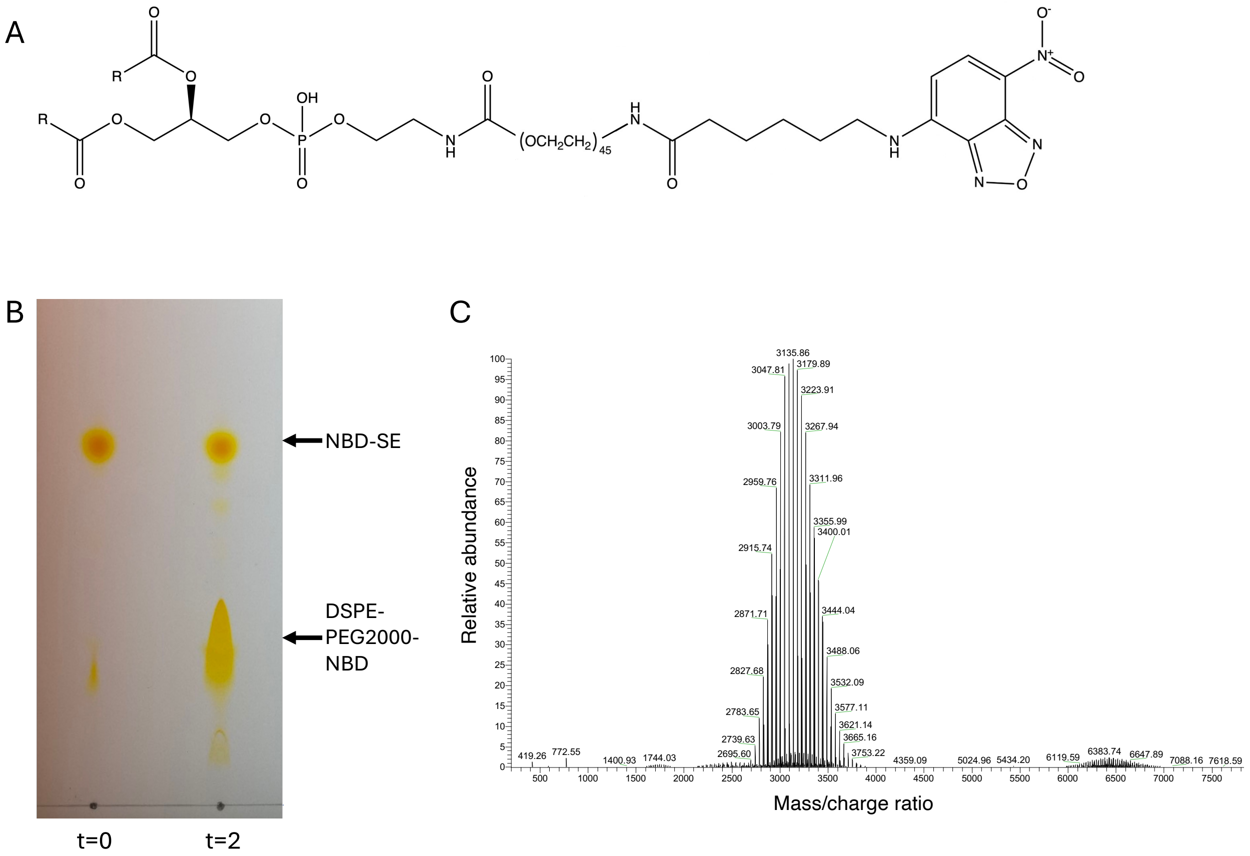

Fig. S8.

DSPE-PEG2000-NBD lipid synthesis. (A) Skeletal formula of the synthesized DSPE-PEG2000-NBD headgroup (expected MW = 3048 Da). **(B)** TLC of DSPE-PEG2000-NBD reaction mix at t = 0 and 2 hours. **(C)** DSPE-PEG2000-NBD MS spectrum. The broad set of peaks differ by 44 Da (the MW of a single PEG unit) due to the polydispersity of PEG lengths in the original DSPE-PEG2000-amine compound used for synthesis.

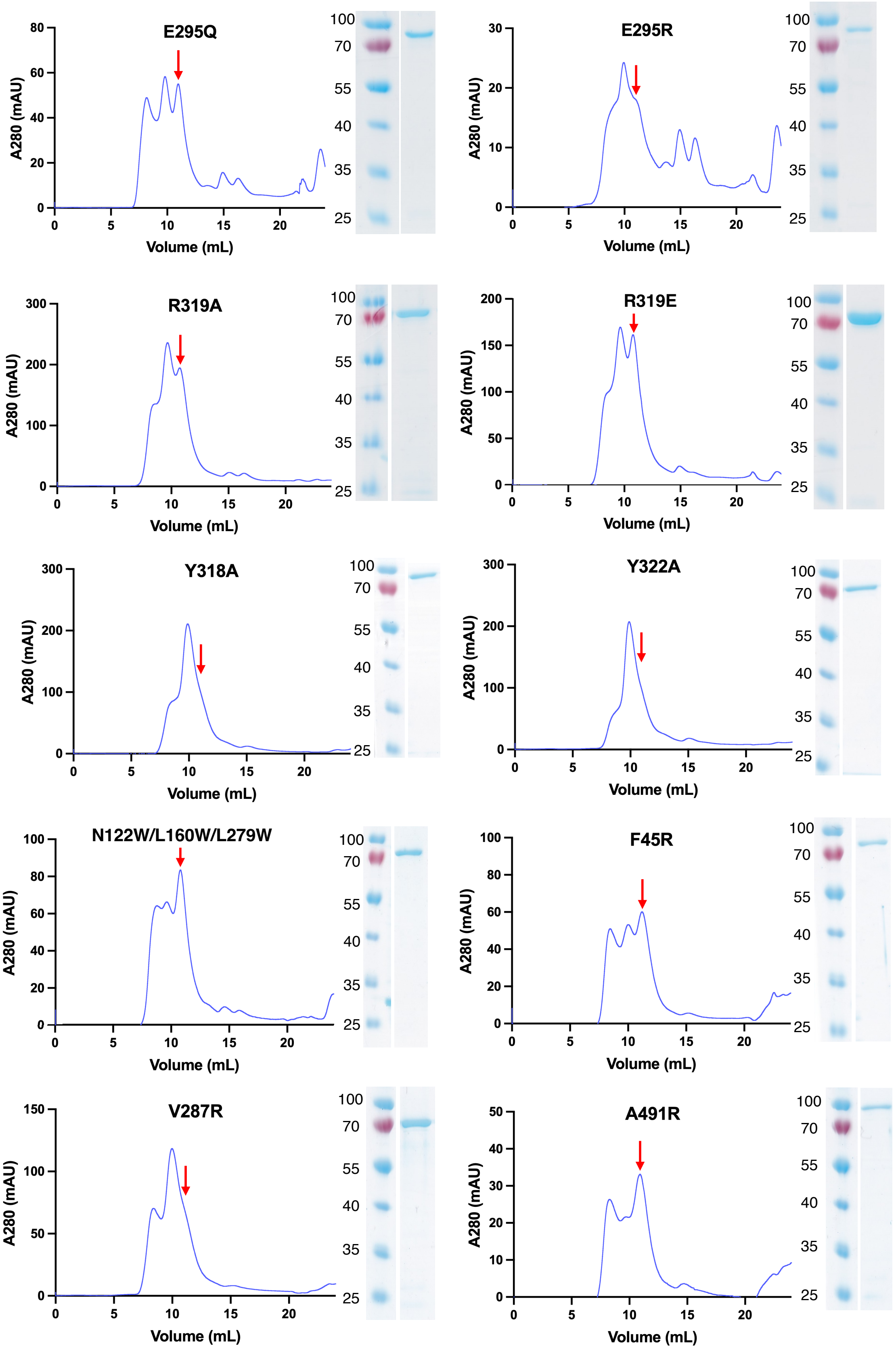

Fig. S9.
***Pa*MprF mutant purifications.** SEC chromatograms and 12 % SDS-PAGE gels of *Pa*MprF mutants discussed in the main text. The 11 mL *Pa*MprF monomer peak used for liposome experiments is highlighted with a red arrow.

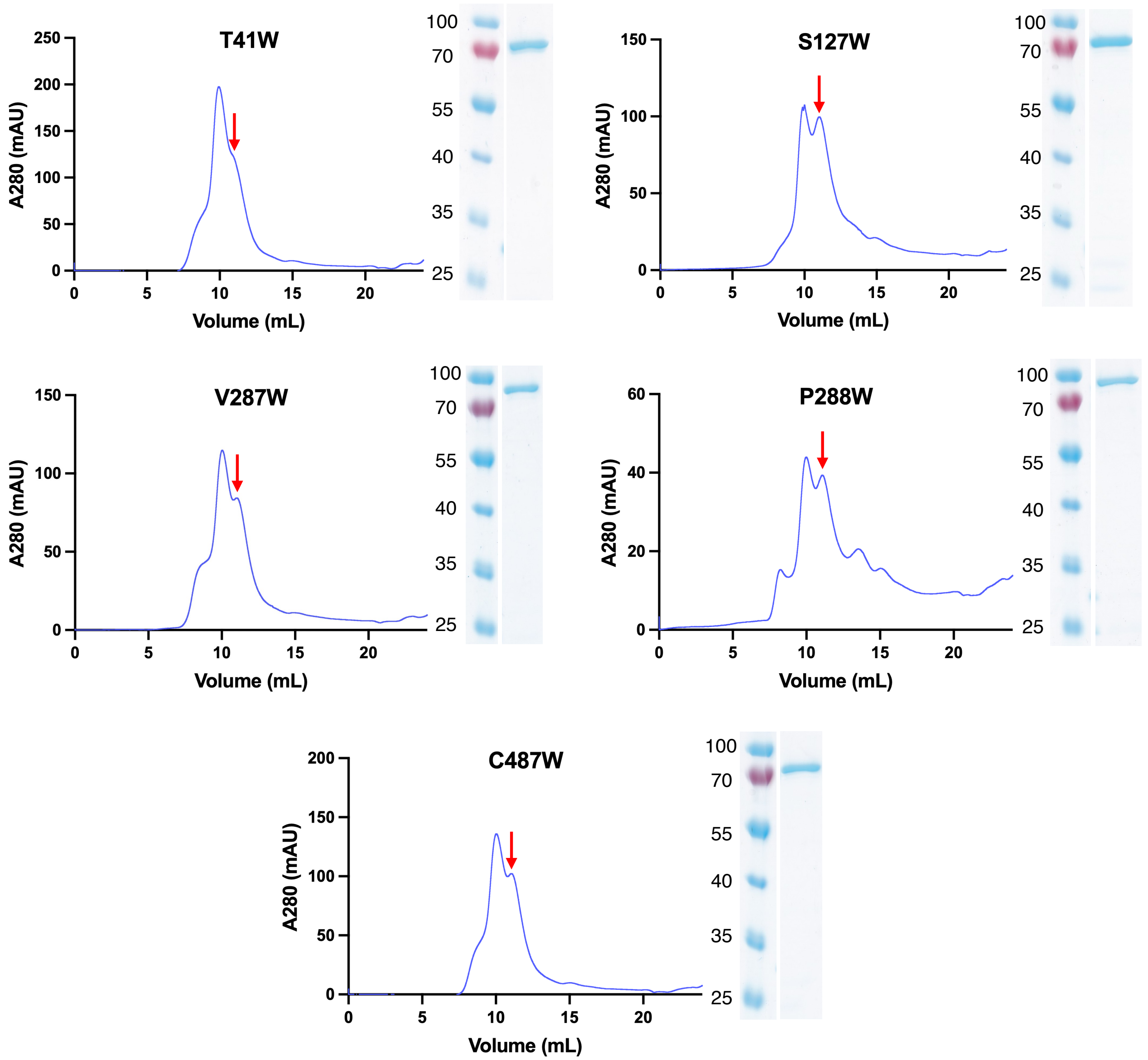

Fig. S10.
TMD interface *Pa*MprF mutant purifications. SEC chromatograms and 12 % SDS-PAGE gels of TMD interface *Pa*MprF mutants (see fig. S12). The 11 mL *Pa*MprF monomer peak used for liposome experiments is highlighted with a red arrow.

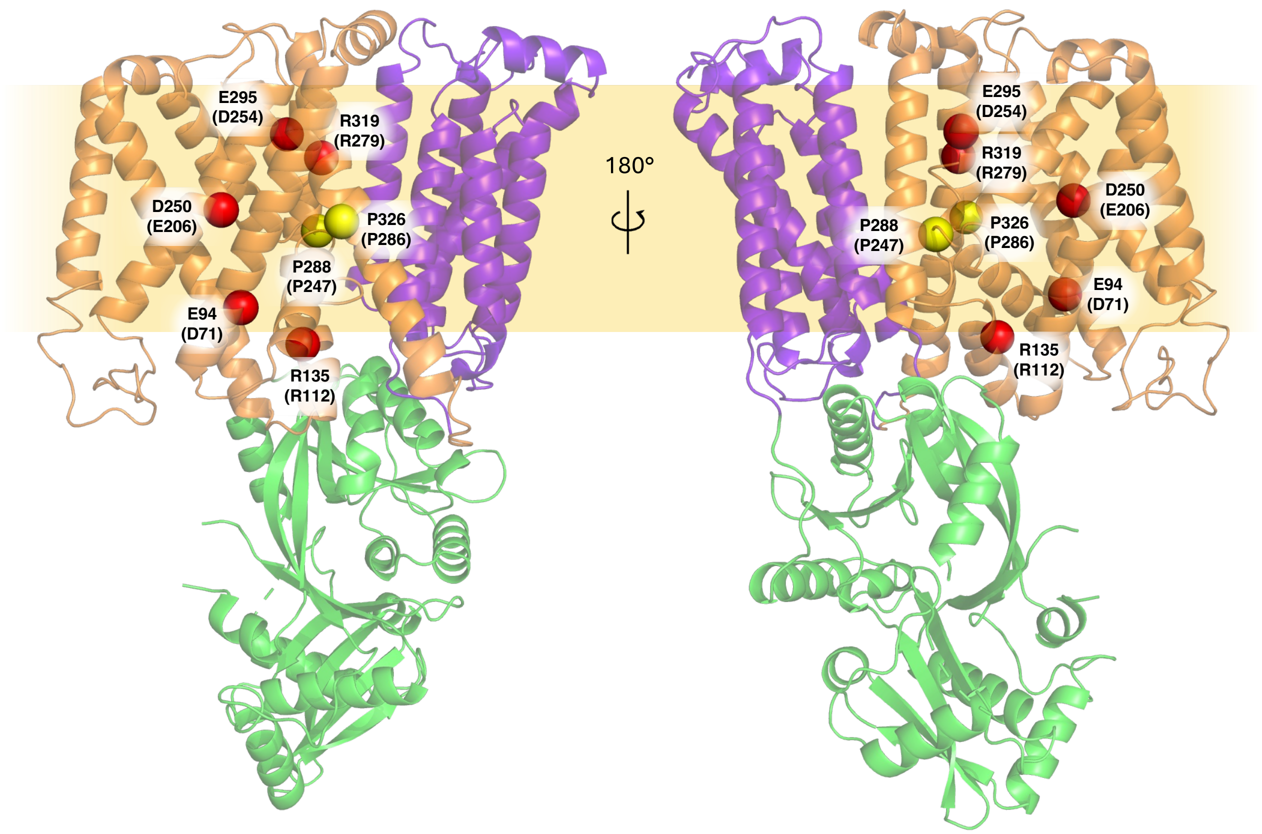

Fig. S11.

Structural context of conserved charged amino acids and proline residues in MprF. Model of *Pa*MprF (TMD1 = orange, TMD2 = purple, soluble domain = green) with C- α spheres highlighting conserved residues that were tested for Daptomycin sensitivity in a prior mutational study in *S. aureus* (*13*), *Pa*MprF residue numbers are displayed with corresponding *Sa*MprF numbers in parentheses. Red: hypersensitive to Daptomycin when mutated, yellow: moderately increased sensitivity to Daptomycin when mutated. E94 and R135 form a salt bridge that conveys structural stability to TMD1, likely influencing lipid transport indirectly. E295 had increased *in vitro* lipid transport rates when mutated. P288 had no impact on *in vitro* lipid transport activity upon mutation. Conserved residue R28 (*Sa*K14) was not resolved in our structure.

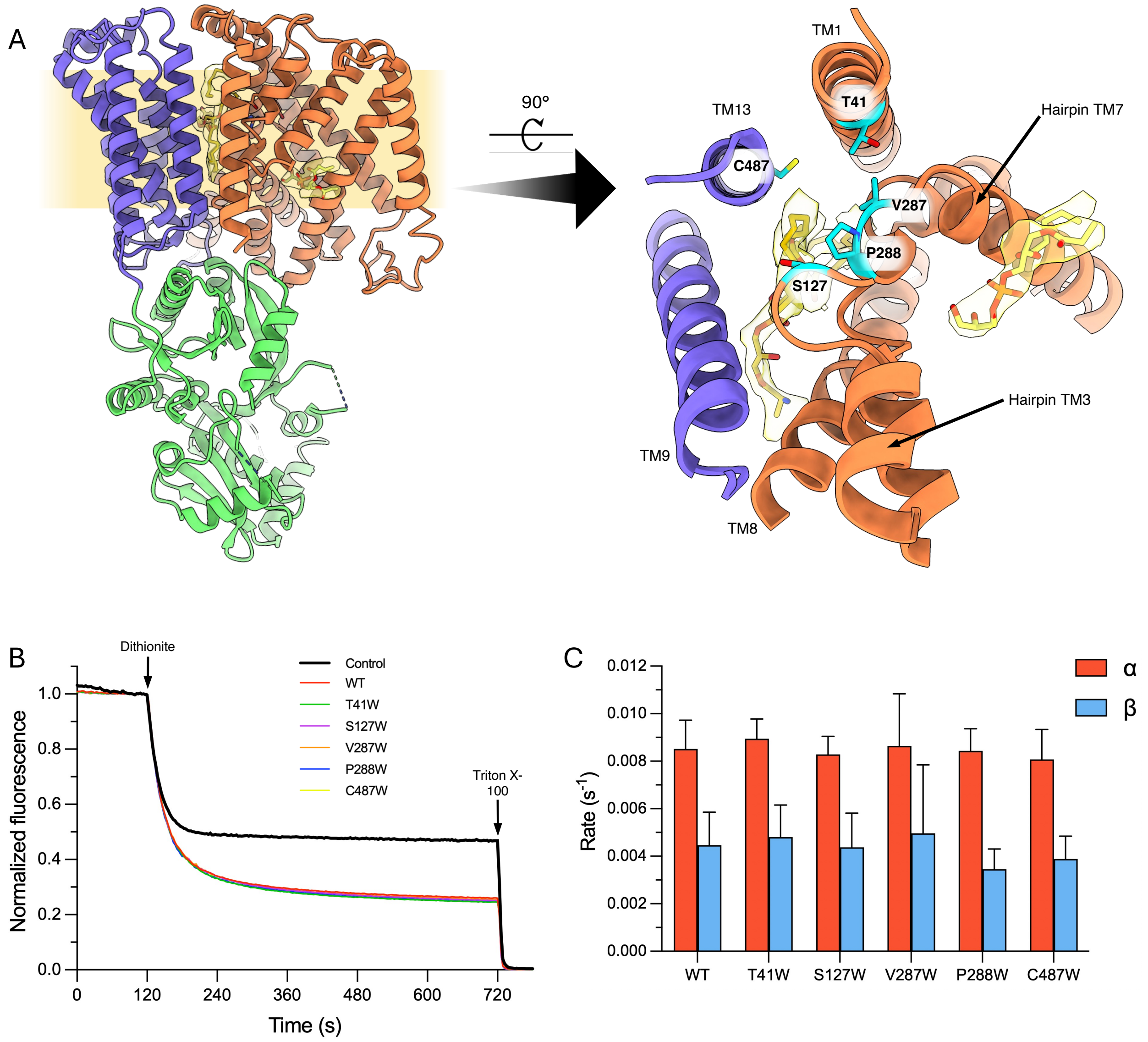

Fig. S12.

Tryptophan mutagenesis at the TMD interface does not significantly reduce lipid transport rates. (A) L*eft: Pa*MprF model (TMD1 = orange, TMD2 = purple, soluble domain = green) with cytoplasmic/periplasmic pocket lipid densities and bound lipids shown in yellow. *Right:* enlarged view of the TM domain interface of *Pa*MprF from the cytoplasmic side, clipping through the soluble domain. The five residues chosen for mutagenesis are highlighted in cyan. TM helices not involved in this interface are removed for clarity. **(B)** Normalized fluorescence time-courses for empty (black), wildtype (WT, red), T41W (green), S127W (purple), V287W (orange), P288W (blue), and C487W (yellow) 1:200 (w/w) *Pa*MprF:lipid liposomes. Curves are averages of three independent reconstitutions. **(C)** Bar charts showing mean α and β rate constants for lipid transport from fitting (error bars = standard deviation, n = 3).

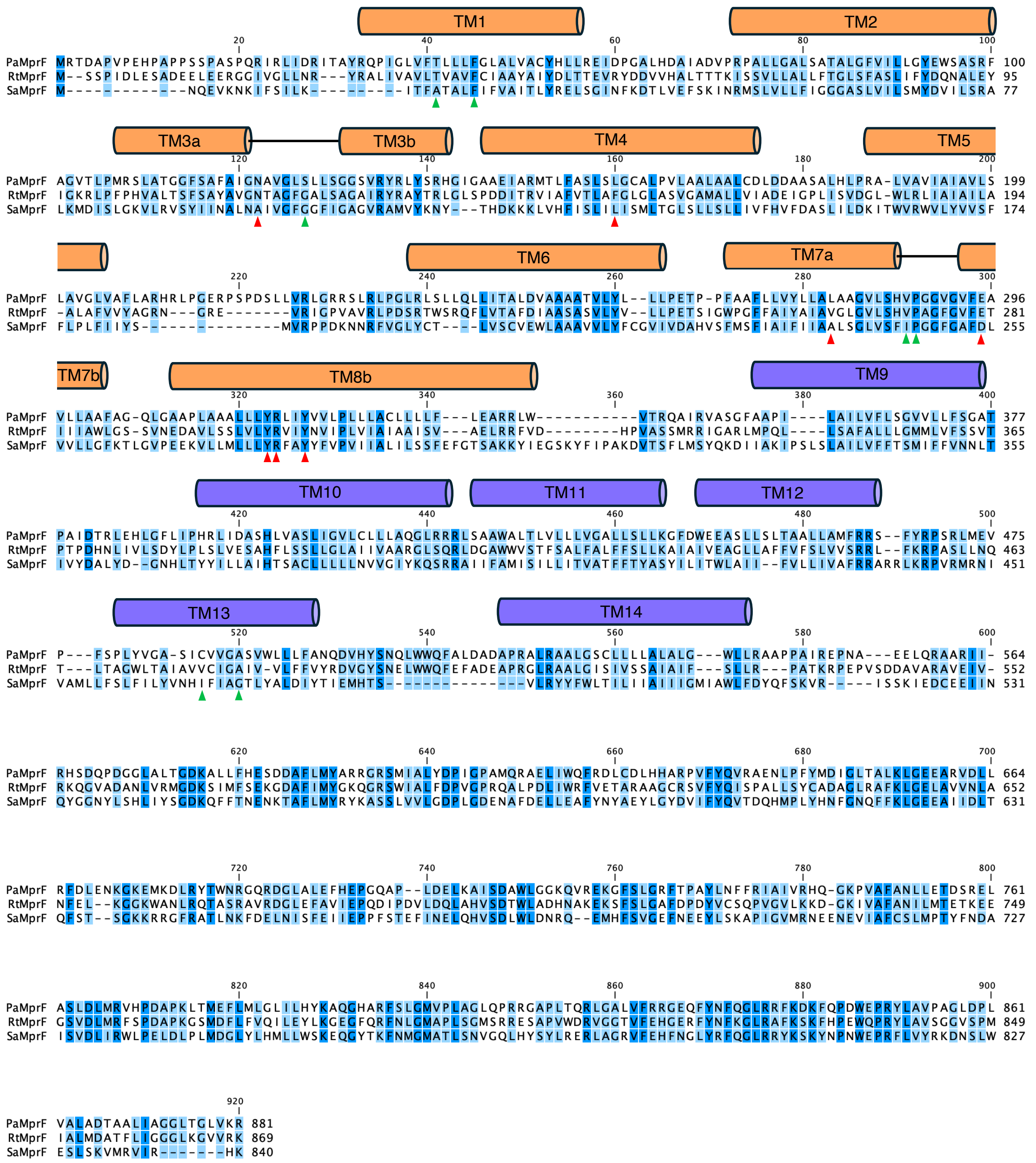

Fig. S13.

MprF alignment. Alignment of *P. aeruginosa* (*Pa*MprF), *R. tropici* (*Rt*MprF) and *S. aureus* (*Sa*MprF) MprF sequences, generated with CLC sequence viewer (QIAgen). Residues are coloured based on conservation (white – blue = least – most conserved). TM helices for *Pa*MprF are depicted above the corresponding sequence (orange = TMD1, purple = TMD2). The residues mutated in *Pa*MprF in this study to investigate lipid transport are highlighted as triangles below sequences (red = cytoplasmic lipid binding pocket, green = TMD interface).

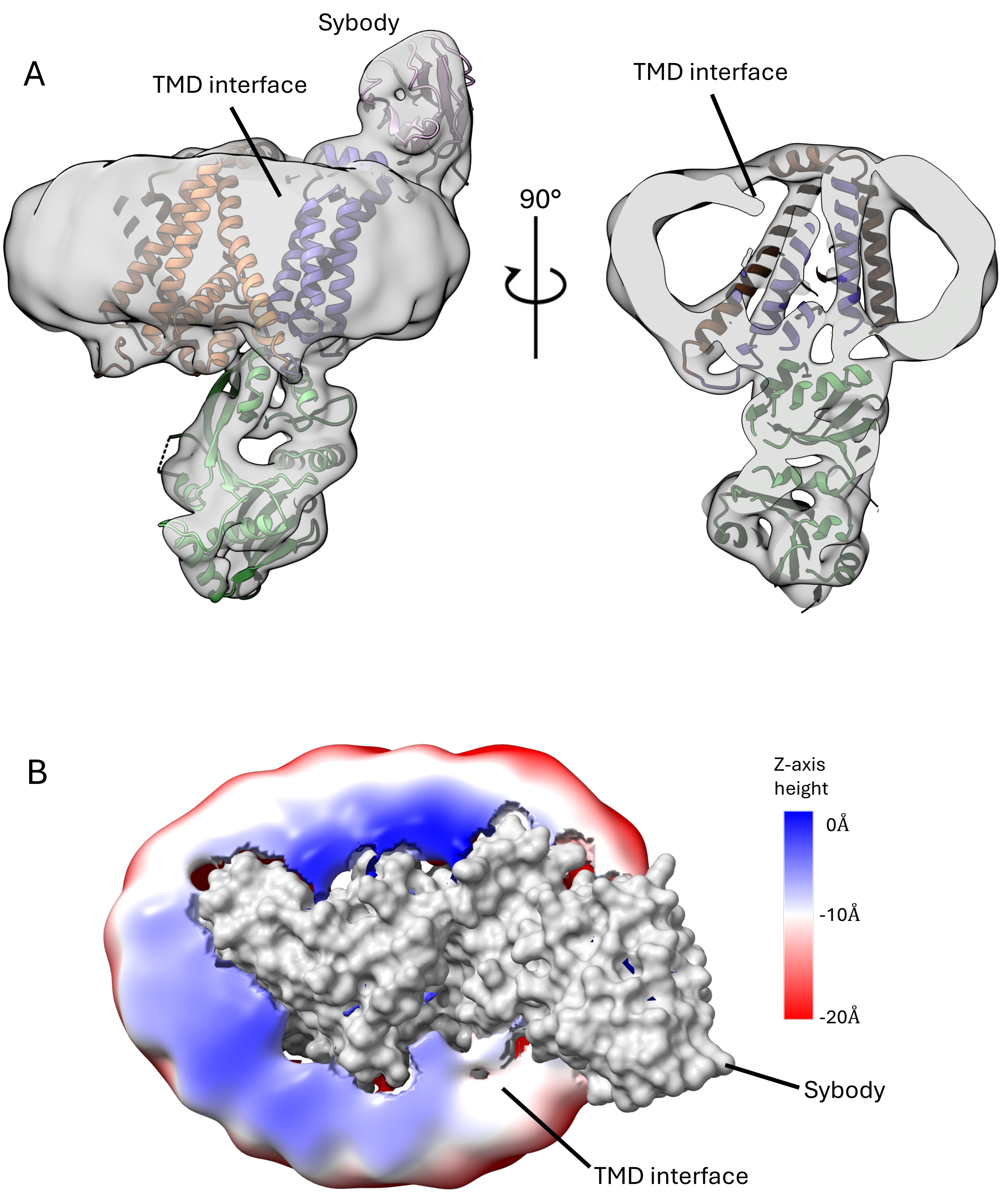

Fig. S14.
Deformation of SapNP surrounding *Pa*MprF. (A) Cryo-EM map of *Pa*MprF-SapNPs, aligned to the *Pa*MprF-Sb29 model (coloured as in Fig. 2). The map has been low-pass filtered to allow better visualisation of the surrounding SapNP. A rotated view is shown on the right clipping through TM2 to show the SapNP deformation at the TMD interface. **(B)** Top view (from the periplasm) of the low-pass filtered cryo-EM map of the SapNP, coloured according to z-axis height away from A63 (a residue in the *Pa*MprF TM domain that sits at the periplasmic surface of the membrane). The surface of the *Pa*MprF-Sb29 model is shown in grey.

Table S1.
***Pa*MprF mutant purification yields.**

*Pa*MprF construct	Yield (mg protein/L cell culture)
WT	0.31
E295Q	0.13
E295R	0.04
R319A	0.28
R319E	0.20
Y318A	0.18
Y322A	0.15
N122W/L160W/L279W	0.14
F45R	0.16
V287R	0.13
A491R	0.10
T41W	0.15
S127W	0.16
V287W	0.10
P288W	0.09
C487W	0.11

Table S2.
List of primers used in this study.

**Primer Name**	**Primer sequence (5’-3’)**
*Pa*MprF_WT_Fw	ATATATGCTCTTCTAGTCGCACCGACGCTCCCGTTCCAGAACAT
*Pa*MprF_WT_Rv	TATATAGCTCTTCATGCGCGTTTCACCAATCCAGTCAGGCCGCC
*Pa*MprF_T41W_Fw	CGGCCTGGTCTTCTGGTTGCTGCTGTTCGGC
*Pa*MprF_T41W_Rv	GCCGAACAGCAGCAACCAGAAGACCAGGCCG
*Pa*MprF_F45R_Fw	CCTTGCTGCTGCGCGGCCTGGCGC
*Pa*MprF_F45R_Rv	GCGCCAGGCCGCGCAGCAGCAAGG
*Pa*MprF_N122W_Fw	GCCTTCGCCATCGGCTGGGCGGTAGGCCTGTCG
*Pa*MprF_N122W_Rv	CGACAGGCCTACCGCCCAGCCGATGGCGAAGGC
*Pa*MprF_S127W_Fw	GCGGTAGGCCTGTGGCTGCTTTCC
*Pa*MprF_S127W_Rv	CGGAAAGCAGCCACAGGCCTACCGC
*Pa*MprF_L160W_Fw	GCCAGCCTTTCCTGGGGCTGCGCACTGC
*Pa*MprF_L160W_Rv	GCAGTGCGCAGCCCCAGGAAAGGCTGGC
*Pa*MprF_L279W_Fw	CTACCTGCTCGCCTGGGCCGCCGGCGTAC
*Pa*MprF_L279W_Rv	GTACGCCGGCGGCCCAGGCGAGCAGGTAG
*Pa*MprF_V287R_Fw	GCGTACTCAGCCACCGCCCGGGCGGAGTCG
*Pa*MprF_V287R_Rv	CGACTCCGCCCGGGCGGTGGCTGAGTACG
*Pa*MprF_V287W_Fw	GCGTACTCAGCCACTGGCCGGGCGGAGTCG
*Pa*MprF_V287W_Rv	CGACTCCGCCCGGCCAGTGGCTGAGTACG
*Pa*MprF_P288W_Fw	CGTACTCAGCCACGTATGGGGCGGAGTCG
*Pa*MprF_P288W_Rv	CGACTCCGCCCCATACGTGGCTGAGTACG
*Pa*MprF_E295Q_Fw	GTCGGGGTGTTCCAGGCGGTGCTGC
*Pa*MprF_E295Q_Rv	GCAGCACCGCCTGGAACACCCCGAC
*Pa*MprF_E295R_Fw	GAGTCGGGGTGTTCCGCGCGGTGCTGCTGG
*Pa*MprF_E295R_Rv	CCAGCAGCACCGCGCGGAACACCCCGACTC
*Pa*MprF_Y318A_Fw	CGCGGCCCTGCTCTTGGCCCGCTTGATTTATGTGG
*Pa*MprF_Y318A_Rv	CACATAAATCAAGCGGGCCAAGAGCAGGGCCGCG
*Pa*MprF_R319A_Fw	CGGCCCTGCTCTTGTACGCCTTGATTTATGTGGTGC
*Pa*MprF_R319A_Rv	GCACCACATAAATCAAGGCGTACAAGAGCAGGGCC
*Pa*MprF_R319E_Fw	CGGCCCTGCTCTTGTACGAATTGATTTATGTGGTGC
*Pa*MprF_R319E_Rv	GCACCACATAAATCAATTCGTACAAGAGCAGGGCCGC
*Pa*MprF_Y322A_Fw	CTTGTACCGCTTGATTGCGGTGGTGCTGCCACTGC
*Pa*MprF_Y322A_Rv	GTGGCAGCACCACCGCAATCAAGCGGTACAAGAGC
*Pa*MprF_C487W_Fw	CCAGCATCTGGGTGGTCGGCGC
*Pa*MprF_C487W_Rv	GCGCCGACCACCCAGATGCTGGC
*Pa*MprF_A491R_Fw	GCGTGGTCGGCCGCTCGGTCTGGC
*Pa*MprF_A491R_Rv	CAGCCAGACCGAGCGGCCGACCACGC

Table S3.

Details of sybody selections against full-length *Pa*MprF for each sybody library. Enrichment factors for phage display steps are calculated by dividing total count of DNA pulled down against *Pa*MprF by DNA pulled down against the negative control protein AcrB.

**Sybody library**	**Phage display #1 enrichment factor**	**Phage display #2 enrichment factor**	**ELISA hits (out of total analysed)**	**Number of purified sybodies (out of total sequenced)**
Concave	1.2	37.0	78/95	14/14
Loop	1.4	10.7	40/95	8/8
Convex	0.7	12.1	65/95	9/10

Table S4.
***Pa*MprF-Sybody29 Cryo-EM data collection, reconstruction and model building statistics.**

**Data collection:**	
Magnification	105,000
Voltage (kV)	300
Defocus range (µm)	-0.75 to -2.25
Pixel size (Å)	0.83
Electron dose (e^-/Å^2)	42.4
Dose rate (e^-/Å^2/s)	7.85
**Reconstruction:**	
Number of particles	111,010
Symmetry	C1
Resolution at FSC=0.143 (Å)	3.28
Map sharpening *B*-factor (Å^2)	-85
**Model composition:**	
Non-hydrogen atoms	7104
Protein residues	924
**Rms deviations:**	
Bonds (Å)	0.007
Angles (°)	0.678
**Molprobity validation:**	
Clashscore, all atoms	4.44
Molprobity score	1.55
Poor rotamers (%)	0.11
Ramachandran Plot (% favoured)	95.31
Ramachandran Plot (% allowed)	4.69
Ramachandran Plot (% outliers)	0.00

Table S5.

Lipid transport rate constants for assessment of lipid specificity. For f_0, α and β, standard deviations are given in parentheses. P-values are shown for unpaired t-tests compared to NBD-AlaPG values.

**NBD lipid**	L_i^{PF}	γ **(s^{-1})**	f_0	f_0 **p-value**	α **(s^{-1})**	α **p-value**	β **(s^{-1})**	β **p-value**	**n**
NBD-AlaPG	0.44	0.047	0.30 (0.030)	-	0.031 ($4.0x10^3$)	-	0.011 ($3.9x10^3$)	-	3
NBD-LysPG	0.50	0.051	0.32 (0.045)	0.495	0.021 ($3.2x10^3$)	0.026	$5.9x10^3$ ($2.7x10^3$)	0.140	3
NBD-PG	0.53	0.047	0.28 ($9.5x10^3$)	0.437	0.029 ($7.5x10^3$)	0.676	$9.4x10^3$ ($2.7x10^3$)	0.612	3
NBD-PC	0.58	0.041	0.26 (0.022)	0.150	0.013 ($2.8x10^3$)	0.003	$1.7x10^3$ ($5.8x10^3$)	0.015	3
NBD-PE	0.51	0.041	0.303 ($8.4x10^3$)	0.703	0.029 (0.011)	0.832	8.0x103 ($7.3x10^3$)	0.949	3

Table S6.

Lipid transport rate constants for analysis of common activators. For α and β, standard deviations are given in parentheses. P-values are shown for unpaired t-tests compared to values in the absence of an activator.

**Energy source**	α **(s^{-1})**	α **p-value**	β **(s^{-1})**	β **p-value**	**n**
None	$4.9x10^3$ ($1.3x10^3$)	-	$1.5x10^3$ ($5.5x10^4$)	-	8
Low pH	$4.6x10^3$ ($6.9x10^4$)	0.733	$1.3x10^3$ ($2.2x10^4$)	0.493	3
High pH	$4.5x10^3$ ($6.1x10^4$)	0.610	$9.6x10^4$ ($3.0x10^4$)	0.144	3
NaCl	$4.0x10^3$ ($4.7x10^4$)	0.255	$8.2x10^4$ ($1.8x10^4$)	0.069	3
ATP	$4.1x10^3$ ($6.5x10^4$)	0.315	$8.6x10^4$ ($2.8x10^4$)	0.090	3
Ca	$4.1x10^3$ ($4.7x10^4$)	0.306	$8.2x10^4$ ($1.8x10^4$)	0.072	3
ProtK	$8.3x10^4$ ($9.1x10^5$)	<0.001	$7.3x10^4$ ($1.2x10^4$)	0.043	3
Sybody-29	$4.8x10^3$ ($3.3x10^4$)	0.849	$1.7x10^3$ ($3.0x10^4$)	0.568	3

Table S7.

Lipid transport rate constants for analysis of *Pa*MprF mutants. For α and β, standard deviations are given in parentheses. P-values are shown for unpaired t-tests compared to WT values. Each experiment included an internal WT control to account for minor variations in rate constants between experiments.

Related Fig.	Mutant	α (s^{-1})	α p-value	β (s^{-1})	β p-value	n
Fig. 8C	WT	6.x710^3 (1.0x10^3)	-	2.5x10^3 (6.3x10^4)	-	3
	E295Q	7.8x10^3 (4.5x10^4)	0.171	2.6x10^3 (3.2x10^4)	0.892	3
	E295R	1.2x10^2 (2.6x10^3)	0.031	3.2x10^3 (1.7x10^3)	0.541	3
	R319A	6.4x10^3 (6.5x10^4)	0.705	2.0x10^3 (3.3x10^4)	0.247	3
	R319E	8.2x10^3 (1.6x10^3)	0.244	2.9x10^3 (1.0x10^3)	0.560	3
	Y318A	7.0x10^3 (1.3x10^3)	0.718	2.5x10^3 (1.0x10^3)	0.969	3
	Y322A	5.6x10^3 (7.5x10^4)	0.167	1.9x10^3 (6.3x10^4)	0.200	3
	N122W/L160W/L279W	5.7x10^3 (2.0x10^3)	0.504	1.8x10^3 (1.1x10^3)	0.426	3
Fig. 8F	WT	7.6x10^3 (1.2x10^3)	-	3.0x10^3 (1.4x10^3)	-	3
	F45R	1.2x10^2 (2.7x10^3)	0.049	4.8x10^3 (1.2x10^3)	0.167	3
	V287R	8.3x10^3 (1.8x10^3)	0.617	2.4x10^3 (9.3x10^4)	0.539	3
	A491R	1.2x10^2 (3.1x10^4)	0.002	4.2x10^3 (1.8x10^4)	0.217	3
Fig. S12C	WT	8.5x10^3 (1.2x10^3)	-	4.5x10^3 (1.4x10^3)	-	3
	T41W	8.9x10^3 (8.3x10^4)	0.641	4.8x10^3 (1.3x10^3)	0.772	3
	S127W	8.3x10^3 (7.6x10^4)	0.794	4.4x10^3 (1.4x10^3)	0.942	3
	V287W	8.7x10^3 (2.2x10^3)	0.931	5.0x10^3 (2.9x10^3)	0.799	3
	P288W	8.4x10^3 (9.2x10^4)	0.933	3.5x10^3 (8.5x10^4)	0.343	3
	C487W	8.1x10^3 (1.3x10^3)	0.684	3.9x10^3 (9.5x10^4)	0.586	3

REFERENCES AND NOTES

1. A. Peschel, R.W. Jack, M. Otto, L.V. Collins, P. Staubitz, G. Nicholson, H. Kalbacher, W.F. Nieuwenhuizen, G. Jung, A. Tarkowski, K. P. M. van Kessel, J. A. G. van Strijp, Staphylococcus aureus resistance to human defensins and evasion of neutrophil killing via the novel virulence factor MprF is based on modification of membrane lipids with l-lysine. *J. Exp. Med.* **193**, 1067–1076 (2001).

2. P. Vinuesa, F. Neumann-Silkow, C. Pacios-Bras, H. P. Spaink, E. Martínez-Romero, D. Werner, Genetic analysis of a pH-regulated operon from Rhizobium tropici CIAT899 involved in acid tolerance and nodulation competitiveness. *Mol. Plant Microbe Interact.* **16**, 159–168 (2003).

3. S. Klein, C. Lorenzo, S. Hoffmann, J. M. Walther, S. Storbeck, T. Piekarski, B. J. Tindall, V. Wray, M. Nimtz, J. Moser, Adaptation of Pseudomonas aeruginosa to various conditions includes tRNA-dependent formation of alanyl-phosphatidylglycerol. *Mol. Microbiol.* **71**, 551–565 (2009).

4. P.W. Simcock, M. Bublitz, F. Cipcigan, M. G. Ryadnov, J. Crain, P. J. Stansfeld, M. S. P. Sansom, Membrane binding of antimicrobial peptides is modulated by lipid charge modification. *J. Chem. Theory Comput.* **17**, 1218–1228 (2021).

5. M. G. Macfarlane, Characterization of lipoamino-acids as O-amino-acid esters of phosphatidyl-glycerol. *Nature.* **196**, 136–138 (1962).

6. H. Roy, Tuning the properties of the bacterial membrane with aminoacylated phosphatidylglycerol. *IUBMB Life* **61**, 940–953 (2009).

7. C. M. Ernst, P. Staubitz, N. N. Mishra, S.-J. Yang, G. Hornig, H. Kalbacher, A. S. Bayer, D. Kraus, A. Peschel, The bacterial defensin resistance protein MprF consists of separable domains for lipid lysinylation and antimicrobial peptide repulsion. *PLOS Pathog.* **5**, e1000660 (2009).

8. W. J. Lennarz, J. A. Nesbitt III, J. Reiss, The participation of sRNA in the enzymatic synthesis of OL-lysyl phosphatidylgylcerol in Staphylococcus aureus. *Proc. Natl. Acad. Sci. U.S.A.* **55**, 934–941 (1966).

9. S. Hebecker, W. Arendt, I. U. Heinemann, J. H. Tiefenau, M. Nimtz, M. Rohde, D. Söll, J. Moser, Alanyl-phosphatidylglycerol synthase: mechanism of substrate recognition during tRNA-dependent lipid modification in *Pseudomonas aeruginosa*. *Mol. Microbiol.* **80**, 935–950 (2011).

10. S. Hebecker, J. Krausze, T. Hasenkampf, J. Schneider, M. Groenewold, J. Reichelt, D. Jahn, D. W. Heinz, J. Moser, Structures of two bacterial resistance factors mediating tRNA-dependent aminoacylation of phosphatidylglycerol with lysine or alanine. *Proc. Natl. Acad. Sci. U.S.A.* **112**, 10691–10696 (2015).

11. J. A. Nesbitt III, W. J. Lennarz, Participation of aminoacyl transfer ribonucleic acid in aminoacyl phosphatidylglycerol synthesis. I. Specificity of lysyl phosphatidylglycerol synthetase. *J. Biol. Chem.* **243**, 3088–3095 (1968).

12. C. M. Ernst, A. Peschel, Broad-spectrum antimicrobial peptide resistance by MprF-mediated aminoacylation and flipping of phospholipids. *Mol. Microbiol.* **80**, 290–299 (2011).

13. C. M. Ernst, S. Kuhn, C. J. Slavetinsky, B. Krismer, S. Heilbronner, C. Gekeler, D. Kraus, S. Wagner, A. Peschel, The lipid-modifying multiple peptide resistance factor is an oligomer consisting of distinct interacting synthase and flippase subunits. *mBio* **6**, e0234014 (2015).

14. W. Arendt, M. K. Groenewold, S. Hebecker, J. S. Dickschat, J. Moser, Identification and characterization of a periplasmic aminoacyl-phosphatidylglycerol hydrolase responsible for *Pseudomonas aeruginosa* lipid homeostasis. *J. Biol. Chem.* **288**, 24717–24730 (2013).

15. F.-J. Chen, T.-L. Lauderdale, C.-H. Lee, Y.-C. Hsu, I.-W. Huang, P.-C. Hsu, C.-S. Yang, Effect of a point mutation in *mprF* on susceptibility to daptomycin, vancomycin, and oxacillin in an MRSA clinical strain. *Front. Microbiol.* **9**, 1086 (2018).

16. L. Friedman, J. D. Alder, J. A. Silverman, Genetic changes that correlate with reduced susceptibility to daptomycin in *Staphylococcus aureus*. *Antimicrob. Agents Chemother.* **50**, 2137–2145 (2006).

17. K. Julian, K. Kosowska-Shick, C. Whitener, M. Roos, H. Labischinski, A. Rubio, L. Parent, L. Ednie, L. Koeth, T. Bogdanovich, P. C. Appelbaum, Characterization of a daptomycin-

nonsusceptible vancomycin-intermediate *Staphylococcus aureus* strain in a patient with endocarditis. *Antimicrob. Agents. Chemother.* **51**, 3445–3448 (2007).

18. C. J. Slavetinsky, J. N. Hauser, C. Gekeler, J. Slavetinsky, A. Geyer, A. Kraus, D. Heilingbrunner, S. Wagner, M. Tesar, B. Krismer, S. Kuhn, C. M. Ernst, A. Peschel, Sensitizing *Staphylococcus aureus* to antibacterial agents by decoding and blocking the lipid flippase MprF. *eLife* **11**, e66376 (2022).

19. D. Song, H. Jiao, Z. Liu, Phospholipid translocation captured in a bifunctional membrane protein MprF. *Nat. Commun.* **12**, 2927 (2021).

20. I. Zimmermann, P. Egloff, C.A. Hutter, F.M. Arnold, P. Stohler, N. Bocquet, M.N. Hug, S. Huber, M. Siegrist, L. Hetemann, J. Gera, S. Gmür, P. Spies, D. Gygax, E. R. Geertsma, R. J. P. Dawson, M. A. Seeger, Synthetic single domain antibodies for the conformational trapping of membrane proteins. *eLife* **7**, e34317 (2018).

21. J. Frauenfeld, R. Löving, J.-P. Armache, A.F.P. Sonnen, F. Guettou, P. Moberg, L. Zhu, C. Jegerschöld, A. Flayhan, J.A.G. Briggs, H. Garoff, C. Löw, Y. Cheng, P. Nordlund, A saposin-lipoprotein nanoparticle system for membrane proteins. *Nat. Methods* **13**, 345–351 (2016).

22. A. Flayhan, H. D. T. Mertens, Y. Ural-Blimke, M. Martinez Molledo, D. I. Svergun, C. Löw, Saposin lipid nanoparticles: A highly versatile and modular tool for membrane protein research. *Structure* **26**, 345–355.e5 (2018).

23. J. Ahmad, J. Jiang, L.F. Boyd, A. Zeher, R. Huang, D. Xia, K. Natarajan, D. H. Margulies, Structures of synthetic nanobody–SARS-CoV-2 receptor-binding domain complexes reveal distinct sites of interaction. *J. Biol. Chem.* **297**, 101202 (2021).

24. J. D. Brunner, N. K. Lim, S. Schenck, A. Duerst, R. Dutzler, X-ray structure of a calcium-activated TMEM16 lipid scramblase. *Nature* **516**, 207–212 (2014).

25. S. R. Bushell, A. C. Pike, M. E. Falzone, N. J. Rorsman, C. M. Ta, R. A. Corey, T. D. Newport, J. C. Christianson, L. F. Scofano, C. A. Shintre, A. Tessitore, A. Chu, Q. Wang, L. Shrestha, S. M. M. Mukhopadhyay, J. D. Love, N. A. Burgess-Brown, R. Sitsapesan, P. J. Stansfeld, J. T.

Huiskonen, P. Tammaro, A. Accardi, E. P. Carpenter, The structural basis of lipid scrambling and inactivation in the endoplasmic reticulum scramblase TMEM16K. *Nat. Commun.* **10**, 3956 (2019).

26. M. E. Falzone, J. Rheinberger, B.-C. Lee, T. Peyear, L. Sasset, A. M. Raczkowski, E. T. Eng, A. Di Lorenzo, O. S. Andersen, C. M. Nimigean, A. Accardi, Structural basis of Ca^{2+}-dependent activation and lipid transport by a TMEM16 scramblase. *eLife* **8**, e43229 (2019).

27. T. Pomorski, A. K. Menon, Lipid flippases and their biological functions. *Cell. Mol. Life Sci.* **63**, 2908–2921 (2006).

28. M. Malvezzi, K. K. Andra, K. Pandey, B.-C. Lee, M. E. Falzone, A. Brown, R. Iqbal, A. K. Menon, A. Accardi, Out-of-the-groove transport of lipids by TMEM16 and GPCR scramblases. *Proc. Natl. Acad. Sci. U.S.A.* **115**, E7033–E7042 (2018).

29. M.E. Falzone, Z. Feng, O.E. Alvarenga, Y. Pan, B. Lee, X. Cheng, E. Fortea, S. Scheuring, A. Accardi, TMEM16 scramblases thin the membrane to enable lipid scrambling. *Nat. Commun.* **13**, 2604 (2022).

30. S.K. Ghorbal, A. Chatti, M.M. Sethom, L. Maalej, M. Mihoub, S. Kefacha, M. Feki, A. Landoulsi, A. Hassen, Changes in membrane fatty acid composition of *Pseudomonas aeruginosa* in response to UV-C radiations. *Curr. Microbiol.* **67**, 112–117 (2013).

31. W. Song, R. A. Corey, T. B. Ansell, C. K. Cassidy, M. R. Horrell, A. L. Duncan, P. J. Stansfeld, M. S. P. Sansom, PyLipID: A Python Package for analysis of protein–lipid interactions from molecular dynamics simulations. *J. Chem. Theory Comput.* **18**, 1188–1201 (2022).

32. I. Menon, T. Huber, S. Sanyal, S. Banerjee, P. Barré, S. Canis, J.D. Warren, J. Hwa, T.P. Sakmar, A. K. Menon, Opsin is a phospholipid flippase. *Curr. Biol.* **21**, 149–153 (2011).

33. Q.-l. Chang, S. N. Gummadi, A. K. Menon, Chemical modification identifies two populations of glycerophospholipid flippase in rat liver ER. *Biochemistry* **43**, 10710–10718 (2004).

34. M. A. Goren, T. Morizumi, I. Menon, J. S. Joseph, J. S. Dittman, V. Cherezov, R. C. Stevens, O. P. Ernst, A. K. Menon, Constitutive phospholipid scramblase activity of a G protein-coupled receptor. *Nat. Commun.* **5**, 5115 (2014).

35. Y. E. Li, Y. Wang, X. Du, T. Zhang, H. Y. Mak, S. E. Hancock, H. McEwen, E. Pandzic, R. M. Whan, Y. C. Aw, TMEM41B and VMP1 are scramblases and regulate the distribution of cholesterol and phosphatidylserine. *J. Cell Biol.* **220**, e202103105 (2021).

36. B. Ploier, A. K. Menon, A fluorescence-based assay of phospholipid scramblase activity. *J. Vis. Exp.* **20**, 54635 (2016).

37. J. Kubelt, A. K. Menon, P. Müller, A. Herrmann, Transbilayer movement of fluorescent phospholipid analogues in the cytoplasmic membrane of *Escherichia coli. Biochemistry* **41**, 5605–5612 (2002).

38. S.K. Sahu and S.N. Gummadi, Flippase activity in proteoliposomes reconstituted with Spinacea oleracea endoplasmic reticulum membrane proteins: Evidence of biogenic membrane flippase in plants. *Biochemistry* **47**, 10481–10490 (2008).

39. M. Malvezzi, M. Chalat, R. Janjusevic, A. Picollo, H. Terashima, A. K. Menon, A. Accardi, Ca^{2+}-dependent phospholipid scrambling by a reconstituted TMEM16 ion channel. *Nat. Commun.* **4**, 2367 (2013).

40. B. Zhang, X. Liu, E. Lambert, G. Mas, S. Hiller, J. W. Veening, C. Perez, Structure of a proton-dependent lipid transporter involved in lipoteichoic acids biosynthesis. *Nat. Struct. Mol. Biol.* **27**, 561–569 (2020).

41. C. Alvadia, N. K. Lim, V. Clerico Mosina, G. T. Oostergetel, R. Dutzler, C. Paulino, Cryo-EM structures and functional characterization of the murine lipid scramblase TMEM16F. *eLife* **8**, e44365 (2019).

42. H. Roy, M. Ibba, RNA-dependent lipid remodeling by bacterial multiple peptide resistance factors. *Proc. Natl. Acad. Sci. U.S.A.* **105**, 4667–4672 (2008).

43. C. J. Slavetinsky, A. Peschel, C. M. Ernst, Alanyl-phosphatidylglycerol and lysyl-phosphatidylglycerol are translocated by the same MprF flippases and have similar capacities to protect against the antibiotic daptomycin in *Staphylococcus aureus*. *Antimicrob. Agents Chemother.* **56**, 3492–3497 (2012).

44. A. S. Bayer, N. N. Mishra, L. Chen, B. N. Kreiswirth, A. Rubio, S. J. Yang, Frequency and distribution of single-nucleotide polymorphisms within mprF in Methicillin-Resistant *Staphylococcus aureus* clinical isolates and their role in cross-resistance to daptomycin and host defense antimicrobial peptides. *Antimicrob. Agents Chemother.* **59**, 4930–4937 (2015).

45. J. L. Parker, S. Newstead, Structural basis of nucleotide sugar transport across the Golgi membrane. *Nature* **551**, 521–524 (2017).

46. J. N. Hauser, A. Kengmo Tchoupa, S. Zabel, K. Nieselt, C. M. Ernst, C. J. Slavetinsky, A. Peschel, PplT domain proteins–ubiquitous potential prokaryotic phospholipid translocases. bioRxiv 483950 [Preprint] (2022). https://doi.org/10.1101/2022.03.11.483950.

47. I. J. Roney, D.Z. Rudner, The DedA superfamily member PetA is required for the transbilayer distribution of phosphatidylethanolamine in bacterial membranes. *Proc. Natl. Acad. Sci. U.S.A.* **120**, e2301979120 (2023).

48. E. R. Geertsma, R. Dutzler, A versatile and efficient high-throughput cloning tool for structural biology. *Biochemistry* **50**, 3272–3278 (2011).

49. I. Zimmermann, P. Egloff, C. A. Hutter, B. T. Kuhn, P. Bräuer, S. Newstead, R. J. Dawson, E. R. Geertsma, M. A. Seeger, Generation of synthetic nanobodies against delicate proteins. *Nat. Protoc.* **15**, 1707–1741 (2020).

50. A. Punjani, J. L. Rubinstein, D. J. Fleet, M. A. Brubaker, cryoSPARC: Algorithms for rapid unsupervised cryo-EM structure determination. *Nat. Methods.* **14**, 290–296 (2017).

51. P. Emsley, K. Cowtan, Coot: Model-building tools for molecular graphics. *Acta Crystallogr. D Biol. Crystallogr.* **60**, 2126–2132 (2004).

52. S. G. Rasmussen, H. J. Choi, J. J. Fung, E. Pardon, P. Casarosa, P. S. Chae, B. T. Devree, D. M. Rosenbaum, F. S. Thian, T. S. Kobilka, A. Schnapp, I. Konetzki, R. K. Sunahara, S. H. Gellman, A. Pautsch, J. Steyaert, W. I. Weis, B. K. Kobilka, Structure of a nanobody-stabilized active state of the β_2 adrenoceptor. *Nature* **469**, 175–180 (2011).

53. A. Waterhouse, M. Bertoni, S. Bienert, G. Studer, G. Tauriello, R. Gumienny, F. T. Heer, T. A. P. de Beer, C. Rempfer, L. Bordoli, R. Lepore, T. Schwede, SWISS-MODEL: Homology modelling of protein structures and complexes. *Nucleic Acids Res.* **46**, W296–W303 (2018).

54. P. V. Afonine, B. K. Poon, R. J. Read, O. V. Sobolev, T. C. Terwilliger, A. Urzhumtsev, P. D. Adams, Real-space refinement in PHENIX for cryo-EM and crystallography. *Acta Crystallogr. D Struct. Biol.* **74**, 531–544 (2018).

55. M. J. Abraham, T. Murtola, R. Schulz, S. Páll, J. C. Smith, B. Hess, E. Lindahl, GROMACS: High performance molecular simulations through multi-level parallelism from laptops to supercomputers. *SoftwareX.* **1-2**, 19–25 (2015).

56. D. H. de Jong, G. Singh, W. F. D. Bennett, C. Arnarez, T. A. Wassenaar, L. V. Schäfer, X. Periole, D. P. Tieleman, S. J. Marrink, Improved parameters for the martini coarse-grained protein force field. *J. Chem. Theory Comput.* **9**, 687–697 (2013).

57. T. A. Wassenaar, H. I. Ingólfsson, R. A. Böckmann, D. P. Tieleman, S. J. Marrink, Computational lipidomics with insane: A versatile tool for generating custom membranes for molecular simulations. *J. Chem. Theory Comput.* **11**, 2144–2155 (2015).

58. G. Bussi, D. Donadio, M. Parrinello, Canonical sampling through velocity rescaling. *J. Chem. Phys.* **126**, 014101 (2007).

59. M. Bernetti, G. Bussi, Pressure control using stochastic cell rescaling. *J. Chem. Phys.* **153**, 114107 (2020).

60. O.N. Vickery, P.J. Stansfeld, CG2AT2: An enhanced fragment-based approach for serial multi-scale molecular dynamics simulations. *J. Chem. Theory Comput.* **17**, 6472–6482 (2021).

61. T. B. Ansell, W. Song, C. E. Coupland, L. Carrique, R. A. Corey, A. L. Duncan, C. K. Cassidy, M. M. G. Geurts, T. Rasmussen, A. B. Ward, C. Siebold, P. J. Stansfeld, M. S. P. Sansom, LipIDens: Simulation assisted interpretation of lipid densities in cryo-EM structures of membrane proteins. *Nat Commun.* **14**, 7774 (2023).

62. M. Parrinello, A. Rahman, Polymorphic transitions in single crystals: A new molecular dynamics method. *J. Appl. Phys.* **52**, 7182–7190 (1981).

63. S. Miyamoto, P.A. Kollman, Settle: An analytical version of the SHAKE and RATTLE algorithm for rigid water models. *J. Comput. Chem.* **13**, 952–962 (1992).

64. B. Hess, H. Bekker, H. J. C. Berendsen, J. G. E. M. Fraaije, LINCS: A linear constraint solver for molecular simulations. *J. Computat. Chem.* **18**, 1463–1472 (1997).

65. N. Michaud-Agrawal, E. J. Denning, T. B. Woolf, O. Beckstein, MDAnalysis: A toolkit for the analysis of molecular dynamics simulations. *J. Comput. Chem.* **32**, 2319–2327 (2011).

66. The PyMOL Molecular Graphics System, Version 3 (Schrödinger, LLC).

67. M. Arndt, C. Alvadia, M.S. Straub, V. Clerico Mosina, C. Paulino, R. Dutzler, Structural basis for the activation of the lipid scramblase TMEM16F. *Nat. Commun.* **13**, 6692 (2022).

68. M. E. Falzone, A. Accardi, Reconstitution of proteoliposomes for phospholipid scrambling and nonselective channel assays. *Methods Mol. Biol.* **2127**, 207–225 (2020).

69. B.-C. Lee, G. Khelashvili, M. Falzone, A.K. Menon, H. Weinstein, A. Accardi, Gating mechanism of the extracellular entry to the lipid pathway in a TMEM16 scramblase. *Nat. Commun.* **9**, 3251 (2018).

70. B. K. Ho, F. Gruswitz, HOLLOW: Generating accurate representations of channel and interior surfaces in molecular structures. *BMC Struct. Biol.* **8**, 49 (2008).

71. M. Fonvielle, I. Li de La Sierra-Gallay, A.H. El-Sagheer, M. Lecerf, D. Patin, D. Mellal, C. Mayer, D. Blanot, N. Gale, T. Brown, H. van Tilbeurgh, M. Ethève-Quelquejeu, M. Arthur, The

structure of $FemX_{Wv}$ in complex with a peptidyl-RNA conjugate: mechanism of aminoacyl transfer from Ala-$tRNA^{Ala}$ to peptidoglycan precursors. *Angew. Chem. Int. Ed. Engl.* **52**, 7419–7422 (2013).
